# Metal−Organic Frameworks for Liquid Phase Applications

**DOI:** 10.1002/advs.202003143

**Published:** 2021-01-21

**Authors:** Anjaiah Nalaparaju, Jianwen Jiang

**Affiliations:** ^1^ Department of Chemical and Biomolecular Engineering National University of Singapore Singapore 117576 Singapore

**Keywords:** computations, liquid phase applications, metal−organic frameworks, nanoporous materials

## Abstract

In the last two decades, metal−organic frameworks (MOFs) have attracted overwhelming attention. With readily tunable structures and functionalities, MOFs offer an unprecedentedly vast degree of design flexibility from enormous number of inorganic and organic building blocks or via postsynthetic modification to produce functional nanoporous materials. A large extent of experimental and computational studies of MOFs have been focused on gas phase applications, particularly the storage of low‐carbon footprint energy carriers and the separation of CO_2_‐containing gas mixtures. With progressive success in the synthesis of water‐ and solvent‐resistant MOFs over the past several years, the increasingly active exploration of MOFs has been witnessed for widespread liquid phase applications such as liquid fuel purification, aromatics separation, water treatment, solvent recovery, chemical sensing, chiral separation, drug delivery, biomolecule encapsulation and separation. At this juncture, the recent experimental and computational studies are summarized herein for these multifaceted liquid phase applications to demonstrate the rapid advance in this burgeoning field. The challenges and opportunities moving from laboratory scale towards practical applications are discussed.

## Graphical abstract

There has been rapidly increasing interest to apply metal‐organic frameworks (MOFs) for liquid phase applications. Remarkable progress and significant advance in this field have been achieved over the last several years. Herein, the recent experimental and computational studies using MOFs for multifaceted liquid phase applications in energy, environmental and pharmaceutical sectors are summarized.

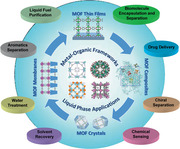

## 1 Introduction

The field of porous crystalline materials has received flourishing attention due to the possibilities to form well‐defined structures by molecular building blocks with spatial control over atomic scale.^[^ ^1^ ^]^ In the controllable porous space of these materials, guest molecules exhibit intriguing properties significantly different from bulk phase, thus leading to a wide range of applications. As a typical class of porous crystalline materials, zeolites comprise tetrahedral SiO_4_ and AlO_4_ units connected via corner‐sharing oxygen atoms to form highly ordered pores and crystalline skeletons. Due to the restriction of tetrahedral skeletons, zeolites can be produced with a limited number of structures. Since the first natural zeolite (stilbite) discovered in 1756, currently there are only 240 zeolites of distinct framework topologies.^[^ ^2^ ^]^ In the last two decades, metal−organic frameworks (MOFs) have emerged as a special class of porous crystalline materials.^[^ ^3^ ^]^ In remarkable contrast to zeolites, MOFs can be synthesized from an extremely wide range of inorganic clusters (e.g., trigonal, tetrahedral, and octahedral) and organic linkers (e.g., carboxylates, imidazolates, and tetrazolates). More importantly, judicious selection of building blocks allows for readily tailoring MOF structures and functionalities in a rational manner. To date, over 100 000 MOFs have been synthesized with their crystal structures deposited in the Cambridge Crystallographic Data Centre.^[^ ^4^ ^]^ Not only their structural diversity and tailorability, but the unique features like framework flexibility and chirality make MOFs very attractive than other classes of porous materials. Therefore, MOFs have been identified as a topical area in materials science and technology, and considered as versatile materials for many important potential applications such as storage, separation, drug delivery, sensing and catalysis.^[^ ^5^, ^6^, ^7^ ^]^

**Figure 1:**
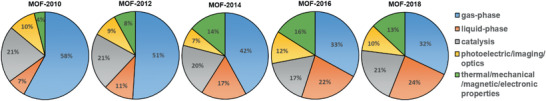
Percentage of papers presented at MOF conferences: MOF‐2010 (Marseille, France), MOF‐2012 (Edinburgh, UK), MOF‐2014 (Kobe, Japan), MOF‐2016 (California, USA), and MOF‐2018 (Auckland, New Zealand).

In early years of development, MOFs were primarily examined for gas storage of low‐carbon footprint energy carriers (e.g., H_2_ and CH_4_),^[^ ^8^, ^9^ ^]^ as largely inspired by the unprecedented surface areas and pore volumes. With inherent similarity to the molecular sieve characteristics of zeolites, meanwhile, there was also a great interest to apply MOFs as adsorbents or membranes for chemical separation, particularly the separation of CO_2_‐containing gas mixtures.^[^ ^10^, ^11^ ^]^ Impressive performance in gas storage and separation was achieved by tailor‐made MOFs with various atomic structures, topologies networks, pore dimensions, as well as surface chemistries.^[^ ^12^ ^]^ Recently, along with the traditional applications in gas phase, new promising avenues have emerged for MOFs including hydrocarbon separation, water treatment, solvent recovery, catalysis, luminescence, optics, thermal/mechanical/magnetic/electronic properties, etc.^[^ ^13^ ^]^ Such evolution is clearly evidenced from **Figure** **1** based on the papers presented in the past five MOF conferences. In the 1st one (2010), 58% of papers were focused on gas phase applications and this percentage dropped to 32% in the 5th one (2018). By contrast, more papers appeared for new avenues, particularly liquid phase applications rose from 7% in 2010 to 24% in 2018.

Liquids such as water, organic solvents and fuels are closely related to human activities and largely used in industries. In many applications, liquid phase operation is preferred over gas phase. For instance, heat‐sensitive compounds may be safely separated in a solution at room temperature, rather than being heated into gas or vapor. Many soluble compounds (e.g., amino acids, proteins, drugs, nucleic acids, antioxidants, carbohydrates, natural and synthetic polymers) can be dissolved in a solvent and purified, whereas gas phase separation is suitable for gaseous mixtures or volatile compounds.

One early application of MOFs in a liquid phase was the selective adsorption and inclusion of aromatic and non‐aromatic components (e.g., benzene, nitrobenzene, cyanobenzene and chlorobenzene) from solvents.^[^ ^14^ ^]^ However, actual implementation for liquid phase applications was impeded by low stability of MOFs. Due to the relatively weak coordination bonds between metals and ligands, a large number of MOFs are chemically unstable in solvents or under harsh conditions. This is why the early studies for MOFs were primarily focused on gas phase, despite the presence of impressive porosity.^[^ ^15^ ^]^ With the improved understanding towards metal‐ligand lability and MOF structural instability in water, acid/base and harsh solvent conditions, several strategies have been developed to design stable MOFs, particularly water stable MOFs. The strategies can be broadly categorized into de novo synthesis and postsynthetic modification. In de novo synthesis, hard/soft acid‐base principle is often used to choose the combination of metal‐ligand to reduce lability. For example, it is increasingly recognized that higher valence cations such as group‐IV metals can interact strongly with organic ligands; particularly zirconium‐based cluster is able to produce MOFs with superior hydrolytic, thermal and chemical stability. On the other hand, stable MOFs with low valence metals can be synthesized from nitrogen‐containing ligands such as imidazolates, pyrazolates, triazolates, and tetrazolates.^[^ ^16^ ^]^ In postsynthetic modification, appropriate functional groups are usually incorporated into existing MOFs or the coordination bonds of certain unstable building units are modified to enhance strength. Such modification to synthesize stable MOFs is not easily achievable through de novo route.^[^ ^17^ ^]^ Based on these strategies, more water‐ and solvent‐resistant MOFs have been successfully synthesized in the past several years. Therefore, as evidenced from the trend in Figure 1, there has been rapidly increasing interest in liquid phase applications.

**Figure 2:**
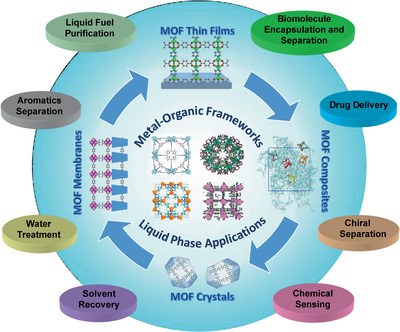
Liquid phase applications of MOFs.

As shown in **Figure** **2**, the potential liquid phase applications for MOFs are in a myriad range such as liquid fuel purification, aromatics separation, water treatment, solvent recovery, chemical sensing, chiral separation, drug delivery, biomolecule encapsulation and separation. It is worthwhile to note that some of these applications are beyond reach to other porous materials like zeolites. For example, drugs and biomolecules usually have diameters in a nanometer scale and are larger than the windows of most zeolites. Therefore, drug delivery and biomolecule encapsulation can hardly be achieved by zeolites; instead, biocompatible MOFs can be used. Moreover, the organic linkers of MOFs may exert favorable interactions with organic solvents, allowing for efficient solvent recovery. Asymmetric centers introduced into MOFs can be applied to enantioselective separation. Due to limited availability of pore size and inorganic nature, zeolites may not be appropriate for these specific applications. Moreover, MOFs offer an unprecedentedly large degree of tunability and structural diversity, as well as the wide range of chemical and physical properties. All these salient features facilitate the utilization of MOFs for liquid phase applications.

Compared with numerous reviews summarized for gas separation, there were only few reviews exclusively for liquid separation.^[^ ^18^, ^19^, ^20^ ^]^ These reviews discussed experimental studies on MOFs for liquid separation, not for general liquid phase applications. In this review, we attempt to summarize the recent experimental studies for a wide variety of liquid phase applications in energy, environmental and pharmaceutical sectors, including liquid fuel purification, aromatics separation, water treatment, solvent recovery, chemical sensing, chiral separation, drug delivery, biomolecule encapsulation and separation. In addition, representative computational efforts are also discussed. With ever‐growing mathematical algorithms and physical resources, computational studies have become an invaluable tool in materials characterization, screening and design. By comprehensively and critically summarizing the current state of MOFs for liquid phase applications, this review will provide systematic overview and facilitate the rational development of new MOFs in this important field.

## 2 Energy Sector

In the chemical and petrochemical industries, hydrocarbon is usually produced as a mixture and required to be separated. Traditional technology for hydrocarbon separation is distillation due to its simplicity and maturity in operation. However, distillation is energy intensive, particularly for the separation of liquids with closely boiling points and not suitable for temperature‐sensitive compounds. Adsorption and membrane technologies using MOFs provide superior alternatives to distillation. The recent development using MOFs for liquid phase hydrocarbon separation is summarized here, including liquid fuel purification and aromatics separation.

### 2.1 Liquid Fuel Purification

Gasoline and diesel are commonly used liquid fuels for transportation. Though gasoline has approximately equal portion of aliphatic and aromatic components, the major component in diesel is aliphatic hydrocarbons. The presence of impurities like sulfur‐ and nitrogen‐containing compounds (NCCs and SCCs) adversely affect fuel efficiency and cause environmental pollution.^[^ ^21^ ^]^ Moreover, these compounds can poison catalysts during combustion. Therefore, the impurities are required to be removed to produce cleaner liquid fuels. Hydrodenitrogenation and hydrodesulfurization are usually used to remove nonaromatic NCCs and SCCs, respectively. These techniques are less efficient for sterically hindered counterparts such as benzothiophene (BT), dibenzothiophene (DBT), 4,6‐dimethyldibenzothiophene (DMDBT), pyridines, and pyrroles.^[^ ^22^ ^]^ As an alternative, desulfurization and denitrogenation by adsorption in porous materials have attracted considerable attention due to low operating temperature and pressure, and the capability to achieve ultraclean fuels affordably. Traditional porous materials including zeolites and activated carbons have been tested for liquid fuel purification; however, they lack fast adsorption kinetics and high capacity, thus not widely used. The most important task currently is to develop adsorbents that are able to remove NCCs and SCCs with high selectivity from complex hydrocarbon mixtures.

**Figure 3:**
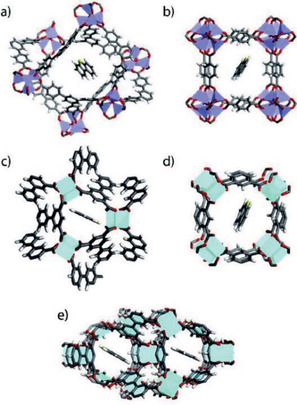
Locations of a DBT molecule in a) MOF‐177, b) MOF‐5, c) UMCM‐150, d) HKUST‐1, and e) MOF‐505. Reproduced with permission.^[^ ^23^ ^]^ Copyright 2008, American Chemical Society.

**Table** **1** lists the recent studies using MOFs for liquid fuel purification. Matzger's group examined the performance of five chemically diverse MOFs (HKUST‐1, UMCM‐150, MOF‐505, MOF‐5, and MOF‐177) for removing organosulfurs (BT, DBT, and DMDBT) in isooctane. These MOFs were found to exhibit excellent adsorption capacities, which are higher than NaY zeolite, indicating strong affinity between the frameworks and organosulfurs. UMCM‐150 and MOF‐505 have the highest affinity for DBT as evidenced in **Figure** **3** by the closest contact of DBT with UMCM‐150 and MOF‐505.^[^ ^23^ ^]^ Moreover, they carried out packed‐bed breakthrough experiments to remove DBT and DMDBT in model and authentic diesels, respectively. Exceptional amounts of solutions were desulfurized before breakthrough point. This study demonstrates the practicality of MOFs for the desulfurization of liquid fuels. Even in the presence of competitive aromatic compounds, these MOFs could still selectively adsorb DBT and DMDBT, in contrast to zeolites and activated carbons.^[^ ^24^ ^]^

**Table 1:** Liquid Fuel Purification

Mixture	MOF	Ref.
BT, DBT, DMDBT in iso‐octane	HKUST‐1, UMCM‐150, MOF‐505, MOF‐5, MOF‐177	^[^ ^23^ ^]^
DBT, DMDBT in diesel	HKUST‐1, UMCM‐150, MOF‐505, MOF‐5, MOF‐177	^[^ ^24^ ^]^
DBT, DMDBT in *i*‐octane	UMCM‐152, UMCM‐153	^[^ ^25^ ^]^
DBT, DMDBT in iso‐octane	UMCM‐150	^[^ ^26^ ^]^
Thiophene, tetrahydrothiophene in gasoline and diesel	Zn‐MOF, Cu‐DABCO‐MOF, Cu‐Iso‐MOF,Cu‐BTC	^[^ ^27^ ^]^
BT in *n*‐octane	MIL‐53(Al), MIL‐53(Cr), MIL‐47(V)	^[^ ^28^ ^]^
BT in *n*‐octane	CuCl_2_/MIL‐47	^[^ ^29^ ^]^
BT in *n*‐octane	PWA‐Cu‐BTC	^[^ ^30^ ^]^
BT in *n*‐octane, toulene/*n*‐octane	CuCl_2_/V‐BDC, CuCl_2_/Al‐BDC, CuCl_2_/Cr‐BDC	^[^ ^31^ ^]^
BT in *n*‐octane, toulene/octane mixture	IL/MIL‐101	^[^ ^32^ ^]^
BT, DBT in *n*‐octane	MOF dispersed in MIL‐100(Fe), CuBTC	^[^ ^33^ ^]^
Quinoline, indole in *n*‐octane, *p*‐xylene	MIL‐101(Cr)	^[^ ^34^ ^]^
Quinoline, indole in *n*‐octane, *p*‐xylene	PWA/MIL‐101(Cr)	^[^ ^35^ ^]^
Quinoline, indole in *n*‐octane, *p*‐xylene	GO/MIL‐101	^[^ ^36^ ^]^
Quinoline, indole in *n*‐octane	CuCl/MIL‐100(Cr)	^[^ ^37^ ^]^
Quinoline, indole in *n*‐octane	AlCl_3_/MIL‐100(Fe)	^[^ ^38^ ^]^
Quinoline, indole, pyrrole, and methylpyrrole in *n*‐octane	UiO‐66, UiO‐66‐NH_2_	^[^ ^39^ ^]^
Quinoline and indole in *n*‐otcane	UiO‐66‐NH‐SO_3_H, UiO‐66‐NH_2_	^[^ ^40^ ^]^
Quinoline and indole in *n*‐octane	Polyaniline‐encapsulated MIL‐101	^[^ ^41^ ^]^
Thiophene in naphtha	HKUST‐1, CPO‐27‐Ni, *rho*‐ZMOF, ZIF‐8, ZIF‐76	^[^ ^42^ ^]^
BT, DBT in iso‐octane	[Cu(L)_1/3_(H_2_O)]⋅8 DMA	^[^ ^43^ ^]^
BT, DBT, 3‐MT in *n*‐octane	Cu‐BTC, Cu‐BDC, Cr‐BTC, Cr‐BDC	^[^ ^44^ ^]^
BT, DBT in isooctane	IFMC‐16	^[^ ^45^ ^]^
Benzothiophene, benzothiazole, indole in isopropanol and heptane	MIL‐53	^[^ ^46^ ^]^
DBT in benzene (aromatic oil), *n*‐octane (aliphatic oil), and mixed oil	MOF‐5	^[^ ^47^ ^]^
DBT in *n*‐octane, DBT in benzene, *p*‐xylene, napthalene/*n*‐octane mixture	MIL‐101(Cr), PTA/MIL‐101(Cr)	^[^ ^48^ ^]^
Thiophene, BT, DBT in *n*‐octane	Fe_3_O_4_‐PAA‐MOF‐199	^[^ ^49^ ^]^
*N*‐compounds in light cycle oil	MIL‐101(Cr)	^[^ ^50^ ^]^
Aliphatic C_5_‐diolefins, mono‐olefins and paraffins	MIL‐96 and Cu_3_(BTC)_2_	^[^ ^51^ ^]^
Indole and carbazoles in toluene and heptane	MIL‐100(Fe, Cr, Al), MIL‐101(Cr), MIL‐47, MIL‐53, Cu_3_(BTC)_2_, CPO‐27(Ni,Co)	^[^ ^52^ ^]^
Indole and 1,2‐dimethylindole in heptane	MIL‐100(Al, Cr, Fe,V)	^[^ ^53^ ^]^
Pyridine, pyrrole, quinoline and indole in *n*‐octane	MIL‐96(Al), MIL‐53(Al), MIL‐101(Cr)	^[^ ^54^ ^]^
Quinoline, indole, DBT,4,6‐DMDBT, naphthalene in *n*‐octane	MIL‐101(Cr), MIL‐100(Fe), Cu‐BTC	^[^ ^55^ ^]^
Thiophene in *n*‐octane	Cu‐BTC or MIL‐101/PDMS MMM, Ag^+^@COF‐Pebax	^[^ ^56^, ^57^, ^58^, ^59^, ^60^ ^]^
Thiophene in iso‐octane	Zeolite beta @ HKUST‐1	^[^ ^61^ ^]^
Methanol/water	Cu(R‐GLA‐Me)(4,4’‐Bipy)_0.5_	^[^ ^62^ ^]^
C_1_‐C_5_ alcohols/water	ZIF‐8	^[^ ^63^ ^]^
Biobutanol/fermentation broth	ZIF‐8	^[^ ^64^ ^]^
2,5‐dimethylfuran and 2‐methylfuran in butanol	ZIF‐7, ZIF‐8, and ZIF‐71	^[^ ^65^ ^]^
Methanol, ethanol/water	Cu_2_(bza)_4_(pyz)/PDMS membrane	^[^ ^66^ ^]^
C_2_‐C_5_ alcohols/water	ZIF‐8/PMPS membrane	^[^ ^67^ ^]^
TP, BT and DBT	Cr‐MIL‐101‐SO_3_Ag	^[^ ^68^ ^]^
BT in *n*‐octane	IL@MIL‐101	^[^ ^69^ ^]^
DBT, BT	MOF‐74	^[^ ^70^ ^]^
Ethanol, *t*‐butanol, ester/water	MIL‐53 and MIL‐96 membranes	^[^ ^71^ ^]^
Alcohols/water, dimethyl carbonate/methanol	ZIF‐71 membrane	^[^ ^72^ ^]^
Butanol/water	ZIF‐8‐PDMS membrane	^[^ ^73^ ^]^
Ethanol/water	ZIF‐71/PDMS MMM	^[^ ^74^ ^]^
Ethanol/water	Sm‐DOBDC membrane	^[^ ^75^ ^]^
Methanol/MTBE	UiO‐66 membrane	^[^ ^76^ ^]^
Ethanol/water	Na‐*rho*‐ZMOF and Zn_4_O(bdc)(bpz)_2_	^[^ ^77^ ^]^ (sim.)
Ethanol/water	ZIF‐8, ‐25, ‐71, ‐90, ‐96, and ‐97	^[^ ^78^ ^]^ (sim.)
Ethanol/water	ZIF‐68, ‐69, ‐78, ‐79, and ‐81	^[^ ^79^ ^]^ (sim.)
Glycerol/methanol	IRMOF‐1	^[^ ^80^ ^]^ (sim.)

**Figure 4:**
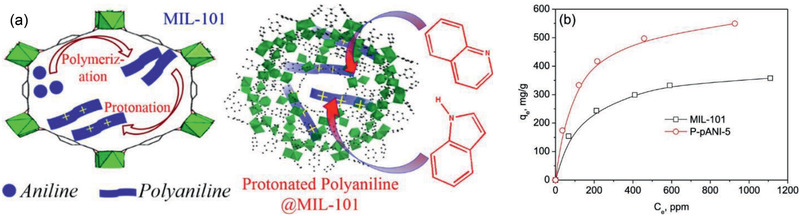
a) Schematic of N‐compounds adsorption in pANI‐loaded MIL‐101, b) adsorption isotherms of indole in MIL‐101 and P‐pANI‐5. Reproduced with permission.^[^ ^41^ ^]^ Copyright 2018, American Chemical Society.

Khan and Jhung systematically investigated the removal of NCCs and SCCs from liquid fuels by loading inorganic salts into MOFs as additional adsorption sites. Particularly, CuCl_2_ loaded MIL‐47 showed remarkable adsorption capacity for BT through the *π*‐complexation with Cu^+^ resulted from the reduction of loaded Cu^2+^ ions.^[^ ^29^ ^]^ Later, other compounds (e.g., AlCl_3_ and phosphotungstic acid) were also loaded. A stable Cu(I)‐compound [(Cu_2_(pyrazine)_2_(SO_4_)(H_2_O)_2_)*_n_*, denoted as CP] was employed to form composites in MIL‐100(Fe) and CuBTC. The prepared CP/MIL‐100(Fe) and CP/CuBTC composites were examined for the adsorptive removal of SCCs. The adsorption capacity of BT was found to increase with increasing CP content and the highest capacity was observed at a medium CP content denoted as CP(m)/MIL‐100(Fe). At the highest CP content, the capacity of BT was reduced as a result of an excessive degree of pore blocking. High adsorption capacity was also seen for DBT in CP(m)/MIL‐100(Fe). This study demonstrates the beneficial utilization of added active sites in porous MOFs for adsorption.^[^ ^33^ ^]^ Jhung and co‐workers further used MOF‐based composites to remove NCCs from liquid fuels. Specifically, polyaniline (pANI) encapsulated MIL‐101(Cr) was prepared via a ship‐in‐bottle strategy and applied in the adsorption of both basic quinoline and neutral indole. Record high adsorption capacities were obtained, as shown in **Figure** **4**, attributed to hydrogen bonding, acid–base interaction and cation−*π* interaction between the composites and adsorbates. It was suggested that protonated pANI‐encapsulated MOFs (P‐pANI) could be a new type of adsorbents for very efficient removal of NCCs from liquid fuels.^[^ ^41^ ^]^

**Figure 5:**
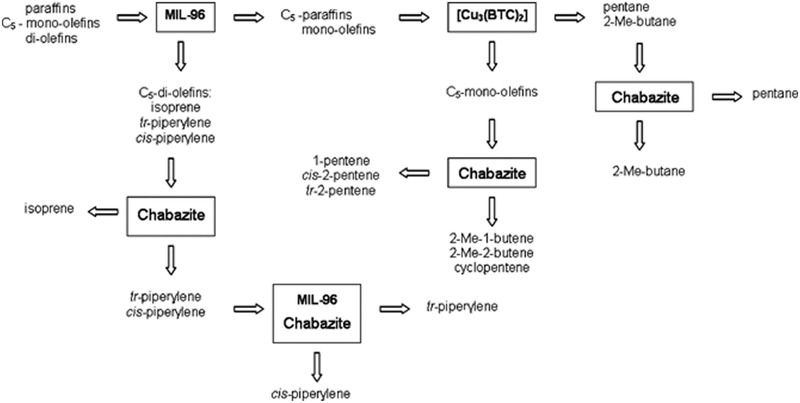
Flow scheme for the separation of C_5_‐cut from a steam cracker. Reproduced with permission.^[^ ^51^ ^]^ Copyright 2010, American Chemical Society.

De Vos's group investigated and compared the separation mechanisms of aliphatic C_5_‐diolefins, mono‐olefins and paraffins in two MOFs (MIL‐96 and Cu_3_(BTC)_2_) and two zeolites (CHA and LTA). These hydrocarbons represent a typical feed produced by a steam cracker. MIL‐96 and CHA were found to adsorb *trans*‐piperylene preferably from a mixture of three C_5_‐diolefin isomers with high separation factor. The capacity was higher compared with 5A zeolite as attributed to more efficient packing of *trans*‐isomer. In addition, CHA could separate *cis*‐piperylene and isoprene due to the size exclusion of branched isomers. Thus, CHA was shown suitable candidate for separating three C_5_‐diolefin isomers, as well as separating linear from branched mono‐olefins and paraffins. Cu_3_(BTC)_2_ was demonstrated to be able to separate C_5_‐olefins from paraffins. Interestingly, MIL‐96 was revealed to be the only material that could separate three C_5_‐diolefin isomers from C_5_‐mono‐olefins and paraffins. Based on these observations, a flow scheme illustrated in **Figure** **5** was devised for the separation of C_5_‐cut from a steam cracker. By a combination MOFs and zeolites, C_5_‐hydrocarons could be completely separated.^[^ ^51^ ^]^

**Figure 6:**
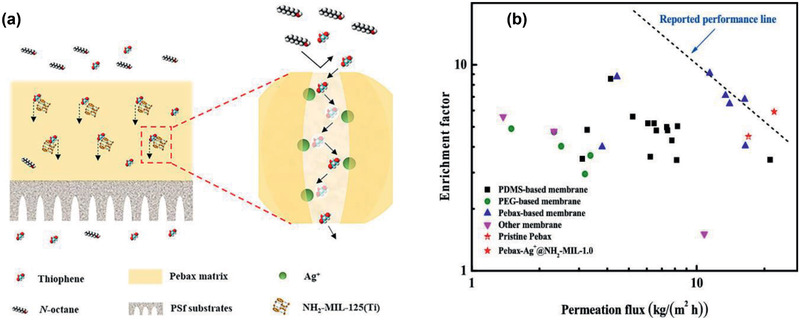
a) Thiophene sieving mechanism, b) pervaporative desulfurization performance of Pebax‐Ag^+^@NH_2_‐MIL‐1.0/polysulfone hybrid membrane and other membranes. Reproduced with permission.^[^ ^60^ ^]^ Copyright 2019, American Chemical Society.

In addition to being used as adsorbents, MOFs have been used as nanofillers into polymer membranes for the fabrication of mixed‐matrix membranes (MMMs). Cao and co‐workers added 8 wt% of Cu_3_(BTC)_2_ in polydimethylsiloxane (PDMS) membranes and tested for pervaporative removal of thiophene from model gasoline. The MOF particles were found to reduce permeation resistance and facilitate the selective transport of thiophene. In comparison with pristine PDMS membranes, the flux and enrichment factor were increased by 100% and 75%, respectively.^[^ ^56^ ^]^ In a separate study, MIL‐101(Cr)/PDMS membranes with 6% loading supported on polyvinylidene fluoride (PVDF) were examined for the desulfurization of model gasoline and thiophene/*n*‐octane mixture. Compared with the pristine membrane, increase of 136% and 38% was observed in flux and enrichment factor, respectively. The packing of PDMS chains was intervened by MIL‐101(Cr) particles, thus resulting in larger fractional free volume and enhanced flux.^[^ ^58^ ^]^ They further synthesized hybrid membranes of amine‐functionalized NH_2_‐MIL‐125(Ti) loaded with Ag^+^, coined as Ag^+^@NH_2_‐MIL‐125(Ti), and investigated for pervaporative desulfurization. It was found that the immobilized Ag^+^ could form reversible *π*‐complexation with thiophene and attain facilitated thiophene‐selective transport. On the other hand, the presence of Ag^+^ reduced the effective pore size and increased the diffusion selectivity of thiophene via a sieving mechanism illustrated in **Figure** **6**a. As a result, Pebax membrane with 1.0 wt% Ag^+^@NH_2_‐MIL‐125(Ti) exhibited an increase in flux and enrichment factor by 34% and 33%, respectively. As shown in Figure 6b, Pebax‐Ag^+^@NH_2_‐MIL‐1.0 was found to outperform over many other membranes reported in the literature. This study recommends the integration of multiple transport mechanisms by using MOFs as nanofillers to achieve superior separation performance.^[^ ^60^ ^]^

The depletion of fossil fuels has sparked enormous interest to develop alternative energy resources such as renewable biofuels. Compared with conventional fuels, biofuels are environmentally benign and carbon neutral with less emission of gaseous pollutants. As‐produced biofuels (e.g., bioethanol and biobutanol) contain a large amount of water and it is crucial to separate alcohols and water. Due to the formation of low‐boiling azeotropes, however, complete removal of water by distillation is not feasible. Moreover, alcohols are the minor components after fermentation and the separation of alcohols via distillation is energy intensive. In this context, a number of MOFs have been examined as adsorbents for biofuel purification.

Denayer and co‐workers used zeolitic imidazolate framework‐8 (ZIF‐8) for the adsorption of methanol, ethanol, propanol, butanol and acetone from aqueous mixtures. High capacity of butanol was observed, which significantly exceeds those in active carbon and silicalite. Concentrated butanol could be obtained by the regeneration of adsorption column with methanol and mild heating.^[^ ^63^ ^]^ Subsequently, they conducted batch and breakthrough measurements of ZIF‐8, active carbon, silicalite and SAPO‐34 for biobutanol purification. Other compounds present in a real ABE (acetone‐butanol‐ethanol) fermentation were found to have an insignificant effect on the adsorption of butanol in ZIF‐8, while SAPO‐34 exhibited high affinity for water and ethanol, but not for butanol. This study suggests that the combination of ZIF‐8 and SAPO‐34 might be attractive for the recovery and purification of biobutanol by adsorption.^[^ ^64^ ^]^

**Figure 7:**
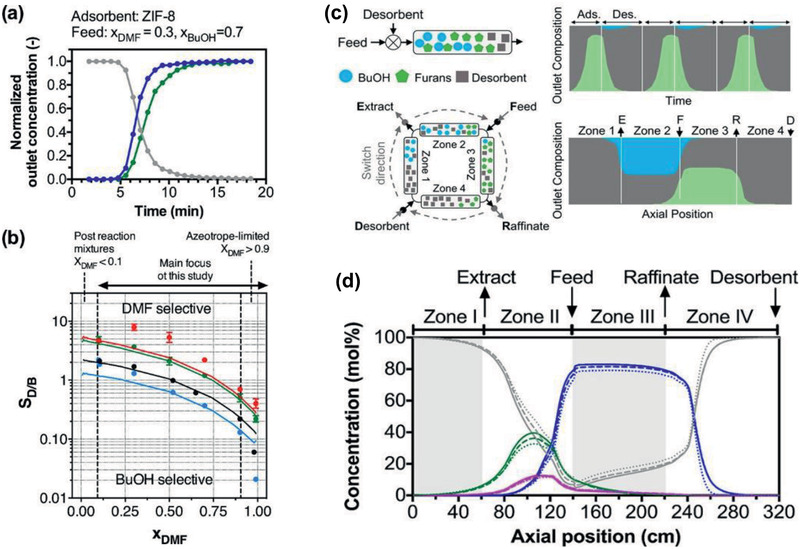
a,b) Typical result of a liquid breakthrough measurement using a binary feed containing DMF (green) and butanol (blue). Red: ZIF‐8. Green: ZIF‐71. Black: UiO‐66. Blue: FA‐UiO‐66. The solid curves represent IAST predictions. c,d) SMB and simulated liquid composition profiles along the axial position coordinate using ZIF‐8 and multicomponent Langmuir adsorption model. Reproduced with permission.^[^ ^65^ ^]^ Copyright 2019, American Chemical Society.

Recently, Nair and co‐workers utilized MOFs in a simulated moving bed (SMB) process to demonstrate the purification of solvents (e.g., butanol) and the concurrent production of furanics (e.g., 2,5‐dimethylfuran, 2,5‐DMF) from multicomponent biofuel reactor exit streams. Via adsorption measurements and ideal adsorbed solution theory (IAST) predictions, ZIF‐8 was revealed to have particularly favorable furan‐selective behavior. Preferable adsorption of DMF was verified by liquid phase breakthrough measurements as shown in **Figure** **7**a,b. An iterative refinement of multicomponent adsorption model as well as detailed dynamic SMB simulation, shown Figure 7c,d, were performed for a realistic multicomponent feed stream until the process converging to satisfy the extract and raffinate purity and recovery requirements. The ZIF‐based SMB was predicted to reach a productivity similar to those of current SMB processes for the production of petrochemical aromatics such as *p*‐xylene.^[^ ^65^ ^]^

Meanwhile, MOF‐based membranes have been investigated for biofuel purification. By dispersing [Cu_2_(bza)_4_(pyz)] (bza = benzoate, pyz = pyrazine) into PDMS to form a MMM, Takamizawa et al. attempted to separate alcohol/water mixtures by pervaporation. The membrane showed effective separation capability with separation factors of 5.6 and 4.7 for methanol/water and ethanol/water mixtures, respectively.^[^ ^66^ ^]^ Similarly, Liu et al. prepared a MMM by doping ZIF‐8 in a silicone rubber polymethylphenylsiloxane (PMPS) membrane and applied for the pervaporation separation of alcohols/water mixture. Both flux and selectivity were found to increase simultaneously with increasing ZIF‐8 and higher than those in the pristine PMPS membrane. Furthermore, the separation performance was enhanced with the carbon number of alcohols due to the increased adsorption capacity and selectivity.^[^ ^67^ ^]^

**Figure 8:**
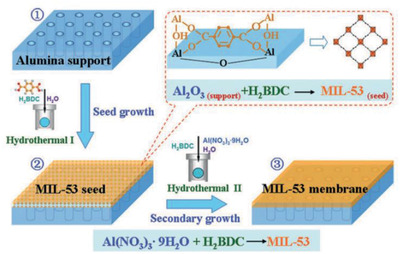
Reactive seeding method to prepare MIL‐53 membrane on an alumina support. Reproduced with permission.^[^ ^71^ ^]^ Copyright 2011, Royal Society of Chemistry.

Instead of MMMs, Jin and co‐workers developed a facile reactive seeding method to prepare continuous MIL‐53 and MIL‐96 membranes on an alumina support. As illustrated in **Figure** **8**, the porous support acted as an inorganic source reacting with organic precursor to grow a seeding layer. The prepared membranes were tested in pervaporation to separate the mixtures of organics (ethanol, *t*‐butanol and ester)/water. High selectivity was obtained and the membranes exhibited good stability.^[^ ^71^ ^]^ Dong and Lin prepared an organophilic ZIF‐71 membrane on a porous ZnO substrate for the pervaporation separation of alcohol/water and dimethyl carbonate (DMC)/methanol mixtures. Good performance was observed particularly for the separation of DMC from methanol, confirming the gate opening effect of ZIF‐71. This study demonstrates that ZIF‐71 membrane is promising for the separation of not only organics/water but also organics/organics mixtures.^[^ ^72^ ^]^

**Figure 9:**
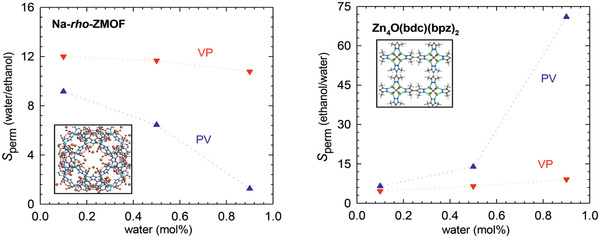
Permselectivities for water/ethanol mixtures in Na‐*rho*‐ZMOF and Zn_4_O(bdc)(bpz)_2_. Reproduced with permission.^[^ ^77^ ^]^ Copyright 2011, Royal Society of Chemistry.

There were a few simulation studies reported on biofuel purification. Jiang and co‐workers simulated the purification of ethanol/water mixtures in Na‐*rho*‐ZMOF and Zn_4_O(bdc)(bpz)_2_ membranes at both pervaporation (50 °C) and vapor permeation (100 °C) conditions. In hydrophilic Na‐*rho*‐ZMOF, water was found to be preferentially adsorbed over ethanol due to its strong interaction with the anionic framework and nonframework Na^+^ ions, and the adsorption selectivity of water/ethanol was higher at a lower composition of water. With increasing water composition, the diffusion selectivity of water/ethanol was observed to increase. In contrast, ethanol was adsorbed more in hydrophobic Zn_4_O(bdc)(bpz)_2_ as attributed to the favorable interaction with methyl groups, and the adsorption selectivity of ethanol/water was higher at a lower composition of ethanol. With increasing ethanol composition, the diffusion selectivity of ethanol/water increased slightly. As demonstrated in **Figure** **9**, the maximum permselectivity in Na‐*rho*‐ZMOF is about 12 by vapor permeation and Na‐*rho*‐ZMOF is preferable for biofuel dehydration. In Zn_4_O(bdc)(bpz)_2_, the maximum permselectivity is 75 by pervaporation and Zn_4_O(bdc)(bpz)_2_ is promising for biofuel recovery. This study presents microscopic insight into the separation of water/ethanol mixtures in hydrophilic and hydrophobic MOF membranes, imparts that hydrophobic MOF is superior to hydrophilic counterpart for biofuel purification.^[^ ^77^ ^]^

They subsequently simulated the adsorption and separation of ethanol/water mixtures in six ZIFs (ZIF‐8, ‐25, ‐71, ‐90, ‐96, and ‐97) with different functional groups. The adsorption isotherms of pure ethanol and water were predicted to agree fairly well with experimental data. Interestingly, the atomic charges in asymmetrically functionalized ZIF‐90, ‐96, and ‐97 were revealed to have important effect on adsorption, in remarkable contrast to symmetrically functionalized ZIF‐8, ‐25, and ‐71. The framework hydrophobicity as well as cage size were determined to govern ethanol/water selectivity, with ZIF‐8 exhibiting the highest selectivity among the six ZIFs.^[^ ^78^ ^]^ Later, a series of isoreticular ZIFs (ZIF‐68, ‐69, ‐78, ‐79, and ‐81) with GME topology but different organic linkers were also examined as adsorbents for ethanol/water separation. Among the five ZIFs, ZIF‐79 with hydrophobic –CH_3_ groups was shown to possess the highest adsorptive selectivity.^[^ ^79^ ^]^ These simulation efforts provide microscopic insights into the adsorption‐based separation of ethanol/water mixtures and would facilitate the development of new MOFs for biofuel purification.

To better understand biofuel/biochemical processing in a representative catalytic cycle, Li et al. conducted a simulation study on the adsorption and diffusion of glycerol/methanol mixtures in IRMOF‐1. The liquid mixtures were assumed to be ideal solutions and the Raoult's Law was used to estimate the fugacity of each component. The presence of a small amount of methanol was found to promote the adsorption of glycerol. The diffusion of glycerol was not affected by a low concentration of methanol, but enhanced with increasing methanol concentration. However, methanol diffusivity was found to be is a complex due to the interplay between the interaction with IRMOF‐1 framework and intermolecular steric effect.^[^ ^80^ ^]^

As discussed above, the application of MOFs for liquid fuel purification was first tested by adsorption and later by membrane operation. Majority of the studies listed in Table 1 were to explore MOFs with various textural properties and interaction sites. When used as adsorbents, MOFs are generally superior to other porous materials because of higher adsorption capacities, more selective and easier regeneration. This is attributed to the combination of specific interactions such as acid–base interaction, *π*–*π* complexation, van der Waals force, as well as H‐bonding with active sites in MOFs. The general trend for adsorption selectivity in MOFs is NCCs > SCCs > aromatics > aliphatics. MOF‐based membranes are relatively less investigated for liquid fuel purification. Integration of multiple transport mechanisms is revealed to be critical for membrane separation of compounds with similar properties. For biofuel purification, however, hydrophobic/hydrophilic interactions dominate membrane performance. In general, MOFs with unsaturated metal nodes offer an additional route for incorporating metals to facilitate interaction and permeation.

### 2.2 Aromatics Separation

While the above is focused on the purification of aliphatic liquid fuels, the separation of aromatics has also received considerable interest, particularly on xylenes and related aromatics (e.g., ethylbenzene and styrene). With an annual production of several million tons, xylenes are produced by the catalytic reforming of crude oil.^[^ ^81^ ^]^ After catalytic reforming, xylenes exist as part of BTEX aromatics (benzene, toluene, ethylbenzene and xylenes) in a colorless liquid state. Xylenes have three isomers namely *p*‐, *o*‐, and *m*‐xylene, and each isomer is useful as a raw material for the manufacturing of various high‐value added chemicals and polymers;^[^ ^82^ ^]^ therefore, it is crucial to separate these aromatics. Crystallization and adsorption are two main processes industrially practiced for aromatics separation. Between these two, adsorption is preferred because of a larger production rate and a significantly higher recovery at a lower cost. One major limitation with the industrially used adsorbents (BaX and KBaY) is the selectivity of *p*‐ over *m*‐xylene is not sufficiently high. Improving this selectivity is of paramount interest and industrial relevance.

**Figure 10:**
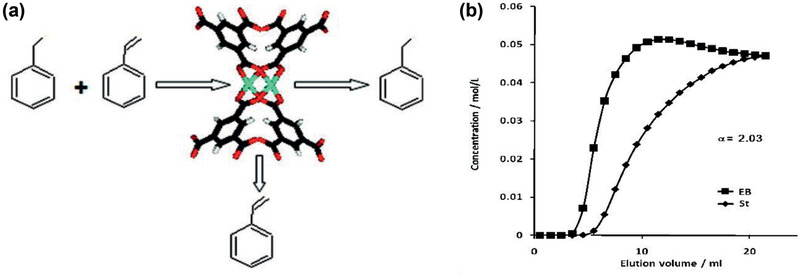
a) Ethylbenzene (EB)/styrene (St) separation on Cu_3_(BTC)_2_. b) breakthrough elution profiles of EB and St. Reproduced with permission.^[^ ^89^ ^]^ Copyright 2011, American Chemical Society.

**Table** **2** summarizes the studies on aromatics separation using MOFs as adsorbents or membranes. Early studies were conducted notably by De Vos and co‐workers. Among MIL‐47, MIL‐53, and Cu_3_(BTC)_2_ with similar pore aperture size, MIL‐47 was identified through breakthrough and chromatographic experiments to possess highest selectivity for the separation of C_8_ alkylaromatics (xylenes and ethylbenzene).^[^ ^83^ ^]^ They also combined batch, pulse chromatographic and breakthrough experiments with MIL‐53 to examine the selective adsorption of C_8_, C_9_, and C_10_ alkylaromatics (xylenes, ethylbenzene, ethyltoluenes and cymenes). MIL‐53 displayed a pronounced preference for *ortho*‐isomers from a feed of alkylaromatic isomers and appeared to be more effective to host large ethyltoluenes and cymenes than MIL‐47.^[^ ^84^ ^]^ Similar study was reported for the separation of olefins, alkylnaphthalenes and dichlorobenzenes in MIL‐47, MIL‐53 and Cu_3_(BTC)_2_. While Cu_3_(BTC)_2_ showed a remarkable preference for *cis*‐olefins over *trans*‐olefins via *π*‐complexation on its open metal sites, both MIL‐47 and MIL‐53 could separate 1,4‐dimethylnaphthalene from other alkylnaphthalene isomers, as well as *p*‐ and *m*‐dichlorobenzene. For alkylnaphthalenes, enthalpic interaction was revealed as an important factor governing selectivity; however, packing effect might dominate the selectivity of dichlorobenzenes.^[^ ^86^ ^]^ In a follow‐up study, the separation of ethylbenzene (EB)/styrene (St) on Cu_3_(BTC)_2_ was investigated to demonstrate the role of specific interactions between the *π*‐electrons of aromatic compounds and Cu^2+^ sites (**Figure** **10**a). Figure 10b shows the breakthrough elution profiles of EB and St with a separation factor of ≈ 2. A roll‐up‐effect was also observed due to the displacement of less preferred EB by more strongly adsorbing St.^[^ ^89^ ^]^ They also examined the capability of three MOFs (MIL‐125, MIL‐125‐NH_2_ and CAU‐1‐NH_2_) for the separation of xylenes, ethyltoluenes and cymenes dissolved in heptane. *Para*‐selective adsorption was observed in the three MOFs, particularly the selectivity of *p*‐cymene over *m*‐cymene was measured to be >109 in MIL‐125‐NH_2_. The experimental data were in accord with simulation predictions; however, the simulations were conducted in a vapor phase, not a full representation of liquid separation.^[^ ^91^ ^]^

**Table 2:** Aromatics Separation

Aromatics	MOF	Ref.
Xylenes, ethylbenzene	MIL‐47, MIL‐53, Cu_3_(BTC)_2_	^[^ ^83^ ^]^
Xylenes, ethylbenzene, ethyltoluenes, cymenes	MIL‐53	^[^ ^84^ ^]^
Xylenes, ehtyltoluenes, dichlorobenzenes, toluidines, cresols	MIL‐47	^[^ ^85^ ^]^
Alkylnaphthalenes, dichlorobenzenes	MIL‐47, MIL‐53, Cu_3_(BTC)_2_	^[^ ^86^ ^]^
Ethylbenzene/styrene, *p*‐ethyltoluene/*p*‐methylstyrene	Cu_3_(BTC)_2_/silica composites	^[^ ^87^ ^]^
Ethylbenzene, styrene, toluene, *o*‐xylene	MIL‐47, MIL‐53	^[^ ^88^ ^]^
Ethylbenzene/styrene, vinyltoluenes/ethyltoluenes	Cu_3_(BTC)_2_	^[^ ^89^ ^]^
Methylnaphthalenes, dimethylnaphthalene, naphthalene	Cu_2_(bdc)_2_(dabco)	^[^ ^90^ ^]^
Xylenes, ethyltoluenes, cymenes	MIL‐125, MIL‐125‐NH_2_, CAU‐1‐NH_2_	^[^ ^91^ ^]^
*n*‐propylbenzene, cumene	MIL‐47	^[^ ^92^ ^]^
BTEX	MIL‐53	^[^ ^93^ ^]^
Ethylbenzene, *p*‐xylene, *o*‐xylene	MIL‐53(Al)	^[^ ^94^ ^]^
Xylenes batch adsorption	Al‐MOF, CAU‐13	^[^ ^95^ ^]^
C_8_ alkyaromatics, dihydroxybenzene isomers, butanol isomers	Cu(CDC)	^[^ ^96^ ^]^
*cis/trans‐*1,3‐dimethylcyclohexane and 4‐ethylcyclohexanol	MIL‐125(Ti)	^[^ ^97^ ^]^
Xylenes, ethylbenzene, dichlorobenzene, chlorotoluenes, styrene	MIL‐101	^[^ ^98^ ^]^
Xylenes, dichlorobenzene, chlorotoluenes, toluene, naphthalene, anthracene, phenanthrene, pyrene, etc.	MIL‐53	^[^ ^99^, ^100^ ^]^
Nitroaniline, aminophenol, naphthol isomers, sulfadimidine, and sulfanilamide	MIL‐101(Cr)	^[^ ^101^ ^]^
BTE, naphthalene and 1‐chloronaphthalene; aniline, acetanilide, 2‐nitroaniline and 1‐naphthylamine, isomers of chloroaniline or toluidine	MIL‐100	^[^ ^102^ ^]^
Neutral polycyclic aromatic hydrocarbons, acetanilide, 4‐fluoroaniline, 2‐nitroaniline, 1‐naphthylamine, resorcinol, *m*‐cresol, 2,6‐dimethylphenol, 2,6‐dichlorophenol, 1‐naphthol, 1‐methylnaphthalene, 1‐chloronaphthalene	UiO‐66‐poly(MAA‐co‐EDMA)	^[^ ^103^ ^]^
Endocrine‐disrupting chemicals, pesticides	ZIF‐8/silica	^[^ ^103^ ^]^
Polycyclic aromatic hydrocarbons, phenols, anilines	COF TpPa‐MA	^[^ ^104^ ^]^
Xylene, chlorotoluene and cymene	MIL‐101(Cr) poly(BMA‐EDMA)	^[^ ^105^ ^]^
Benzene, ehtylbenzene, styrene, naphthalene, anthracene, phenanthrene, pyrene	MOF‐5, Cu_3_(BTC)_2_	^[^ ^106^ ^]^
Ethylcinnamate, styrene, *n*‐heptane, *i*‐octane, dichloromethane	MOF‐5, Cu_3_(BTC)_2_, MIL‐101, DUT‐5, 6, 7, 9, ZIF‐8, Zn_4_O(btb)_2_, Zn_2_(bdc)_2_(dabco)	^[^ ^107^ ^]^
Xylenes	UiO‐66	^[^ ^108^ ^]^
Xylenes	ZIF‐8	^[^ ^109^ ^]^
3ʹ‐hydroxyacetophenone, 3‐(1‐hydroxy phenyl) ethanol, 3‐[1‐(methylamino)ethyl] phenol, 3‐[1‐(dimethylamino)ethyl] phenol	HKUST‐1, MIL‐47, ZIF‐8	^[^ ^110^ ^]^
Ethylbenzene, styrene	HKUST‐1	^[^ ^111^ ^]^
Substituted benzenes and polycyclic aromatic hydrocarbons	UiO‐66	^[^ ^112^ ^]^
Xylene, dichlorobenzene, chlorotoluene and nitroaniline isomers	MIL‐53	^[^ ^113^ ^]^
*n*‐hexane, benzene, mesitylene	ZIF‐8 membrane	^[^ ^114^ ^]^
Xylene isomers	Zn_2_(BDC)_2_DABCO membrane	^[^ ^115^ ^]^
*p*‐xylene, *o*‐xylene	MOF‐5 membrane	^[^ ^116^ ^]^
Toluene, xylene, and 1,3,5‐triiso propylbenzene	MOF‐5 membrane	^[^ ^117^ ^]^
Toluene/*n*‐heptane, benzene/cyclohexane	MOP/W3000 MMM	^[^ ^118^ ^]^
Toluene/*n*‐heptane	Cu_3_(BTC)_2_/PVA MMM	^[^ ^119^ ^]^
Toluene/*n*‐heptane, benzene/cyclohexane	[MOP‐X, X = SO_3_Na*_n_*H*_m_*, OH and *t*Bu]/W3000	^[^ ^120^ ^]^
Toluene/*iso*‐octane, benzene/cyclohexane, toluene/cyclohexane and toluene/*n*‐heptane	Co(HCOO)_2_/ PEBA MMM	^[^ ^121^ ^]^
Substituted benzenes and polycyclic aromatic hydrocarbons	UiO‐66‐NH2,UiO‐67	^[^ ^122^ ^]^
Divinylbenzene and ethylvinylbenzene isomers	MIL‐53, MIL‐100	^[^ ^123^ ^]^
Nitrobenzenes, nitrotoluenes, triphenyl benzene and tris(4‐bromobenzyl)benzene	UMCM‐310	^[^ ^124^ ^]^
Cyclohexene or benzene from cyclohexane	HKUST‐1‐silica composite	^[^ ^125^ ^]^
C_8_‐isomers, dichlorobenzene isomers, styrene and ethylbenzene	UiO‐67/silica composite	^[^ ^126^ ^]^
Xylenes and ethylbenzene	Co_2_(dobdc), Co_2_(m‐dobdc)	^[^ ^127^ ^]^
Xylenes	MIL‐101	^[^ ^128^ ^]^ (sim.)
Xylenes	14 MOFs	^[^ ^129^ ^]^ (sim.)
Xylenes	MIL‐53(Al)	^[^ ^130^ ^]^ (exp./sim.)
Xylenes	2500 MOFs	^[^ ^131^ ^]^ (sim./exp.)
Xylenes	MIL‐160 on PDA‐modified *α*‐Al_2_O_3_ support	^[^ ^132^ ^]^ (exp./sim.)

**Figure 11:**
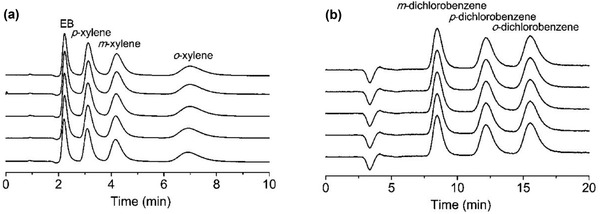
HPLC chromatograms on MIL‐101 slurry‐packed column for the separation of a) xylenes and ethylbenzene b) dichlorobenzenes. Reproduced with permission.^[^ ^98^ ^]^ Copyright 2011, American Chemical Society.

Based on high‐performance liquid chromatography (HPLC), Yang and Yan applied slurry‐packed MIL‐101 column for the separation of xylenes, ethylbenzene, dichlorobenzene, chlorotoluenes and styrene. MIL‐101 was found to offer high affinity for *ortho*‐isomer, allowing fast and selective separation of isomers as shown in **Figure** **11**. For xylenes, dichlorobenzene and chlorotoluenes, the separation was controlled by entropy effect; while enthalpy change played a dominant role in ethylbenzene and styrene.^[^ ^98^ ^]^ Subsequently, they used MIL‐53 as a stationary phase and explored the separation of xylenes, dichlorobenzene, chlorotoluenes, toluene, naphthalene, anthracene, phenanthrene and pyrene, as well as polar analytes. High resolution and good precision were obtained and the effects of mobile phase composition, injected sample mass and temperature were examined. The great potential of HPLC separation using MOF‐packed columns was envisioned from the combined mechanisms of size‐exclusion, shape selectivity and hydrophobicity.^[^ ^99^, ^100^ ^]^

Matzger and co‐workers evaluated MOF‐5 and Cu_3_(BTC)_2_ for the shape and size selective separation of benzene, ehtylbenzene, styrene, naphthalene, anthracene, phenanthrene, pyrene, 1,3,5‐triphenylbenzene and 1,3,5,‐tris(4‐bromophenyl)benzene. Excellent performance was achieved based on a combination of adsorption and molecular sieving.^[^ ^106^ ^]^ Adsorption of ethyl cinnamate and styrene from solvents was reported by Henschel et al. in various MOFs such as MOF‐5, Cu_3_(BTC)_2_, MIL‐101, DUT‐5,6,7,9, ZIF‐8, Zn_4_O(btb)_2_, and Zn_2_(bdc)_2_(dabco). They found that adsorption capacity was strongly dependent of framework chemistry, pore size and shape, as well as pore volume. Additionally, the size and polarity of solvent also appeared to have significant effects.^[^ ^107^ ^]^ UiO‐66 was studied by Moreira et al. for the separation of xylenes using *n*‐heptane as an eluent. By pulse experiment, UiO‐66 was shown to preferentially adsorb *o*‐xylene. From binary and multicomponent breakthrough experiments, the selectivities were determined to be 1.8 and 2.4 for *o*‐xylene over *m*‐xylene and *p*‐xylene, respectively.^[^ ^108^ ^]^ Peralta et al. presented the separation of xylene isomers in ZIF‐8 on the basis of molecular sieving mechanism. The highest capacity was seen for *p*‐xylene because of the smallest size, whereas *o*‐xylene with the largest size exhibited the least capacity. The rate of adsorption was also governed by the size of xylene, decreasing from *p*‐ to *m*‐ and to *o*‐xylene. In liquid phase breakthrough experiments, the separation efficiency was lower compared with that in gas phase. The quite dispersed breakthrough fronts indicated slow diffusion of xylenes into the sodalite cage of ZIF‐8. In addition, structural analysis suggested that the diffusion might occur via the transitory deformation of pore aperture in ZIF‐8.^[^ ^109^ ^]^

**Figure 12:**
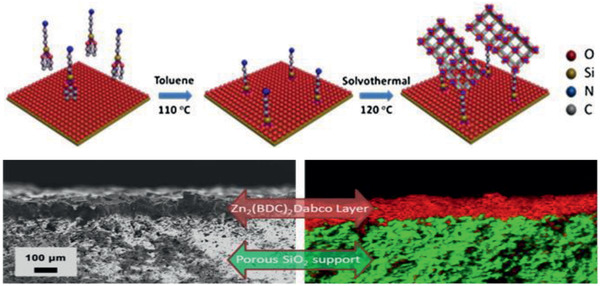
(Top) Zn_2_(BDC)_2_DABCO membrane preparation by a secondary growth approach. (Bottom) Cross‐section SEM image and EDS map of Zn_2_(BDC)_2_DABCO membrane grown after four cycles. Reproduced with permission.^[^ ^115^ ^]^ Copyright 2013, Elsevier.

In addition to adsorptive and chromatographic separation, recent endeavor has applied MOF membranes for aromatics and aromatic/aliphatic mixtures. Caro and co‐workers prepared a thin‐film composite ZIF‐8 membrane on *α*‐Al_2_O_3_ support with ZIF‐8 layer of about 15 µm thickness continuously and densely grown. The membrane was evaluated for the pervaporation separation of *n*‐hexane/benzene and *n*‐hexane/mesitylene mixtures. The measured separation factor was lower than the predicted ideal permselectivity for *n*‐hexane/benzene as the mobile component *n*‐hexane was blocked by less mobile benzene. For *n*‐hexane/mesitylene, however, *n*‐hexane flux was increasingly reduced by pore entrance blocking with increasing mesitylene concentration.^[^ ^114^ ^]^ Kang et al. synthesized a continuous‐growth Zn_2_(BDC)_2_DABCO membrane on a porous SiO_2_ substrate. To prepare MOF membrane by a second growth approach, as illustrated in **Figure** **12**, the surface of porous SiO_2_ was first modified with a linker 3‐aminopropyl‐triethoxysilane (APTES). The coordination of NH_2_ groups in APTES and zinc sites in the seed crystals increased the binding force between membrane and support. The membrane performance for the separation of xylene isomers was investigated. In contrast with zeolite membrane, this MOF membrane showed greater permeability for *o*‐xylene and *m*‐xylene with larger kinetic diameters due to the selective adsorption on the framework.^[^ ^115^ ^]^

**Figure 13:**
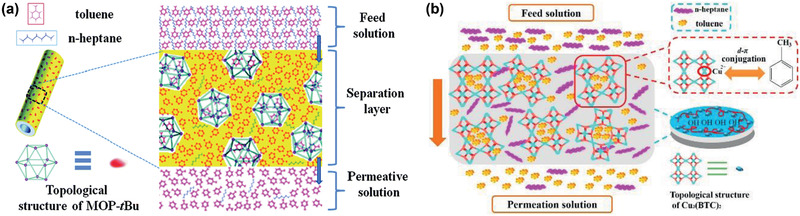
Toluene/*n*‐heptane separation through a) MOP/W3000, b) Cu_3_(BTC)_2_/PVA hybrid membranes. a) Reproduced with permission.^[^ ^118^ ^]^ Copyright 2014, Royal Society of Chemistry. b) Reproduced with permission.^[^ ^119^ ^]^ Copyright 2015, Elsevier.

On the other hand, Li and co‐workers embedded soluble metal‐organic polyhedral (MOPs) into W3000 polymer to produce hybrid membranes for the PV separation of aromatic/aliphatic mixtures. For toluene recovery from its equimolar *n*‐heptane solution, a separation factor of 19.0 and a flux of 220.5 g m^−2^ h^−1^ were obtained at 4.8 wt% MOP loading and comparable with other reported membranes. As illustrated in **Figure** **13**a, the embedding of MOP into W3000 could create preferential pathways for toluene due to high affinity of the coordinative unsaturated sites or *π*‐bond sites in MOPs. Moreover, the cavities of MOP were blocked by adsorbed toluene on these active sites located near MOP pore windows, thus restricting the entrance of *n*‐heptane even though *n*‐heptane is smaller than toluene. This resulted in increase of both flux and separation factor with increasing MOP loading. It was found that toluene transport favored through W3000 layer due to the presence of polar groups in MOP. Overall, the separation performance of hybrid MOP/W3000 membranes were significantly improved.^[^ ^118^ ^]^ Subsequently, by using Cu_3_(BTC)_2_ as a filler in poly(vinyl alcohol) (PVA), another MOF/polymer hybrid membrane was prepared and tested for the separation of 50 wt% toluene/*n*‐heptane mixtures through pervaporation. The separation factor and flux were improved upon comparison with pristine PVA membrane, specifically, from 8.9 and 14 g m^−2^ h^−1^ to 17.9 and 133 g m^−2^ h^−1^, respectively. The transport mechanism of permeating components in the selective layer of Cu_3_(BTC)_2_/PVA membrane was proposed as shown in Figure 13b. Presumably, the empty 3d‐orbitals of Cu^2+^ ion could contribute to d‐*π* conjugation interaction between the unsaturated metal ion and aromatic molecule, which resulted in a solubility difference relative to aliphatic compounds in the membrane. Furthermore, the selective layer in Cu_3_(BTC)_2_/PVA might bind toluene more strongly than *n*‐heptane, thus improving separation performance.^[^ ^119^ ^]^

The MOP/W3000 and Cu_3_(BTC)_2_/PVA hybrid membranes by Li and co‐workers^[^ ^118^, ^119^ ^]^ showed better separation capability than the pristine polymer counterparts; nevertheless, their performance was found not always improved upon increasing MOF loading. To provide in‐depth understanding, they further synthesized Co(HCOO)_2_/polyether‐block‐amide (PEBA) membranes for the separation of toluene and *n*‐heptane mixtures. When Co(HCOO)_2_ loading was increased from 1 to 8 wt%, the separation factor initially increased and then decreased, while the flux kept increasing; at a loading of 4 wt%, the highest separation factor was achieved. When the loading was low, Co(HCOO)_2_ particles would contribute to the enhanced interaction between toluene and membrane, and increase fractional free volume. Thus the flux and separation factor were increased. At excessive loading, however, the particles would aggregate and form certain defects on the membrane surface. Consequently, the flux was increased while the separation factor was decreased.^[^ ^121^ ^]^

**Figure 14:**
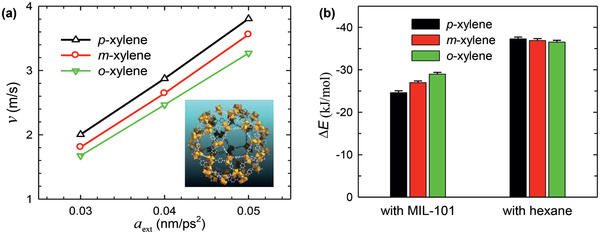
a) Transport velocities of xylene isomers versus external force. b) Interaction energies of xylene isomers with MIL‐101 and hexane, respectively. Reproduced with permission.^[^ ^128^ ^]^ Copyright 2013, American Chemical Society.

Along with the above‐discussed experimental studies, simulations were performed for xylene separation by MOFs. Jiang and co‐workers reported a molecular dynamics (MD) simulation study to examine liquid separation of xylenes with hexane as a mobile phase and MIL‐101 as a stationary phase.^[^ ^128^ ^]^ As demonstrated in **Figure** **14**, the transport velocities of three xylenes isomers were predicted to rise as *p*‐xylene > *m*‐xylene > *o*‐xylene, consistent with experiment. The interactions of *p*‐, *m*‐, and *o*‐xylenes with MIL‐101 (∆*E*_framework_) increased from −24.6, −27.0 to −29.0 kJ mol^−1^, while the interactions with hexane (∆*E*_solvent_) decreased from −37.3, −36.9 to −36.5 kJ mol^−1^. Therefore, *p*‐xylene interacted the most weakly with MIL‐101 but the most strongly with hexane, thus exhibiting the fastest transport velocity. The ΔΔ*E* ( = ∆*E*_solvent_ − ∆*E*_framework_) were estimated to be −12.7, −9.9, and −7.5 kJ mol^−1^ for *p*‐, *m*‐, and *o*‐xylenes, respectively, with the same decreasing trend as the transport velocities. The difference in ΔΔ*E* among the three xylenes was greater than in either Δ*E*_framework_ or Δ*E*_solvent_, suggesting a cooperative contribution of solute‐framework and solute‐solvent interactions to the observed separation. In accord with energetic analysis, structural analysis based on radial distribution functions further indicated the closest contact of *o*‐xylene to MIL‐101.^[^ ^128^ ^]^

Based on grand‐canonical Monte Carlo (GCMC) simulations, Torres‐Knoop et al. examined 14 MOFs, as well as 12 zeolites, for the adsorption and separation of xylene isomers, highlighted the crucial role of channel dimension in governing separation. With commensurate stacking, MAF‐X8 was identified as a promising candidate for *p*‐xylene adsorption.^[^ ^129^ ^]^ Agrawal et al. combined experiment and computation to study the adsorption and separation of xylene isomers by flexible MIL‐53 with a focus on the effects of different metals (Al, Cr and Ga). Compared with MIL‐53(Cr) and MIL‐53(Ga), MIL‐53(Al) was found to have the highest gravimetric saturation capacity and the lowest transition pressure for *o*‐xylene. In accord with this trend, high *o*‐xylene selectivity was obtained with MIL‐53(Al) being the most selective and the simulation showed good overall agreement with experiment.^[^ ^130^ ^]^ From computational screening of ≈ 2500 MOFs, Sholl and co‐workers predicted two MOFs (MOF‐48 and MIL‐140B) with comparable performance to zeolite BaX, which is currently used in industrial *p*‐xylene enrichment from C_8_ aromatics mixture; then they synthesized the top performing MOFs and evaluated through breakthrough adsorption experiment.^[^ ^131^ ^]^

**Figure 15:**
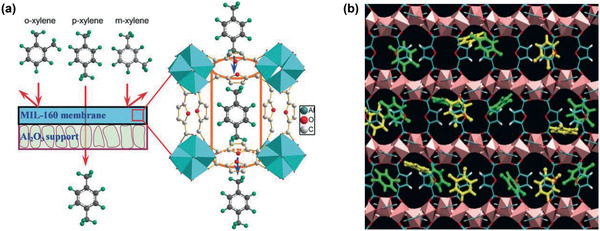
a) Schematic representation of the separation of *p*‐xylene from *o*‐xylene and *m*‐xylene through MIL‐160 membrane. b) Simulation snapshot of *p*‐xylene (green) and *o*‐xylene (yellow) adsorbed in MIL‐160 at 75 °C and 10 kPa, showing an enrichment of *p*‐xylene over *o*‐xylene due to the stronger interaction between *p*‐xylene and MIL‐160. Reproduced with permission.^[^ ^132^ ^]^ Copyright 2018, Wiley‐VCH.

Huang and co‐workers reported the selective separation of *p*‐xylene from its isomers by pervaporation using a highly selective and stable MIL‐160 membrane. As illustrated in **Figure** **15**a, the suitable pore size (0.5–0.6 nm) of MIL‐160 was expected to allow *p*‐xylene to pass through, while excluding bulkier *o*‐/*m*‐xylenes. However, it was found that both *m*‐xylene and *o*‐xylene could enter into the channels of MIL‐160. This was attributed to the effect of framework flexibility and further supported by molecular simulation. As illustrated by the simulation snapshot in Figure 15b, *p*‐xylene was shown to be predominantly adsorbed over *o*‐xylene in MIL‐160 due to its stronger interaction with MIL‐160 than *o*‐xylene. As a result of both higher adsorption and diffusion selectivities of *p*‐xylene over *o*‐xylene, *p*‐xylene was observed to possess a higher permeability than *o*‐xylene through the membrane. This study reveals MIL‐160 membrane as a promising candidate for the separation of xylene isomers by pervaporation. ^[^ ^132^ ^]^

Predominant studies listed in Table 2 for aromatics separation were focused on selective adsorption of isomeric aromatics. These studies indicate that the underlying mechanisms for aromatics separation are a complex interplay of many factors including size‐, shape‐ and topology‐based exclusion, entropic and packing effect; meanwhile, aromatics‐MOF interaction like *π*‐complexation, enthalpic effect, solvent polarity and hydrophobicity all come into play for separation. Engaging such a large variation of strategies to tune separation performance is not possible for traditional porous materials. Moreover, the unique structural flexibility of MOFs due to breathing or gate‐opening (e.g., MIL‐53) provides additional tunability for separation. The implementation of MOF‐based membranes in aromatics separation is in its incipiency, primarily by pervaporation.^[^ ^113^, ^114^, ^115^, ^116^, ^117^, ^118^, ^119^, ^120^ ^]^

## 3 Environmental Sector

### 3.1 Water Treatment

Water scarcity has been a global concern and how to efficiently supply fresh water is a topical issue. Meanwhile, substantial amount of contaminants such as metal ions and organic compounds (e.g., aromatics, dyes, and pharmaceuticals) have been introduced into water due to increasing world population and economic development. It is indispensable to remove these contaminants from water, thus minimizing health and environmental risks. A handful of techniques have been proposed for water treatment such as ion exchange, adsorption and membrane filtration using inorganic and polymeric materials.^[^ ^133^ ^]^ However, these materials usually possess small pores, thus leading to slow kinetics and low capacity.

**Figure 16:**
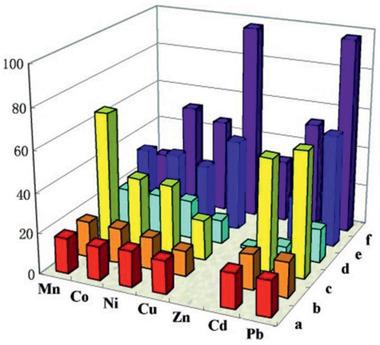
Percentages of adsorbed metal ions and exchanged Zn^2+^ ions by Zn(4,4’‐bpy)_2_‐(FcphSO_3_)_2_ from the solutions at different concentrations of Mn^2+^, Co^2+^, Ni^2+^, Cu^2+^, Zn^2+^, Cd^2+^, and Pb^2+^. The red, orange, and yellow columns represent the percentages of exchanged Zn^2+^ ions from solutions at 10, 100, and 1000 µg mL^−1^, respectively, whereas the brown, blue, and cyan columns represent the percentages of adsorbed metal ions from solution at 10, 100, and 1000 µg mL^−1^, respectively. Reproduced with permission.^[^ ^135^ ^]^ Copyright 2008, Wiley‐VCH.

MOFs have been examined to remove ions and organics as summarized in **Table** **3**. Mi et al. reported a zinc ferrocenyl sulfonate MOF, Zn(4,4’‐bpy)_2_‐(FcphSO_3_)_2_ as a molecular aspirator for the removal of toxic metal ions (Pb^2+^, Cd^2+^, Cu^2+^, Mn^2+^, Co^2+^, Ni^2+^, and Zn^2+^). They observed that a powdered sample could remove metal ions mainly by sorption; however, ion exchange was observed in a single crystalline sample. **Figure** **16** plots the percentages of adsorbed metal ions (columns a–c) and exchanged Zn^2+^ ions (columns d–f) from solutions at different concentrations of metal ions. With increasing ion concentration, the percentage of exchanged Zn^2+^ ions and the quantity of adsorbed metal ions were found to increase. Thus, metal ion sorption occurred mainly in a dilute solution, whereas both ion sorption and exchange were present in a concentrated solution. This study implies the complex mechanisms involved in the removal of metal ions by a MOF.^[^ ^135^ ^]^

**Table 3:** Removal of ions and organics from water

Species	MOF	Ref.
Mn^2+^, Zn^2+^, Pb^2+^	Cd(bpp)_2_‐(O_3_SFcSO_3_), Cd(bpy)_2_‐(O_3_SFcSO_3_)	^[^ ^134^ ^]^
Mn^2+^, Zn^2+^, Pb^2+^, Cd^2+^	Zn(4,4’‐bpy)_2_‐(FcphSO_3_)_2_	^[^ ^135^ ^]^
Li^+^‐Cs^+^, Mg^2+^‐Ba^2+^, Mn^2+^‐Zn^2+^, Fe^3+^	NaLa(H_4_L)	^[^ ^136^ ^]^
Cd^2+^, Hg^2+^	PCN‐100, PCN‐101	^[^ ^137^ ^]^
Cd^2+^	Cu_3_(BTC)_2_‐SO_3_H	^[^ ^138^ ^]^
CH_3_Hg^+^, Hg^2+^	BioMOF	^[^ ^139^ ^]^
Hg^2+^	Thiol‐functionalized HKUST‐1	^[^ ^140^ ^]^
As^5+^	Fe‐BTC	^[^ ^141^ ^]^
Hg^2+^, Bi^3+^, Zn^2+^, Pb^2+^, Cd^2+^	UiO‐66	^[^ ^142^ ^]^
Hg^2+^	Thiol‐laced UiO‐66	^[^ ^143^ ^]^
Hg^2+^, Pb^2+^	Fe‐BTC/PDA composite	^[^ ^144^ ^]^
Cu^2+^, Ni^2+^	UiO‐66(Zr)‐2COOH	^[^ ^145^ ^]^
Cu^2+^, Co^2+^	[Zn(trz)(H_2_betc)_0.5_]·DMF	^[^ ^146^ ^]^
Pb^2+^	MOF‐5, UiO‐66, MIL‐101(Cr), MIL‐53(Fe), ZIF‐8, MOF‐5, IRMOF‐3	^[^ ^147^ ^]^
Pb^2+^	DUT‐67	^[^ ^148^ ^]^
Cs^+^, Sr^2+^	MIL‐101‐SO_3_H	^[^ ^149^ ^]^
Ba^2+^	MIL‐101‐SO_3_H, MOF‐808‐SO_4_	^[^ ^150^ ^]^
Cu^2+^	[(Zn_3_L_3_(H_2_O)_6_][(Na)(NO_3_)]	^[^ ^151^ ^]^
UO_2_^2+^, UO_2_(OH)^+^, UO_2_(OH)^2+^	HKUST‐1	^[^ ^152^ ^]^
UO_2_^2+^	UiO‐68‐NH_2_, UiO‐68‐P(O)(OEt)_2_, UiO‐68‐P(O)(OH)_2_	^[^ ^153^ ^]^
UO_2_^2+^	MOF‐76	^[^ ^154^ ^]^
UO_2_^2+^	Amine‐derived UiO‐66	^[^ ^155^ ^]^
UO_2_^2+^	Zn‐MOF	^[^ ^156^ ^]^
UO_2_^2+^	MISS‐PAF‐1	^[^ ^157^ ^]^
Cs^+^	[(CH_3_)_2_NH_2_]_4_[(UO_2_)_4_(TBAPy)_3_]·18DMF·17H_2_O, [(CH_3_)_2_NH_2_]_4_[(UO_2_)_4_(TBAPy)_3_]·22DMF·37H_2_O	^[^ ^158^ ^]^
As^5+^, ethylene blue	MFC‐N‐100	^[^ ^159^ ^]^
Cd^2+^	CTF‐1	^[^ ^160^ ^]^
As^3+^	MIL‐100(Fe)	^[^ ^161^ ^]^
Pb^2+^	Fe_3_O_4_/MIL‐96(Al)	^[^ ^162^ ^]^
Co^2+^, Cd^2+^, Cu^2+^, Cr^3+^, Fe^2+^, and Pb^2+^	TMU‐6,TMU‐21, TMU‐23, TMU‐24	^[^ ^163^ ^]^
SO_4_^2−^	Urea‐functionalized MOF	^[^ ^164^ ^]^
F^−^	MIL‐53(Al, Fe, Cr), MIL‐68, CAU‐1, CAU‐6, UiO‐66(Hf, Zr), ZIF‐7, ‐8, ‐9	^[^ ^165^ ^]^
F^−^	MIL‐96	^[^ ^166^ ^]^
I^−^	MOF@cellulose aerogels	^[^ ^167^ ^]^
Cr_2_O_7_^2−^	FIR‐53, FIR‐54	^[^ ^168^ ^]^
Cr_2_O_7_^2−^	ZJU‐101	^[^ ^169^ ^]^
Cr_2_O_7_^2− ^, MnO_4_^−^	Cationic Ni‐MOF	^[^ ^170^ ^]^
HCrO_4_^− ^, CrO_4_^2−^	TMU‐30	^[^ ^171^ ^]^
MnO_4_^−^, ReO_4_^−^	SLUG‐21, SLUG‐22	^[^ ^172^ ^]^
H_2_ASO_4_^−^, HASO_4_^2−^	UiO‐66	^[^ ^173^ ^]^
ASO_4_^3−^, ASO_3_^3−^	UiO‐66 analogues	^[^ ^174^ ^]^
ASO_4_^3−^	NH_2_‐MIL‐88(Fe)	^[^ ^175^ ^]^
ASO_4_^3−^	ZIF‐8/sodium alginate	^[^ ^176^ ^]^
SeO_4_^2−^, SeO_3_^2−^	NU‐1000, UiO‐66, UiO‐67	^[^ ^177^ ^]^
Cr_2_O_7_^2−^	MOF+	^[^ ^178^ ^]^
NaCl	UiO‐66 membrane	^[^ ^179^ ^]^
NaCl	MIL‐53/PVA membranes	^[^ ^180^ ^]^
Benzene	MIL‐101	^[^ ^181^ ^]^
Methyl orange, methylene blue	MIL‐53, MIL‐101, MOF‐235	^[^ ^182^, ^183^ ^]^
HAsO_4_^2−^, methylene blue, rhodamine B, coomassie brilliant blue G‐250	MIL‐100(Fe), MIL‐100(Al)	^[^ ^184^ ^]^
Methanol, ethanol, acetone, tetrahydrofuran	Zn_2_(IDC)_4_(OH)_2_(Hprz)_2_	^[^ ^185^ ^]^
Acetonitrile	Basolite F300, Z1200, C300, A100	^[^ ^186^ ^]^
Xylenol orange	MIL‐101	^[^ ^187^ ^]^
Phenol, *p*‐cresol, sugar	MIL‐53	^[^ ^188^ ^]^
Nitrobenzene	MIL‐53	^[^ ^189^ ^]^
Malachite green	MIL‐53, MIL‐100	^[^ ^190^ ^]^
Congo red, brilliant black, direct red, etc.	MBioF	^[^ ^191^ ^]^
Polycyclic aromatic hydrocarbons	Fe‐BTC	^[^ ^192^ ^]^
Saturated hydrocarbons	PCPF‐1	^[^ ^193^ ^]^
MnO_4_^−,^ dyes, methyl blue, methyl orange	3D‐ionic COFs	^[^ ^194^ ^]^
Furosemide, sulfasalazine	HKUST‐1, MIL‐100	^[^ ^195^ ^]^
Naproxen, clofibric acid	MIL‐100, MIL‐101	^[^ ^196^ ^]^
Saccharin, acesulfame, cyclamate	MAF‐6	^[^ ^197^ ^]^
Rhodamine B	Carbonized CoMOF	^[^ ^198^ ^]^
Atrazine	ZIF‐8, UiO‐66, and UiO‐67	^[^ ^199^ ^]^
Methylene blue	JLUE‐COP‐1 and JLUE‐COP‐2	^[^ ^200^ ^]^
Methylene blue	MIL‐100(Fe)‐KG, ZIF‐8‐KG, CuBTC‐KG	^[^ ^201^ ^]^
Methylene blue	PVBA‐UiO‐66	^[^ ^202^ ^]^
Oil (*n*‐hexane, gasoline, soybean oil, diesel, toluene)	F‐ZIF‐90@PDA@sponge	^[^ ^203^ ^]^
Dye	ZIF‐8 on PVDF hollow fibers	^[^ ^204^ ^]^
NaCl	ZIF‐8 membrane	^[^ ^205^ ^]^ (sim.)
NaCl	ZIF‐25, ‐71, ‐93, ‐96, and ‐97	^[^ ^206^ ^]^ (sim.)
NaCl	ZIF‐8, ‐93, ‐95, ‐97, and ‐100	^[^ ^207^ ^]^ (sim.)
NaCl	ZIF‐25	^[^ ^208^ ^]^ (sim.)
NaCl	ZIF‐8	^[^ ^209^ ^]^ (sim.)
Pb^2+^	Na‐*rho*‐ZMOF	^[^ ^210^ ^]^ (sim.)
Dimethyl sulfoxide	Zn_4_O(bdc)(bpz)_2_, Zn(bdc)(ted)_0.5_, ZIF‐71	^[^ ^211^ ^]^ (sim.)
Dimethyl sulfoxide	UiO‐66, UiO‐66‐F_4_, ‐(CH_3_)_2_, ‐(COOH)_2_, ‐(CF_3_)_2_, and ‐naphthyl	^[^ ^212^ ^]^ (sim.)
Aniline and phenol	MIL‐53(Al)	^[^ ^213^ ^]^ (exp./sim.)
Pd^2+^, Pt^4+^, Au^3+^	Zr‐MOFs, UiO‐66‐NH_2_	^[^ ^214^ ^]^ (exp./DFT)
NaCl	ZIF‐7, ‐8, and ‐90 on *α*‐Al_2_O_3_	^[^ ^215^ ^]^ (exp./sim.)
NaCl, MgCl_2_, FeCl_3_, Na_2_SO_4_, MgSO_4_	COF membranes	^[^ ^216^ ^]^ (exp./sim.)

**Figure 17:**
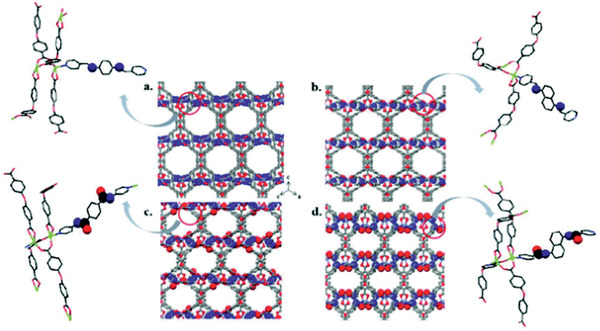
Pores with imine and amide groups in a) TMU‐6, b) TMU‐21, c) TMU‐23, and d) TMU‐24. Reproduced with permission.^[^ ^163^ ^]^ Copyright 2018, Royal Society of Chemistry.

By the coordination bonding of Cu^2+^ with –SH group in dithioglycol, Ke et al. functionalized HKUST‐1 and found it to possess remarkably high adsorption affinity and capacity for Hg^2+^, while the unfunctionalized counterpart showed no adsorption of Hg^2+^.^[^ ^140^ ^]^ Through a simple solvothermal method, Zhu et al. synthesized Fe‐BTC and examined the kinetics and thermodynamics of As^5+^ adsorption. Fe‐BTC was found to possess high As^5+^ adsorption capacity, about 6.5 times that of 50 nm Fe_2_O_3_ nanoparticles.^[^ ^141^ ^]^ Lin and co‐workers prepared three highly porous and stable MOFs with UiO‐68 network topology using amino‐TPDC or TPDC bridging ligands. These phosphorylurea‐derived MOFs were found to be efficient in sorbing uranyl ions from water and artificial seawater. The highest saturation capacity was up to 217 mg g^−1^, at least fourfold larger than that in amidoxime polymers. Density functional theory (DFT) calculations in both gas phase and water revealed that two phosphorylurea groups could bind favorably to one uranyl ion.^[^ ^153^ ^]^ To provide systematic insight into the effects of functional groups on adsorption of metal ions, Esrafili et al. synthesized four isoreticular Zn‐based MOFs (TMU‐6, ‐21, ‐23, and ‐24 shown in **Figure** **17**) by a mechanochemical method. Compared with imine and naphthyl groups, amide and phenyl groups in these MOFs were found to have greater adsorption of metal ions. Amide‐containing TMU‐23 showed efficient extraction and removal of metal ions (Co^2+^, Cd^2+^, Cu^2+^, Cr^3+^, Fe^2+^, and Pb^2+^). In addition, DFT calculations were performed on possible coordination between ion and functional group in each MOF.^[^ ^163^ ^]^

**Figure 18:**
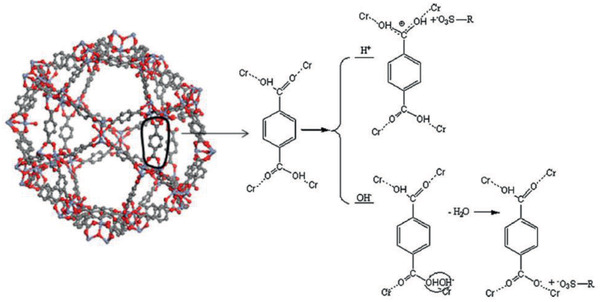
Adsorption of xylenol organe (represented by R‐SO_3_^−^) in MIL‐101. Reproduced with permission.^[^ ^187^ ^]^ Copyright 2012, Elsevier.

Under microwave irradiation, Jhung et al. synthesized MIL‐101(Cr) and examined benzene adsorption from aqueous solutions. Compared with activated carbon, MIL‐101 was observed to adsorb a higher amount of benzene. Additionally, the rate of benzene adsorption was faster due to the giant pores in MIL‐101.^[^ ^181^ ^]^ They further reported the removal of harmful dyes, anionic methyl orange (MO) and cationic methylene blue (MB) from aqueous solutions using MIL‐53, MIL‐101, and MOF‐235. The removal was revealed to be a spontaneous endothermic process and driven by entropy effect rather than enthalpy change.^[^ ^182^, ^183^ ^]^ Chen et al. reported kinetic and thermodynamic studies on the adsorption of xylenol orange (XO) into MIL‐101 from wastewater. A pseudo second‐order kinetic model was found to well describe experimental data. Thermodynamic parameters including free energy, enthalpy and entropy of adsorption were obtained and all were in favor of XO adsorption. Interestingly, the adsorbed amount was found to decrease with increasing pH value of XO solution and approach zero at pH = 12. As illustrated in **Figure** **18**, the adsorption mechanism was attributed to charge interaction between XO and MIL‐101. Compared with active carbon and MCM‐41, MIL‐101 was shown better adsorption capacity over a wider concentration range, suggesting a great prospect for dye removal.^[^ ^187^ ^]^

Hybrid gels in a capillary using Fe‐BTC were fabricated by Hu et al. and coupled with HPLC for the online enrichment of trace polycyclic aromatics from water and of amphetamines drugs from urine. As attributed to *π*–*π* interactions between the Fe‐BTC framework and aromatic rings of analytes, excellent enrichment performance was observed.^[^ ^192^ ^]^ Via homo‐coupling reaction of a custom‐designed ligand, Ma and co‐workers synthesized a highly porous covalent porphyrin framework (PCPF‐1) with a strong hydrophobicity. PCPF‐1 was shown high adsorptive capacities for both vapor and liquid saturated C_5_‐C_8_ hydrocarbons as well as liquid gasoline towards the cleanup of oil spill in water.^[^ ^193^ ^]^

Pharmaceutical compounds have turned out to be an emerging class of environmental contaminants in water, thus are required to be removed. Cychosz and Matzger examined water stability of seven microporous MOFs using power X‐ray diffraction, then tested HKUST‐1 and MIL‐100 for the adsorption of furosemide and sulfasalazine. Large adsorption capacities were obtained in MIL‐100, implying the potential application of MIL‐100 for water purification.^[^ ^195^ ^]^ The adsorptive removal of two typical pharmaceuticals and personal care products (naproxen and clofibric acid) was also investigated by Jhung and co‐workers in MIL‐100 and MIL‐101, as well as in activated carbon. The adsorption rate and capacity were found to decrease in the order of MIL‐101 > MIL‐100 > activated carbon, largely depending on the pore size and volume of adsorbent.^[^ ^196^ ^]^

**Figure 19:**
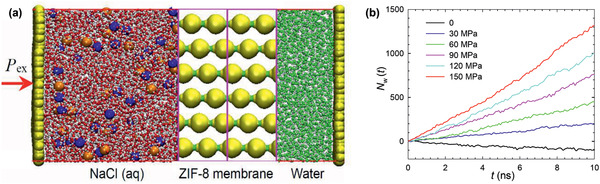
a) Water desalination through a ZIF‐8 membrane. Two chambers (aqueous solution with 0.5 M NaCl on the left and pure water bath on the right) were separated by a ZIF‐8 membrane. ZIF‐8, yellow; Na^+^, blue; Cl^−^, gray; H of H_2_O, white; O of H_2_O, magenta (left chamber), and green (right chamber). b) Number of net transferred water molecules from NaCl solution to pure water bath. Reproduced with permission.^[^ ^205^ ^]^ Copyright 2011, American Institute of Physics.

It is worthwhile to note that Jiang's group reported several simulation studies on water treatment by MOFs, including water desalination, removal of toxic Pb^2+^ and dimethyl sulfoxide (DMSO) from water. Specifically, ZIF‐8 membrane was demonstrated to act as a reverse osmosis (RO) membrane to seawater desalination, as shown in **Figure** **19**. Under a sufficiently large external pressure, water was observed to permeate through the ZIF‐8 membrane, whereas Na^+^ and Cl^−^ ions could not due to the sieving of small apertures in ZIF‐8. Water flux was found to scale linearly with the external pressure. Because of the surface interaction and geometrical confinement, water in the membrane was predicted to experience less hydrogen‐bonding and longer life time compared with bulk water.^[^ ^205^ ^]^ Subsequently, water desalination was simulated through five ZIF membranes (ZIF‐25, ‐71, ‐93, ‐96, and ‐97) with identical topology but different functional groups. With larger apertures, ZIF‐25, ‐71, and ‐96 were shown to possess a much higher water flux than ZIF‐93 and ‐97. However, the polarity of functional group rather than aperture size was revealed to determine water flux through ZIF‐25, ‐71, and ‐96. Among the three, ZIF‐25 with hydrophobic –CH_3_ groups showed the highest flux despite the smallest aperture size. Water molecules were observed to go through fast flushing motion in ZIF‐25, but frequent jumping in ZIF‐96 and particularly in ZIF‐97.^[^ ^206^ ^]^ Furthermore, seawater pervaporation in series of ZIFs (ZIF‐8, ‐93, ‐95, ‐97, and ‐100) was simulated. ZIF‐100 with the largest aperture was predicted to possess the highest water permeability of 5 × 10^−4^ kg m m^−2^ h^−1^ bar^−1^, which is higher than commercial RO membranes, zeolite and graphene oxide membranes. For ZIF‐8, ‐93, ‐95, and ‐97 with similar aperture size, water flux was revealed to depend on framework hydrophobicity. A to‐and‐fro motion was seen for water in ZIF‐100, such information on dynamic properties of water is useful to design new membranes for water desalination.^[^ ^207^ ^]^

**Figure 20:**
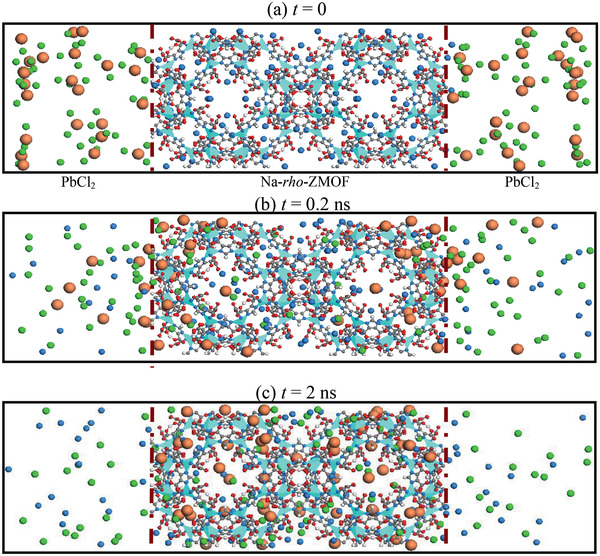
Removal of Pb^2+^ by ion exchange in Na‐*rho*‐ZMOF at 0, 0.2, and 2 ns, respectively. Pb^2+^: orange; Cl^−^: green; Na^+^: blue. Reproduced with permission.^[^ ^210^ ^]^ Copyright 2012, American Chemical Society.

To remove toxic ions from water, Nalaparaju and Jiang simulated the exchange of Pb^2+^ ions in aqueous solution with Na^+^ ions in Na‐*rho*‐ZMOF. **Figure** **20** shows the ion‐exchange process at different durations. Initially, Pb^2+^ and Cl^−^ ions were in the solution and Na^+^ ions in *rho*‐ZMOF. Once starting simulation, Pb^2+^ ions were observed to rapidly move into the framework, while Na^+^ ions moving out. At 0.2 ns, a large number of Pb^2+^ ions moved into *rho*‐ZMOF, particularly near the solution/*rho*‐ZMOF interface. Meanwhile, Na^+^ ions moved out and stayed in the solution. Once exchanged, Pb^2+^ ions were found to prefer staying in the framework without moving back to solution. At 2 ns, all the Pb^2+^ ions were exchanged and resided in the framework. By umbrella sampling, the potentials of mean force (PMFs) were estimated for ions moving from the solution into *rho*‐ZMOF. The PMF for Pb^2+^ was predicted about −10 *k*_B_ *T* and more favorable than −5 *k*_B_ *T* for Na^+^. From the residence‐time distributions and mean‐squared displacements, all the exchanged Pb^2+^ ions were shown to constantly stay in *rho*‐ZMOF without exchanging with other ions in the solution. The exchanged Pb^2+^ ions in *rho*‐ZMOF were identified to locate at the 8‐, 6‐, and 4‐member rings with different dynamics.^[^ ^210^ ^]^ This simulation study provides microscopic insight into ion exchange between aqueous solution and *rho*‐ZMOF. However, it should be noted that the solvent (water) was represented by a dielectric continuum. While offering several advantages, the implicit solvent approach lacks atomistic resolution particularly in dynamics. To more precisely describe the dynamics, solvent molecules should be explicitly taken into account, but simulation will be substantially longer.

Nalaparaju and Jiang also conducted a simulation study to examine DMSO recovery from aqueous solution by Zn_4_O(bdc)(bpz)_2_, Zn(bdc)(ted)_0.5_, and ZIF‐71. Due to the hydrophobic nature, the three MOFs showed highly selective adsorption of DMSO from DMSO/H_2_O mixtures. The adsorption selectivity of DMSO over H_2_O was increased initially with increasing the composition of DMSO (*X*_DMSO_), then decreased within the range of *X*_DMSO_ considered in the study. The initial increase was attributed to the favorable interaction of DMSO with multiple adsorption sites in the framework. However, the interaction could weaken when most adsorption sites were occupied and furthermore smaller H_2_O molecules were more easily fill into the framework; consequently, a decrease in selectivity was seen at high *X*_DMSO_. The highest selectivity was predicted up to 1700 in ZIF‐71; nonetheless, the recovery capacity of DMSO in ZIF‐71 was substantially smaller than in Zn_4_O(bdc)(bpz)_2_ and Zn(bdc)(ted)_0.5_. Therefore, Zn_4_O(bdc)(bpz)_2_ and Zn(bdc)(ted)_0.5_ might be practically better among the three MOFs for DMSO recovery.^[^ ^211^ ^]^

**Figure 21:**
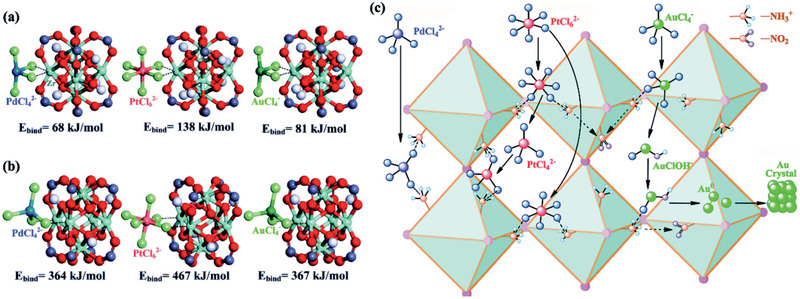
Binding energies of anions onto a) one Zr site and b) two Zr sites. c) Binding modes and reduction pathways of anions in UiO‐66‐NH_2_. Reproduced with permission.^[^ ^214^ ^]^ Copyright 2017, Royal Society of Chemistry.

By combining experiment and simulation, the adsorption of aniline/phenol from aqueous solutions by MIL‐53(Al) was investigated by Zhong and co‐workers. Besides framework flexibility, coadsorbed water molecules were also found to affect the adsorption of both aniline and phenol. Interestingly, more water molecules were trapped in MIL‐53(Al) accompanying with phenol adsorption (0.64 water molecules per unit cell versus 0.11 in the case of aniline adsorption). This was attributed to stronger hydrogen bonding of phenol with water than of aniline. This study suggests such a flexible material might be useful for separating aniline/phenol from their mixed aqueous solutions.^[^ ^213^ ^]^ Yun and co‐workers explored the adsorption of PdCl_4_^2−^, PtCl_6_^2−^, and AuCl_4_^−^ from strongly acidic solutions by UiO‐66 and UiO‐66‐NH_2_. Experimentally, both MOFs showed rapid uptake rate and high adsorption capacity. As shown in **Figure** **21**, their DFT calculations indicated the anions binding onto two Zr sites with energies higher than those binding onto one Zr site. The inner‐sphere complexation between anions and incompletely coordinated Zr sites was revealed to be the key mechanism contributing to the adsorption. In UiO‐66‐NH_2_, additional electrostatic attraction between the protonated amine group (–NH_3_^+^) and anions was observed along with the partial reduction of bound anions.^[^ ^214^ ^]^ Through post‐modification, Huang and co‐workers constricted the pore aperture of a covalent organic framework (COF) membrane, IISERP‐COOH‐COF1. With superior stability, the membrane exhibited high water permeance above 0.5 L m^−2^ h^−1^ bar^−1^ and superior ion rejection for various salts (82.9% NaCl, 90.6% MgCl_2_, 99.6% FeCl_3_, 96.3% Na_2_SO_4_, and 97.2% MgSO_4_). The experimental observation was supported by molecular simulation.^[^ ^216^ ^]^

The development of water stable MOFs, particularly MIL, UiO and ZIF families, has provided plenty of opportunities to use MOFs for water treatment. As discussed above, with diversified pores, topologies and structures, MOFs can adsorb a wide variety of molecules ranging from small ions to large organics from water or aqueous solutions. For cation removal, MOFs possess the intrinsic mechanisms from both organic and inorganic counterparts, such as chelation by organic functional groups and ion exchange with framework or nonframework ions. MOFs can also be used for selective anion removal by ion exchange, which is not easily achievable in many other porous materials.^[^ ^164^ ^]^ For organics removal, pore volume, surface area, *π*–*π* and electrostatic interactions are the main governing factors. Compared with zeolites and activated carbons, MOFs exhibit higher capacity and efficiency for the removal of large organics. Furthermore, MOFs have been tested for membrane‐based water treatment processes such as ultrafiltration, forward osmosis, reverse osmosis and nanofiltration. Computational studies are instrumental to provide mechanistic insights into the fundamental mechanisms of adsorption, ion exchange and membrane separation for water treatment.

### 3.2 Solvent Recovery

In the chemical and pharmaceutical industries, organic solvents are widely used as raw materials, reaction media and cleaning agents. A large amount of solvents after use are simply disposed by incineration. From environmental and economic aspects, it is beneficial to recover solvents. Adsorption is not well‐suited for large‐scale solvent recovery due to high energy consumption, low efficiency and difficulty in recycling. Instead, membrane separation is more effective with low energy requirement, high selectivity, flexibility in design and environmental friendliness. Organic solvent nanofiltration (OSN) is a promising membrane‐based, energy efficient technology for solvent recovery. Most of the current OSN membranes are polymer based. To attain superior‐performance OSN, a narrow pore size distribution is important for the discrimination of molecules of similar size, which however is difficult to be produced in polymer membranes. In this aspect, microporous crystalline materials are of immense potential to be developed into OSN membranes. Recently, different types of MOF‐based OSN membranes have been produced, namely, mixed‐matrix membranes (MMMs), thin‐film nanocomposite (TFN) membranes, and thin‐film composite (TFC) membranes. Examples of these membrane used for solvent recovery are listed in **Table** **4**.

**Table 4:** Solvent Recovery

Solution	MOF	Ref.
**MMM**		
Rose bengal in isopropanol	Cu_3_(BTC)_2_, MIL‐47, MIL‐53, ZIF‐8/PDMS	^[^ ^217^ ^]^
Polystyrene oligomer, methyl styrene dimer in acetone	ISG HKUST‐1/PI P84	^[^ ^218^, ^219^ ^]^
Brilliant blue G in ethanol	MIL‐53(Al)/PMIA	^[^ ^220^ ^]^
Reactive red 120, methyl blue, reactive orange 16, methyl red in methanol	Cu‐BTC/PPSU	^[^ ^221^ ^]^
Reactive orange 16 in methanol	Cu‐BTC/PPSU	^[^ ^222^ ^]^
Rose bengal, methylene red in methanol	ZIF‐8@resin microspheres/PPSU	^[^ ^223^ ^]^
Congo red in ethanol and IPA	Carbonized ZIF‐8/PI	^[^ ^224^ ^]^
**TFN**		
Polystyrene oligomer in methanol and tetrahydrofuran	NH_2_‐MIL‐53(Al), MIL‐53(Al), ZIF‐8, MIL‐101(Cr)/P84	^[^ ^225^ ^]^
Acridine orange, sunset yellow, and Rose bengal in methanol	MIL‐101(Cr), MIL‐68(Al), ZIF‐11/P84	^[^ ^226^ ^]^
Sunset yellow, acridine orange in methanol	MIL‐101(Cr) and ZIF‐11/P84	^[^ ^227^ ^]^
Sunset yellow in methanol	ZIF‐8 and ZIF‐67/P84	^[^ ^228^ ^]^
Methyl blue dye in methanol	ZIF‐8@GO composite/PEI	^[^ ^229^ ^]^
Rose bengal in various solvents	UiO‐66/PA	^[^ ^230^ ^]^
Tetracyline in methanol	DA‐UiO‐66‐NH_2_/PI	^[^ ^231^ ^]^
Rose bengal in ethanol	PAF‐1/PTMSP	^[^ ^232^ ^]^
Erythromycin and dyes in methanol, tetrahydrofuran, dimethyl formamide and butyl acetate	*β*‐CD@ZIF‐8/PA	^[^ ^233^ ^]^
Sunset yellow in methanol	ZIF‐8, ZIF‐93 and UiO‐66/P84	^[^ ^234^ ^]^
Rose bengal, sunset yellow in methanol	LS‐MIL‐101(Cr)/P84	^[^ ^235^ ^]^
Rhodamine B in ethanol and Rose bengal in DMF	SNW‐1 COF/PI	^[^ ^236^ ^]^
**TFC**		
Rose bengal in ethanol or IPA	ZIF‐8/PES	^[^ ^237^ ^]^
Polystyrene oligomer, methyl styrene dimer in acetone	HKUST‐1/P84	^[^ ^238^ ^]^
Brilliant blue R dye in IPA	Zn(BDC)/PAN	^[^ ^239^ ^]^
Rose bengal dye in methanol and isopropanol	MIL‐53(Al) and NH_2_‐MIL‐53(Al)	^[^ ^240^ ^]^
Dyes in acetonitrile, acetone, methanol, ethanol, isopropanol and dimethylformamide	CON films	^[^ ^241^ ^]^
Paracetamol in methanol, ethanol, acetonitrile, acetone and *n*‐hexane	ZIF‐25, ‐71, ‐96	^[^ ^242^ ^]^ (sim.)
FDA, PRM, MSD and NRD in acetonitrile, acetone, methanol, ethanol, IPA, MEK, and *n*‐hexane	COFs	^[^ ^243^ ^]^ (sim.)

**Figure 22:**
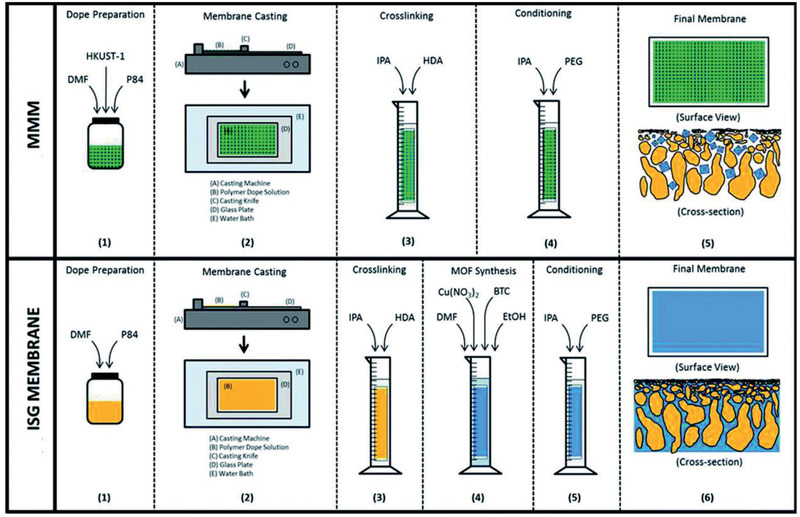
Schematic in situ growth of MMMs. Reproduced with permission.^[^ ^218^ ^]^ Copyright 2014, Royal Society of Chemistry.

Vankelecom and co‐workers reported the earliest MMMs by filling ZIF‐8, MIL‐47, MIL‐53, and HKUST‐1 into polydimethylsiloxane for the separation of Rose bengal from iso‐propanol, and found higher permeance but lower retention compared with unfilled membranes. However, the use of trimethylsilyl surface modification could improve the adhesion of MOF fillers with polymer and thus enhancing retention. The superior performance of the MMM was attributed to the reduced polymer swelling and size exclusion of the fillers.^[^ ^217^ ^]^ To generate a perfect interaction between polymer and MOF and reduce non‐selective voids in MMMs, Campbell et al. proposed a novel membrane fabrication method utilizing in situ growth (ISG) of MOF crystals in preformed polymer membranes, as shown in **Figure** **22**. The OSN performance of HKUST‐1/P84 membrane prepared by the ISG was compared with MMMs formed without using the ISG and controlled ultrafiltration membrane. It was found that chemically modified ISG membranes outperform non‐modified ISG membranes in both solute retention and solvent permeance.^[^ ^218^ ^]^

From non‐solvent induced phase separation, Zhu et al. produced MMMs using MIL‐53 and poly(m‐phenyleneisophthalamide). They observed a significant increase in ethanol permeance while a slight reduction in rejection of brilliant blue G upon comparing with pristine polymer membrane; nevertheless, the rejection was up to 4.5 times of that in a commercial membrane.^[^ ^220^ ^]^ By growing ZIF‐8 core–shell nanoparticles on the outer surface of macroporous resin microspheres (RMs) in polyphenylsulfone (PPSU), Dai et al. prepared PPSU/ZIF‐8@RMs membranes. These membranes exhibited higher rejection of methyl red from methanol and better stability compared with PPSU/ZIF‐8 and PPSU/RMs membranes. This was attributed to the combination of high affinity and microporous structure of ZIF‐8 nanoparticles, thus resulting in reduced defects between ZIF‐8@RMs and PPSU.^[^ ^223^ ^]^

In the preparation of polyimide (PI)‐based MMMs, Wang et al. proposed a strategy to overcome structure degradation of ZIF‐8 due to protonation effect in an acid environment of polyamide acid (PAA) solution. The coordinating linkers of ZIF‐8 were transferred into carbon skeleton through direct carbonization. It was observed that the unique framework of carbonized ZIF‐8 (CZIF‐8) showed superior stability in the MMM preparation process. Furthermore, CZIF‐8 provided selective flow paths for solvents due to improved porosity and enhanced adsorption capacity. Particularly, 10 wt% CZIF‐8/PI MMM showed a high rejection > 90% for Congo red and remarkable permeances for water, ethanol and isopropanol. This study suggests the strategy can effectively enhance ZIF‐8 stability in preparation of ZIF‐8 based MMMs in an acid environment.^[^ ^224^ ^]^

**Figure 23:**
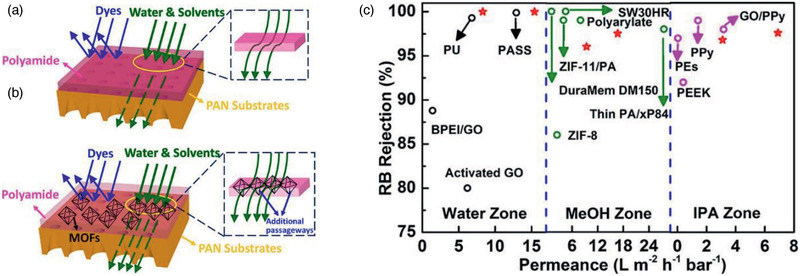
a) Conventional PA membrane. b) UiO‐66/PA TFN membrane. c) Comparison of separation performance with other nanofiltration membranes. Reproduced with permission.^[^ ^230^ ^]^ Copyright 2017, American Chemical Society.

Recently, TFN membranes have received strong interest for OSN. Notably, Livingston and co‐workers produced several TFN membranes containing ZIF‐8, MIL‐53, MIL‐53‐NH_2_, and MIL‐101 nanoparticles in a PA thin‐film layer on top of PI support, then evaluated their OSN performance on the basis of methanol and tetrahydrofuran permeances and the rejection of styrene oligomers. Increased solvent permeances were observed in the TFN membranes and the increase was in good correlation with the pore size and porosity of the MOFs.^[^ ^225^ ^]^ Using a dip‐coating method, Sarango et al. controlled the deposition of ZIF‐8 and ZIF‐67 particles on top of a polyimide P84 asymmetric support prior to the interfacial polymerization of polyamide, and tested for OSN in a methanol solution with sunset yellow. A rejection of 90% and a high methanol permeance of 8.7 L m^−2^ h^−1^ bar^−1^ were achieved in ZIF‐8 containing TFN membrane. This study highlights the advantages of using dip‐coating methodology for controlling the location of MOF nanoparticles in preparing TFN membranes.^[^ ^228^ ^]^ Cheng et al. fabricated ultrathin TFN membranes by incorporating hydrostable UiO‐66 into PA. The effects of functionalized ligands (UiO‐66‐(CH_3_)_2_, UiO‐66‐NH_2_) and postsynthetic functionalization (UiO‐66(Ti)) on tailoring the nonbonded, intimate interactions between UiO‐66 and PA chains were investigated to ensure uniform UiO‐66 dispersion within the PA selective layer. Due to the inherent porous nature of UiO‐66, additional molecular transport passageways were provided in PA membranes, as depicted in **Figure** **23**, which would drastically enhance solvent permeation without compromising separation performance. Upon comparison, the UiO‐66/PA TFN membranes were found to outperform many nanofiltration membranes.^[^ ^230^ ^]^ Zhong and co‐workers modified UiO‐66‐NH_2_ nanoparticles with long alkyl chains and used them to prepare TFN membranes on polyamide; they found significant enhancement in methanol permeance without comprising tetracycline rejection.^[^ ^231^ ^]^

**Figure 24:**
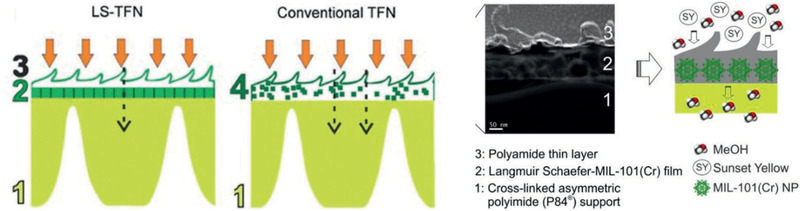
(1) Cross‐linked asymmetric polyimide (P84) support (2) Langmuir–Schaefer (LS)‐MIL‐101(Cr) monolayer (3) polyamide selective layer and (4) polyamide layer with MIL‐101(Cr) nanoparticles (NPs) inside. Reproduced with permission.^[^ ^235^ ^]^ Copyright 2018, American Chemical Society.

Xu et al. developed TFN membranes by doping *β*‐CD@ZIF‐8 nanoparticles for the nanofiltration of antibiotic erythromycin (EM) in methanol and butyl acetate. The membranes encompassed two PA layers on a polyimide support. One was a poly(*m*‐phenylene isophthalamide) (PMIA) support layer and the other being a PA selective layer. The *β*‐CD@ZIF‐8 nanoparticles were doped in both selective PA layer and PMIA support layer to provide an efficient way for EM concentration and purification. Several adjustable parameters were identified such as the concentration of PMIA in support, the contents of aqueous and organic phases, and the dosage of *β*‐CD@ZIF‐8 nanoparticles to improve membrane performance.^[^ ^233^ ^]^ Navarro et al. reported a novel procedure for positioning a monolayer of hydrophilic MIL‐101(Cr) nanoparticles in TFN membranes. As described in **Figure** **24**, it was achieved by transferring a Langmuir–Schaefer (LS) film of MIL‐101(Cr) between a top polyamide thin layer and a bottom cross‐linked asymmetric polyimide (P84) support. Such a membrane preparation method required the smallest amount of MOF and would assist in the formation of defect‐free ultrathin MOF films. Unlike conventional TFN membranes with agglomeration of MOF nanoparticles and formation of unselective defects, the LS‐TFN membranes were characterized by a homogeneous and continuous MOF coating. Excellent methanol permeance was observed when filtering sunset yellow and Rose bengal with rejection higher than 90%, as solvent permeation through the polyamide layer was greatly enhanced by the LS‐MIL‐101(Cr) monolayer.^[^ ^235^ ^]^

**Figure 25:**
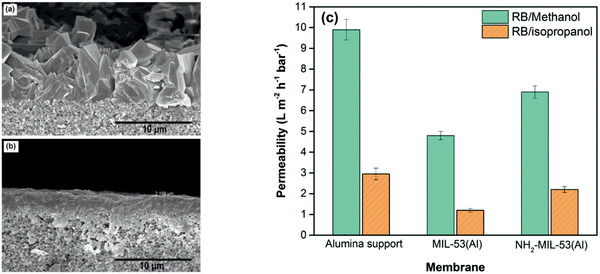
Cross‐sectional FE‐SEM images of a) MIL‐53(Al) and b) NH_2_‐MIL‐53(Al) membranes on an *α*‐alumina support. c) Methanol and isopropanol permeability. Reproduced with permission.^[^ ^240^ ^]^ Copyright 2019, Royal Society of Chemistry.

Compared with MMMs and TFN membranes, TFC membranes with continuous MOF layer are less investigated. By a simple interfacial synthesis method, Li et al. prepared ZIF‐8 membranes on a porous polyethersulfone support and confirmed membrane integrity using SEM, TEM, and ATR‐FTIR. The structure of ZIF‐8 crystals formed by this method was found to be the same as that conventionally prepared in DMF but with an aggregated morphology rather than uniform crystals. These TFC membranes exhibited high performance in the removal of Rose bengal from water, ethanol and isopropanol with permeances in the order of ethanol–water > isopropanol. Due to the flexible structure and hydrophobic pore of ZIF‐8, ethanol and water were observed to have close permeance.^[^ ^237^ ^]^ Chung and co‐workers used a green layer‐by‐layer method to produce polyacrylonitrile supported zinc benzene‐1,4‐dicarboxylicacid MOF membranes. In contrast to most synthesis methods for MOFs requiring the use of organic solvents and elevated temperatures, only water was used as a solvent at room temperature by utilizing terephthalate salts as organic linker. Subsequently, cross‐linking and hydrolyzing were also conducted in water to prepare polymer layer and the layer‐by‐layer growth of MOF selective layer. The produced TFC membranes were evaluated for OSN and compared with other membranes.^[^ ^239^ ^]^ Moreover, MIL‐53(Al) and NH_2_‐MIL‐53(Al) TFC membranes on an *α*‐alumina support were prepared by Amirilargani et al. for the removal of Rose bengal from organic solvents. The TFC membranes were found to possess lower methanol and isopropanol permeability compared with unmodified *α*‐alumina membranes, as shown in **Figure** **25**; however, the NH_2_‐MIL‐53(Al) membrane had a relatively higher permeability than MIL‐53(Al). Almost no RB was adsorbed from methanol in the *α*‐alumina membrane and the amount adsorbed in NH_2_‐MIL‐53(Al) membrane was nearly 70% higher than that in MIL‐53(Al) membrane. Based on methanol permeability in the state‐of‐the‐art ceramic OSN membranes ranging from 3.9 to 6.1 L m^2−^ h^1−^ bar^1−^, these TFC membranes were considered to be efficient in removing Rose bengal along with high solvent permeability.^[^ ^240^ ^]^

**Figure 26:**
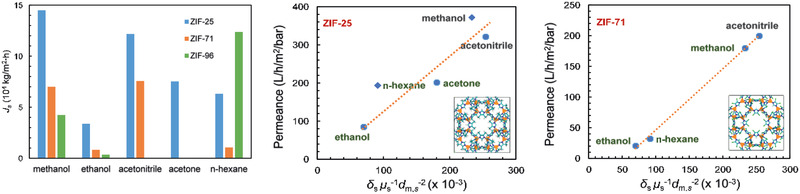
Solvent fluxes through ZIF‐25, ZIF‐71, and ZIF‐96. Correlations between permeances and a combination of solvent properties in ZIF‐25 and ZIF‐71. Reproduced with permission.^[^ ^242^ ^]^ Copyright 2018, American Chemical Society.

The above experimental studies demonstrate increasing interest in MOF‐based membranes for OSN, but the development is still at its early stage. Heretofore, to the best of our knowledge, there are only two related simulation studies. Jiang and co‐workers simulated OSN process through three ZIFs (ZIF‐5, ZIF‐71 and ZIF‐96) with identical topology and similar pore size but different functional groups. As shown in **Figure** **26**, fluxes were predicted to decrease for polar solvents (methanol ethanol, acetonitrile and acetone) as ZIF‐25 > ZIF‐71 > ZIF‐96, whereas for nonpolar solvent (*n*‐hexane) as ZIF‐96 > ZIF‐25 > ZIF‐71. The reasons were attributed to weak interaction of polar solvent with hydrophobic ZIF‐25, and weak interaction between nonpolar solvent with hydrophilic ZIF‐96. Moreover, good correlations were found between solvent permeances and a combination of solvent properties including solubility parameter, viscosity and diameter. The ZIF membranes showed perfect rejection for a model solute (paracetamol) in all the five solvents. This simulation study highlights the important role of pore chemistry in solvent permeation, in addition to pore size, and it suggests the potential use of ZIFs for OSN.^[^ ^242^ ^]^

**Figure 27:**
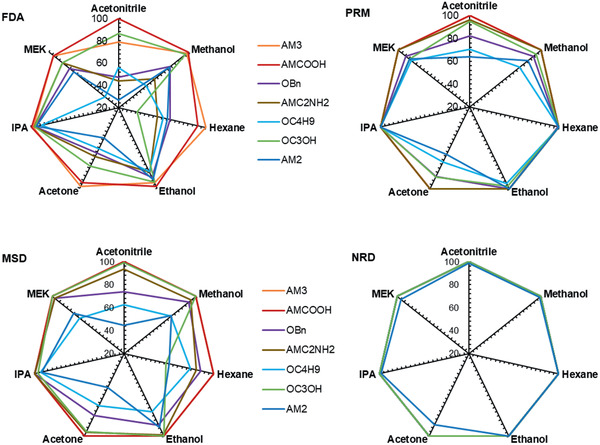
Solute rejection in seven solvents by TpPa‐X membranes (TpPa‐AM_3_, –AMCOOH, –OBn, –AMC_2_NH_2_, –OC_4_H_9_, –OC_3_OH). Reproduced with permission.^[^ ^243^ ^]^ Copyright 2019, American Chemical Society.

Furthermore, they computationally designed seven ultrathin 2D TpPa‐X (Tp = 1,3,5‐triformylphloroglucinol, Pa = *p*‐phenylenediamine) COF membranes with different functional groups and aperture sizes for OSN. In total, 245 sets of simulations were performed for seven solvents (acetonitrile, acetone, methanol, ethanol, isopropanol, methyl ethyl ketone and *n*‐hexane) and four solutes (2,5‐furandiamine, paracetamol, *α*‐methylstyrene dimer, and nile red). Solvent fluxes through the COF membranes were revealed to be governed by the aperture size and membrane functionality, as well as solvent property. For membranes with comparable aperture size, the hydrophobic one exhibited higher fluxes than the hydrophilic counterpart for all the solvents except *n*‐hexane. To elucidate this trend, solvent structures near the membranes were analyzed and the potentials of mean force for solvent permeation were evaluated. The solvent permeances through hydrophobic and hydrophilic membranes were correlated respectively with two different combinations of solvent properties. As shown in **Figure** **27**, solute rejection was found to depend on a complex interplay among solute size and polarity, solvent viscosity, solute‐solvent interaction, aperture size and membrane functionality. In the presence of solute, solvent permeances were observed to reduce by ≈ 10%. This comprehensive computational study furnishes quantitative insight into solvent permeation and solute rejection in the COF membranes and would assist the development of new membranes for high‐performance OSN.^[^ ^243^ ^]^

The interest of implementing MOFs for solvent recovery is rapidly growing, attributed to advance in membrane design and development of new solvent‐resistant MOFs. However, the current studies on MOF‐based membranes for solvent recovery are still limited. Only a few specific MOFs have been explored because of the challenge in preparing uniformly dispersed MOF membranes. Most of the current studies are focused on two main areas. One is the selection of MOFs with suitable textural features and chemical functionalities; the other is the preparation methods of MOF membranes. In OSN application, MOFs are used primarily to overcome the trade‐off between selectivity and permeability of polymer membranes, thus enhancing separation performance. With appropriate design, additional adsorption sites and transport pathways provided by MOFs can enhance solvent permeation and solute rejection.

### 3.3 Chemical Sensing

Identifying the presence of harmful and toxic compounds from drinking water, food products, fuels and valuable chemical substances is crucial to ensure purity and safety. For a material to be suitable for chemical sensing, reliable detection and differentiation among target species are imperative. Introducing ordered pores to a sensing material can provide a large surface area and selectively detect target species even at a very low concentration.

Compare with other materials, MOFs are excellent in tuning above characteristics, additionally they offer several transducing methods (e.g., luminescent, electrochemical and mechanical). MOF‐based sensors have been explored for analytes in both gas and liquid phases. While solution‐based powdery sensor probes are commonly employed for normal samples, nanoscaled MOFs (NanoMOFs) or thin film‐based probes are more suitable for biomedical sensing. Particularly, film‐based sensors have distinct advantages of tunable shape and size, good stability, recycling, and portability for non‐invasive real‐time detection.

Luminescent MOFs are the most extensively investigated because of their direct usability as chemical sensors without integrating to external transducing mechanism. These MOFs are able to recognize and produce visible signal that can be monitored directly. Luminescence in MOFs was initially realized two decades ago^[^ ^244^ ^]^ and the corresponding exploration on sensing application in liquid phase started in 2004.^[^ ^245^, ^246^ ^]^ By virtue of their nature, MOFs display a wide range of luminescent behavior via tailoring organic linkers, metal ions, antenna effects, and interactions with guest molecules.^[^ ^247^ ^]^ Studies on guest inclusive luminescence have paved the way for sensing applications.^[^ ^248^, ^249^, ^250^, ^251^ ^]^ The visible variations induced by guest molecules can be either the shift of emission spectra (in emitting color) or the change of fluorescence/phosphorescence intensity with turn‐on and ‐off mechanism to enhance or quench luminescence. **Table** **5** summarizes chemical sensing studies in liquid phase using MOFs. The analytes can be broadly classified as cations, anions, volatile organic compounds (VOCs), explosives and biological molecules in either aqueous or organic solutions. Representative studies that are conceptually different and demonstrated for different sensing applications are discussed below.

**Table 5:** Chemical sensing

Species	MOF	Ref.
Zn^2+^	[Ln(PDA)_3_Mn_1.5_(H_2_O)_3_]·3.25H_2_O	^[^ ^245^ ^]^
Mg^2+^	[Dy(L^1^)_3_Mn_1.5_(H_2_O)_3_]⋅3.125 H_2_O and [Dy(L^2^)_3_Mn_1.5_(H_2_O)_6_]⋅8.25 H_2_O	^[^ ^246^ ^]^
Water in organic solvents	AEMOF‐1	^[^ ^252^ ^]^
H^+^ in water (pH sensor)	PCN‐222	^[^ ^253^ ^]^
H^+^ in water (pH sensor)	UiO‐66‐NH_2_, UiO‐66‐N=N‐ind	^[^ ^254^ ^]^
Cu^2+^	Eu_2_(FMA)_2_(OX)(H_2_O)_4_·4H_2_O	^[^ ^255^ ^]^
Cu^2+^	[Cd_2_L_2_]⋅NMP⋅MeOH	^[^ ^256^ ^]^
Cu^2+^ and methylviologen in water	InPCF‐1	^[^ ^257^ ^]^
Cd^2+^	Eu/UiO‐66‐(COOH)_2_	^[^ ^258^ ^]^
Hg^2+^	Cd‐EDDA	^[^ ^259^ ^]^
Hg^2+^ both in vitro and in vivo	UiO‐68‐NCS, UiO‐68‐R6G, and UiO‐68‐R6G	^[^ ^260^ ^]^
Cd^2+^	Eu/CPM‐17‐Zn	^[^ ^261^ ^]^
Fe^3+^	Eu/MIL‐53‐COOH(Al)	^[^ ^262^ ^]^
Fe^3+^	Cu_3_(CN)_3_(MPTZ)	^[^ ^263^ ^]^
Fe^3+^	[Ln(HL)(H_2_O)_2_]*_n_*⋅2H_2_O	^[^ ^264^ ^]^
Fe^3+^	[Cd(p‐CNPhHIDC)(4,4’‐bipy)_0.5_]*_n_*, [Zn(p‐CNPhHIDC)(4,4’‐bipy)]*_n_*	^[^ ^265^ ^]^
Fe^3+^ and Fe^2+^, Cr_2_O_7_^2−^ and acetone	Eu^3+^@MIL‐124	^[^ ^266^ ^]^
Fe^3+^ and Al^3+^, TNP	([Tb(L1)_1.5_(H_2_O)	^[^ ^267^ ^]^
Fe^3+^, Hg^2+^ and Cr^3+^	FSOF (AIE)	^[^ ^268^ ^]^
Fe^3+^ in water	Cu‐MOFs	^[^ ^269^ ^]^
Fe^3+^ in water	Eu‐MOF/EDTA‐NiAl‐CLDH–M motor	^[^ ^270^ ^]^
Fe^3+^ in water	Tb–HIAAC	^[^ ^271^ ^]^
Fe^3+^ in water	Zn‐DTA and Cd‐DTA	^[^ ^272^ ^]^
Hg^2+^	RuUiO‐67	^[^ ^273^ ^]^
Al^3+^ and Lysine in HEPES buffer	Cd‐TCOOH	^[^ ^274^ ^]^
Fe^3+^ and CrO_4_^2−^/Cr_2_O_7_^2−^ in water	[La(SIP)(H_2_O)_3_]·H_2_O	^[^ ^275^ ^]^
Fe^3+^ and CrO_4_^2−^/Cr_2_O_7_^2−^ in water, pesticides in DMF	[Ag(CIP)]	^[^ ^276^ ^]^
CO_3_^2−^, SO_4_^2−^, PO_4_^2−^ and NO_3_^2−^	[Tb(Mucicate)_1.5_(H_2_O)_2_]·5H_2_O	^[^ ^277^ ^]^
Br^−^, NO_3_^−^, IO_4_^−^, BF_4_^−^, ClO_4_^−,^ and PF_6_^−^	[Zn_2_(bipy)_3_(H_2_O)_8_(ClO_4_)_2_(paba)_2_]·2(bipy)·4H_2_O and [Cd_2_(bipy)_4_(H_2_O)_6_(ClO_4_)_2_(paba)_2_]·(bipy)·5H_2_O	^[^ ^278^ ^]^
ClO_4_^−^, NO_3_^−^, NO_2_^−^, Cl^−^, and I^−^	Cd(µ_2_‐Cl)(µ_4_‐5MT)	^[^ ^279^ ^]^
F^−^, Cl^−^, and Br^−^	MOF‐76	^[^ ^280^ ^]^
Fe^3+^, Cr_2_O_7_^2−^ and isoquinoline antibiotics	[Zn(ACA)_4_]·CB[6]·[NH_2_(CH_3_)_2_]·8H_2_O	^[^ ^281^ ^]^
Fe^3+^ and CrO_4_^2−^/Cr_2_O_7_^2−^ in water	[Zn(L)(H_2_O)_2_]·H_2_O and Cd(L)(H_2_O)_2_]·4H_2_O	^[^ ^282^ ^]^
Cu^2+^ and Cr_2_O_7_^2−^ in DMF	[Zn(OBA)_2_(PTD)·2DMF·2H_2_O]*_n_*	^[^ ^283^ ^]^
Cu^2+^ and TNP in DMF and water	[Cd_2_(2‐abpt)(Hbtca)(H_2_btca)_0.5_(H_2_O)]*_n_* [Cd_3_(2‐abpt)(btca)_1.5_]·H_2_O	^[^ ^284^ ^]^
Fe^3+^ in ethanol and nitro explosives in DMF	[Zn(H_2_L)(2,2‐bipy)]*_n_*, Cd(H_2_L)(phen)(H_2_O)] and Cd(H_2_L)(4,4‐bipy)	^[^ ^285^ ^]^
Fe^3+^, Cr_2_O_7_^2−^ and nitrofurantoin in water	[TbL·2H_2_O]*_n_*	^[^ ^286^ ^]^
Fe^3+^, Cr_2_O_7_^2−^, MnO^4−^ and 4‐nitrophenol in water	[Ln_2_(L)_2_(H_2_O)_2_]·5H_2_O·6DMAC	^[^ ^287^ ^]^
Fe^3+^, CrO4^2−^ in water and acetone in DMF	Five Cd‐MOFs	^[^ ^288^ ^]^
Fe^3+^ and dichromate (Cr_2_O_7_^2−^) in water and picric acid in DMSO	Zr_6_(µ_3_‐O)_4_(µ_3_‐OH)_4_(OH)_4_(H_2_O)_4_(L)_2_	^[^ ^289^ ^]^
Al^3+^ and CO_3_^2−^ in water	[Tb(ppda)(ox)_0.5_(H_2_O)_2_]*_n_*	^[^ ^290^ ^]^
Metal ions	[Ca(H4L)(DMA)_2_]·2DMA (Ca‐MOF)	^[^ ^291^ ^]^
I^−^/S^2−^	Pt(II)TMPyP/rho‐ZMOF	^[^ ^292^ ^]^
Cr_2_O_7_^2−^	JLU‐MOF50	^[^ ^293^ ^]^
Cr_2_O_7_^2−^ in water	Zn_3_(L)(HL)(OH)(H_2_O)_3_]·H_2_O	^[^ ^294^ ^]^
Cr_2_O_7_^2−^	BUT‐39	^[^ ^295^ ^]^
CrO_4_^2−^/ Cr_2_O_7_^2−^	[Zn(DCTP)]·4.2H_2_O	^[^ ^296^ ^]^
CrO_4_^2−^, Cr_2_O_7_^2−^ and MnO_4_^−^	[Cd_1.5_(L)_2_(H_2_O)(NO_3_)]*_n_*, [Cd(L)_2_(H_2_O)_2_]*_n_*, [Pb(L)(H_2_O)_2_(NO_3_)]*_n_*, [Pb(L)_2_(H_2_O)]*_n_*, [Zn(L)_2_(H_2_O)_4_]*_n_*, [Zn_2_(L)(btc)(H_2_O)_2_]*_n_*, and [Zn(L)(p‐bdc)_0.5_(H_2_O)]·(p‐H_2_bdc)_0.5_|*_n_*	^[^ ^297^ ^]^
Cr_2_O_7_^2−^ in water *o*‐NP in ethanol	[Zn(opda)(pbib)] and [Zn(ppda)(pbib)(H_2_O)]	^[^ ^298^ ^]^
Nitroaromatic explosives	bio‐MOF‐1	^[^ ^299^ ^]^
Nitroaromatic explosives	UiO‐68@NH_2_	^[^ ^300^ ^]^
Nitroaromatic explosives	[Tb(L)(OH)]	^[^ ^301^ ^]^
5‐nitro‐2,4‐dihydro‐3*H*‐1,2,4‐triazole‐3‐one	TABD‐MOF‐1	^[^ ^302^ ^]^
2,4‐dinitrophenol and *p*‐benzoquinone	[Zn_4_(Hbpvp)_2_(BTC)_3_(HCOO)(H_2_O)_2_]	^[^ ^303^ ^]^
Hydrogen peroxide	Cu‐bipy‐BTC/carbon nanotube	^[^ ^304^ ^]^
H_2_S in water‐based biological and environmental assays	BFMOF‐1	^[^ ^305^ ^]^
TNP in water	[Co_2_(SDB)_2_(TIB)(H_2_O)](H_2_O)(1,4‐dioxane)_2_, Cd_3_(SDB)_3_(TIB)](H_2_O)_2_(1,4‐dioxane)(G)*_x_*	^[^ ^306^ ^]^
Sulfonamide antibiotics	FCS‐1	^[^ ^307^ ^]^
Nitrofuran, nitroimidazole, chloramphenicol, sulfonamide, and quinoline antibiotics	TMPyPE@bio‐MOF‐1	^[^ ^308^ ^]^
Nitro explosives and antibiotics in water	Zn(L)(aip)·(H_2_O), Zn(L)(ip)·(DMF)(H_2_O)_1.5_, Zn(L)(HBTC)·(H_2_O)_2_	^[^ ^309^ ^]^
Methyl red (MR) isomers (ortho, meta, and para) in ethanol	MIL‐100(Al)	^[^ ^310^ ^]^
Hippuric acid in urine	MIL‐121	^[^ ^311^ ^]^
Polycyclic aromatic hydrocarbons	Fe_3_O_4_@COF‐(TpBD)	^[^ ^312^ ^]^
Mycotoxin (3‐NPA)	FITC@ [Cd(L)·solvent]*_n_*	^[^ ^313^ ^]^
Anthocyanins from blackberries and methyl viologen in water	(CH_3_)_2_NH_2_]_9_Tb_6_(*η* ^6^TATAT)_4_(H_2_O)_12_·3Cl·DMA·7H_2_O	^[^ ^314^ ^]^
TNP	Cd_2_(Tipb)_2_(btc)Cl]·G*_x_*, [Cd(Tipb)(tdc)]·G*_x_*, [Cd(Tipb)(fdc)]·G*_x_* and [Cd_5_(Tipb)_4_(btc)_2_Cl_2_(DMF)_2_(H_2_O)_5_]·Cl_2_·G*_x_*	^[^ ^315^ ^]^
TNP	Zr_6_O_4_(OH)_4_(DCPB)_6_	^[^ ^316^ ^]^
VOCs (styrene, toluene, benzene, propylene and methanol)	Hybrid photonic‐MOF	^[^ ^317^ ^]^
Aromatic nitro‐explosives, aliphatic nitro‐explosives, antibiotics, un‐nitrated aromatic organic solvents	HPTS@ [Zn(TIPA)(NO_3_)_2_]·5H_2_O	^[^ ^318^ ^]^
Phenols and anilines in water	LVMOF‐1	^[^ ^319^ ^]^
Enantioselective sensing (proline)	S(R)‐ZIF‐78 h	^[^ ^320^ ^]^
Urea in human body fluids	Ni‐MIL‐77	^[^ ^321^ ^]^
Solvent molecules	PU‐1 and PU‐2	^[^ ^322^ ^]^
Bilirubin in human biofluids	1‐NH_2_@THB	^[^ ^323^ ^]^
Glucose in blood	PCN‐222(Fe)	^[^ ^324^ ^]^
PhCHO and Fe^2+^ in DMF	Cd_0.5_(TBC)]*_n_*	^[^ ^325^ ^]^
Cardiac troponin I (cTn) in human bio‐fluids	Mn–MOF‐PVC	^[^ ^326^ ^]^
*o*‐ and *p*‐TCBQ isomers	Tb@UiO‐66‐(COOH)_2_NH_2_	^[^ ^327^ ^]^
Pyrophosphate anion and antibiotics in water	ZTMOF‐1	^[^ ^328^ ^]^
Trichloroacetic acid in human urine	Fluorescein dye@ [Zn_2_(Tipa)_2_(OH)]·3NO_3_·12H_2_O	^[^ ^329^ ^]^
Alkaline phosphatase in human serum	MIL‐53(Fe)	^[^ ^330^ ^]^
DNA	Porphyrin‐cobalt(II) (Co‐TTPP)	^[^ ^331^ ^]^
Nitrobenzene and Fe^3+^ in methanol	[Cu(SCN)(hmp)]*_n_*	^[^ ^332^ ^]^
H_2_O_2_ in milk and human serum	Cu*_x_*ONPs/GF	^[^ ^333^ ^]^
Adrenaline in human serum	ZIF‐67/NC/3DG	^[^ ^334^ ^]^
Chlorpyrifos in water and food samples	Tb‐MOF@PDDA‐aggregated‐AuNP	^[^ ^335^ ^]^
Tyrosine in buffer solution and TNP in water	[Zr_6_O_4_(OH)_8_(H_2_O)_4_(L)_2_]·8DMF·10H_2_O	^[^ ^336^ ^]^
Cu^2+^ in water and nitro compounds in DMA	[Cd(Hcpnc)(DMF)]·DMF·2H_2_O	^[^ ^337^ ^]^
Nitroaromatic explosives in organic solvents	[Zn_4_(L)_1/3_(H_2_O)_4_]^6+^[S] [NaCd_2_(L)(DMF)_3_]^−^·(Me_2_NH_2_^+^)(3DMF)	^[^ ^338^ ^]^
C‐reactive protein in human serum	PCN‐777	^[^ ^339^ ^]^
Cholesterol in human serum	*β*‐cyclodextrin (*β*‐CMCD) grafted NU‐1000	^[^ ^340^ ^]^
Nitroaromatic compounds in DMF	[Cd(H_2_L)(bpz)]*_n_* and [Cd_2_(H_4_L)(L)(bpz)_2_]*_n_*	^[^ ^341^ ^]^
Arginine in urine	[Cd_4_(H_2_L)_2_(L)·H_2_O]·3H_2_O	^[^ ^342^ ^]^
Ampicillin‐binding aptamer in water	(CdS/Eu‐MOF)	^[^ ^343^ ^]^
Histamine and histidine in red wine and urine	Cu‐MOFs	^[^ ^344^ ^]^
n‐methylformamide in urine and methylglyoxal in serum	Tb_1−_ *_x_*Ybx@Cu‐Hcbpp	^[^ ^345^ ^]^
Macrophage cells	MOF‐1114(Yb) and MOF‐1140(Yb)	^[^ ^346^ ^]^
Nitrobenzene, benzene, and acetone	[Zn_2_(L)(bipy)(H_2_O)_2_]·(H_2_O)_3_(DMF)_2_	^[^ ^347^ ^]^ (DFT)
Benzene and nitrobenzene	Zn(L)(HDMA)_2_(DMF)(H_2_O)_6_	^[^ ^348^ ^]^ (DFT)
Toulene, dioxane, methanol, and hexane	Gallium‐based COMOC‐4	^[^ ^349^ ^]^(DFT)
Al^3+^ in water	Zn(HTABDC)(DMF)_2_, Zn(HTABDC)(Bpy)·DMF	^[^ ^350^ ^]^ (DFT)
Mandelic acid, 1,2‐cyclohexanediamine, 1‐phenylethylamine and 1‐phenylpropylamine	Zn_6_(salalen)_6_, Zn_6_(salalen)_3_(salen)_3_ and Zn_6_(salen)_6_	^[^ ^351^ ^]^ (exp./DFT)
VOCs and metal ions	NUS‐24	^[^ ^352^ ^]^ (exp./DFT)
Amino acids, pharmaceutical molecules	COFs (NUS 30–32)	^[^ ^353^ ^]^ (exp./DFT)
Live‐cell	NUS‐100, NUS‐101	^[^ ^354^ ^]^ (exp./DFT)
Fe^3+^ in water	ZJU‐109	^[^ ^355^ ^]^ (exp./DFT)
Amines	F‐CTF	^[^ ^356^ ^]^ (exp./DFT)

Lanthanide (Ln) ions are widely employed as lumophores into functional supramolecular scaffolds of MOFs because of their spectrally narrow emission even in a solution. Thus, the first application of sensing in a liquid medium was based on Ln‐MOFs. Zhao et al. reported two isostructural 3d‐4f heterometallic coordination polymers [Ln(PDA)_3_Mn_1.5_(H_2_O)_3_]·3.25H_2_O (Ln = Eu^3+^ and Tb^3+^, PDA = pyridine‐2,6‐dicarboxylic acid) from two building blocks Ln(PDA)_3_ and MnO_4_(H_2_O)_2_. Each adjacent building block was connected with carboxyl groups of PDA to form a highly ordered framework with 1D channels of about 1.8 nm diameter, which was achieved by a dodecanuclear heterometallic macrocycle of Ln_6_Mn_6_C_12_O_24_ with a C_6_ symmetry. The luminescence of these Ln‐MOFs was measured in DMF solutions by introducing metal ions such as Zn^2+^, Mn^2+^, Ca^2+^, Mg^2+^, Fe^2+^, Co^2+^, and Ni^2+^. Only in the presence of Zn^2+^, the emission intensity was observed to increase at least twice of that in DMF solutions without Zn^2+^.^[^ ^245^ ^]^ Zhao et al. also prepared isomorphic Dy‐Mn MOF of Eu (or Tb)‐MN and tested for their luminescent behavior. These new MOFs were found to selectively sense Mg^2+^ rather than Zn^2+^.^[^ ^246^ ^]^

**Figure 28:**
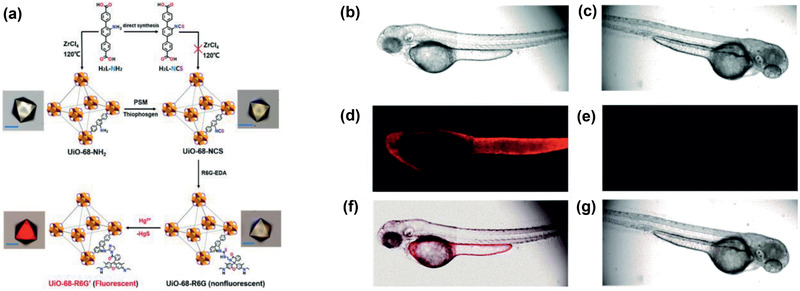
a) Synthesis of UiO‐68‐NCS, UiO‐68‐R6G and UiO‐68‐R6G’. b,c) bright field; d,e) dark‐field; f,g) overlaid images of a 3‐day‐old zebrafish treated with nanosized UiO‐68‐R6G. Reproduced with permission.^[^ ^260^ ^]^ Copyright 2019, Royal Society of Chemistry.

Majority of the initial sensing studies were limited to organic media. With more stable MOFs synthesized, sensing has been extended to aqueous and biological media. Several water‐stable MOFs hold great potential to sensing environmentally polluting cations in water. Chen and co‐workers reported the first example of luminescent MOF, Eu_2_(FMA)_2_(OX)(H_2_O)_4_·4H_2_O (FMA = fumarate; OX = oxalate), for highly selective sensing of Cu^2+^ in aqueous solutions.^[^ ^255^ ^]^ From a stepwise post‐synthetic modification (PSM) process starting from UiO‐68‐NH_2_ in a single‐crystal‐to‐single‐crystal (SCSC) manner, as shown in **Figure** **28**a, Li et al. synthesized UiO‐68‐NCS, UiO‐68‐R6G and UiO‐68‐R6G’. UiO‐68‐R6G with rhodamine‐based moiety was observed to be highly sensitive and specific chemodosimeter to detect Hg^2+^ via both visual and fluorogenic observations. They successfully used nanoscale UiO‐68‐R6G for the detection of Hg^2+^ in HeLa cells and zebrafish as demonstrated in Figure 28b–g.^[^ ^260^ ^]^ On the other hand, iron plays a crucial role in biological systems as oxygen carrier in blood stream. Thus, many Ln‐MOFs were examined for sensing biological relevant Fe^3+^ and Fe^2+^. Li and co‐workers developed Eu‐MOF/EDTA‐NiAl‐CLDH fluorescent micromotor, which was ethylene diamine tetraacetic acid (EDTA) functionalized and comprised calcined layered double hydroxides (CLDH), for sensing and removal of Fe^3+^ from water. Compared with conventional static MOFs, the self‐propelled micromotors were found to greatly enhance sensing efficiency because the actively moving material could convert chemical energy or other forms of energies into kinetic energy.^[^ ^270^ ^]^

It is also important to recognize and sense anions in organic solvents as well in water. A Tb‐mucicate framework [Tb(Mucicate)_1.5_(H_2_O)_2_]·5H_2_O synthesized by Wong et al. showed reversible anion uptake and sensing in aqueous solution. The Tb‐centered luminescent intensity was enhanced in the presence of halides, CN^−^ and CO_3_^2−^, but not of SO_4_^2−^ and PO_4_^3−^. This was attributed to the size exclusion of SO_4_^2−^ and PO_4_^3−^ being unable to fit into the framework channels.^[^ ^277^ ^]^ Similar anion size‐based intensity enhancement was observed in Zn‐ and Cd‐MOFs containing 4,4′‐bipyridine (bipy) and p‐aminobenzoatein (paba) linkers for PO_4_^3−^, as its large size allowing more strong interaction through hydrogen bonding with the framework.^[^ ^278^ ^]^ Nevertheless, small sized NO_2_^−^ rather than other oxoacid anions, was found to cause selective quenching of luminescence in Cd(µ_2_‐Cl)(µ_4_‐5‐methyl‐1H‐tetrazole), where the size and shape of cavities allowing the diffusion of small ions.^[^ ^279^ ^]^ Chen et al. synthesized Tb‐MOF (MOF‐76) with 1D channels of about 6.6 × 6.6 Å^2^ partially occupied by methanol molecules. This MOF exhibited excellent selective sensing of F^−^ with high luminescence enhancement, as a result of strong hydrogen‐bonding between F^−^ and methanol molecules in the channels.^[^ ^280^ ^]^

**Figure 29:**
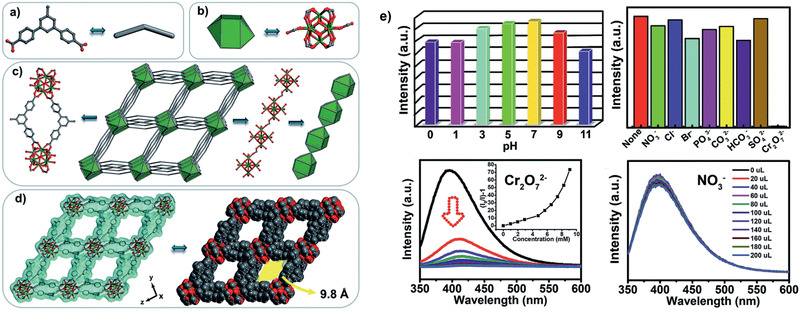
JLU‐MOF50: a–d) topology simplification of V‐shaped ligand and Zr_6_ cluster, polyhedron and space filling representation; e) Luminescence intensity of JLU‐MOF50 after treated in aqueous solutions with different pH values and different anions. Reproduced with permission.^[^ ^293^ ^]^ Copyright 2018, Royal Society of Chemistry.

On the other hand, many metal pollutants exist in the form of oxo‐hydroxo anions such as SeO_3_^2−^, Cr_2_O_7_^2−^, TcO^4−^, and AsO_4_^3−^. Among these, Cr_2_O_7_^2−^ is a prominent concern as it can accumulate in living organisms and cause serious damages. Sun et al. reported an ultrastable Zr‐MOF (JLU‐MOF50, **Figure** **29**a–d) with ligand H_2_MDCPB = 1,3‐di(4‐carboxyphenyl) benzene connected to 10‐connected Zr_6_ clusters, providing luminescence and highly porous nature. Excellent chemical stability was found for this MOF in both acidic and alkaline aqueous solutions. More interestingly, it exhibited not only a notable trapping capacity (92 mg g^−1^) and a rapid adsorption rate (32.5 mg g^−1^ min^−1^) of Cr_2_O_7_^2−^, but also highly selective sensing of Cr_2_O_7_^2−^ in aqueous phase (Figure 29e). The *K*_SV_ value (quenching constant) was measured up to 4.99 × 10^4^ m ^−1^, one of the highest values reported to date for MOFs. The dual functionalities were attributed to missing linker, high surface area, and excellent luminescence property. This study suggests that JLU‐MOF50 is a good candidate for both rapid capture and highly luminescence detection of Cr_2_O_7_^2−^ in aqueous phase.^[^ ^293^ ^]^

**Figure 30:**
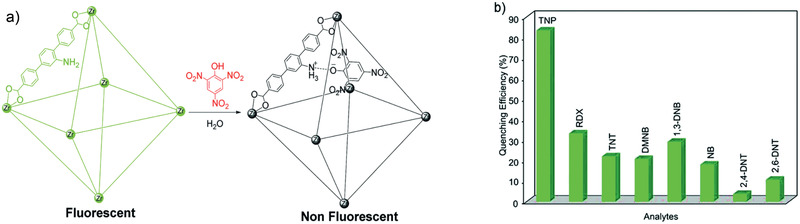
a) Pendant amine functionality for fluorescence. b) Comparison of fluorescence quenching efficiency of different nitro analytes toward activated UiO‐68@NH_2_ in water. Reproduced with permission.^[^ ^300^ ^]^ Copyright 2015, Royal Society of Chemistry.

Nitro explosives in aqueous solutions pose a high threat to land security and environmental pollution, thus prompting a high demand of their detection. Ghosh and co‐workers reported chemically stable UiO‐68@NH_2_ with a pendant recognition site as shown in **Figure** **30**a for selective detection of nitro‐aromatic explosive 2,4,6‐trinitrophenol (TNP) in aqueous solutions. The pendant Lewis basic amine moieties were implanted to interact with TNP via electrostatic interaction and acted as recognition sites for TNP. By using this MOF after activation, the presence of TNP in water was detected at a concentration as low as 0.4 ppm, with a response time of a few seconds even in the presence of competing nitro analytes. Due to the visible excitation and emission wavelengths, UiO‐68@NH_2_ would be an ideal candidate for TNP sensing. The outstanding and selective quenching ability towards TNP was demonstrated by a high quenching constant (5.8 × 10^4^ m ^−1^), about 23 times higher than those for 2,4,6‐trinitrotoluene (TNT) and hexahydro‐1,3,5‐trinitro‐1,3,5‐triazine (RDX) as plotted in Figure 30b. This unparalleled selectivity was attributed to electron‐transfer and energy‐transfer mechanisms, as well as electrostatic interaction between the MOF and TNP. To drive for practical application, a MOF‐coated paper strip was prepared and the same fast and selective response to TNP in water was observed.^[^ ^300^ ^]^

**Figure 31:**
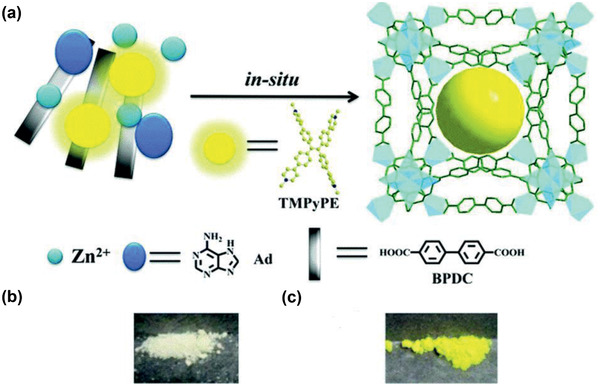
a) Encapsulation of TMPyPE into bio‐MOF‐1. Colors b) before and c) after encapsulation. Reproduced with permission.^[^ ^308^ ^]^ Copyright 2019, Royal Society of Chemistry.

While increasingly used to treat infection of animals and plants, antibiotics have high potential to enter surface and ground waters. Thus, there is strong interest in sensing antibiotics and other related pharmaceutical compounds. A luminescent MOF [Zn_8_(C_5_H_4_N_5_)_4_(C_14_H_8_O_4_)_6_O(C_50_H_44_N_4_)_0.5_] (TMPyPE@bio‐MOF‐1) was prepared by in situ encapsulation of cationic tetraphenylethene (TPE) based tetrakis[4‐(1‐methylpyridin‐4‐yl)phenyl]ethene (TMPyPE) into an anionic MOF, as depicted in **Figure** **31**. This MOF exhibited high stability and strong fluorescence in water and could selectively sense nitrofuran antibiotics in aqueous solutions. TMPyPE@bio‐MOF‐1 was found to have higher fluorescence quenching efficiencies of 96% for nitrofurazone (NFZ) and 95.5% for nitrofurantoin (NFT) than other antibiotics at the same titration concentration. Because of the simplicity in preparation and sensitivity toward anitobiotics, this TPE‐based luminogen encapsulated MOF would have wide potential sensing applications.^[^ ^308^ ^]^

Apart from luminescence based sensing, other transducing methods have also been implemented into MOFs for sensing in liquid phase. Bao et al. recently prepared a Ni‐MOF decorated electrode in differential pulse voltammetry (DPV) to analyze urea in human body samples.^[^ ^321^ ^]^ By embedding nitrogen‐rich‐carbon‐coated ZIF‐67 into 3D‐graphene (ZIF‐67/NC/3DG) fiber, Zeng et al. developed an electrode to detect adrenaline from human serum.^[^ ^334^ ^]^ A novel photoelectrochemical (PEC) assay was fabricated to sense C‐reactive protein using PCN‐777 as a photoelectric material and thioflavin‐T (Th‐T) as a signal sensitizer coupled with rolling circle amplification.^[^ ^339^ ^]^

Various mechanisms are potentially involved in the intrinsic luminescence of MOFs such as metal‐centered emission, ligand‐centered emission, ligand‐to‐metal charge transfer, metal‐to‐ligand charge transfer, and interligand charge transfer (LLCT). Moreover, guest induced emission may also occur via either encapsulation of luminescent molecules or exciplex formation. Understanding the physiochemical nature of both probe and analyte is imperative for the design of effective sensors. Particularly, quantitative evaluation of electronic states like the highest occupied molecular orbital (HOMO) and the lowest occupied molecular orbital (LUMO) is useful in elucidating and predicting electron transfer for enhancing (turn‐on) or quenching (turn‐off) fluorescence response. However, experimentally identifying emission routes and discerning between competing processes are usually difficult. In this aspect, computation is an invaluable tool to provide microscopic insight and facilitate the design of new high‐performance luminescent MOFs.

Based on DFT calculations, Hao and co‐workers investigated the detection mechanism of nitroaromatics by luminescent [Zn_2_(L)(bipy)(H_2_O)_2_]·(H_2_O)_3_(DMF)_2_, which is insoluble in acetonitrile suspension. The molecular orbitals involved in excitation process were confirmed by excitation energy. The fragment orbitals related to excitation were examined to understand analyte–sensor interactions.^[^ ^347^ ^]^ To include the effect of solvents, they further implemented a polarization continuum model (PCM) in DFT calculations; however, the solvent effects were not included for insoluble MOFs.^[^ ^348^ ^]^ Liu et al. used a PCM solvent model in DFT calculations to examine solvent dependent luminescence of a gallium MOF (COMOC‐4). The UV–vis spectra calculated were in excellent agreement with experimental data.^[^ ^349^ ^]^ Li et al. investigated aggregation‐induced emission (AIE) induced luminescence in two different MOFs both containing AIE‐active fluorophore HTABDC as a ligand. One MOF was found to be highly emissive, whereas the other with auxiliary ligand Bpy as a quenching agent was almost nonemissive. From DFT calculations, the most important molecular orbitals in these two MOFs were identified to illustrate the difference in HOMO–LUMO gaps and emission behavior.^[^ ^350^ ^]^

**Figure 32:**
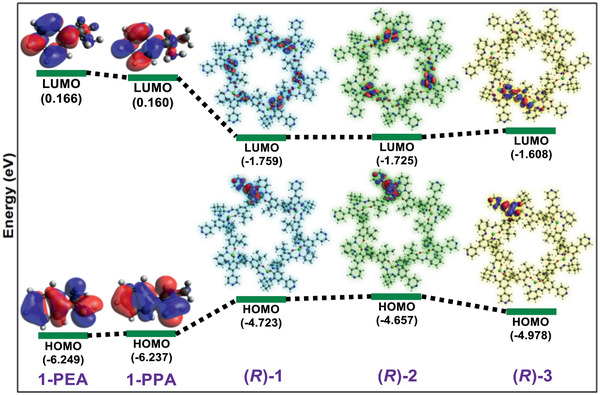
HOMO–LUMO energy profiles of 1‐PEA, 1‐PPA, (R)‐1, (R)‐2, and (R)‐3. Reproduced with permission.^[^ ^351^ ^]^ Copyright 2017, American Chemical Society.

By assembling Zn(salalen) and Zn(salen) building units, Cui and co‐workers prepared three chiral NH‐controlled metallacycles (*R*)‐1 Zn_6_(salalen)_6_, (*R*)‐3 Zn_6_(salalen)_3_(salen)_3_, and (*R*)‐2 Zn_6_(salen)_6_. The chiral cavities and NH groups in (*R*)‐1–3 were able to enantioselectively recognize *α*‐hydroxycarboxylic acids such as mandelic acid (MA) and sugar acids; they could also discriminate the enantiomers of sugar acids and amino acids through fluorescence quenching in a solution and the enantiomers of 1‐phenylethylamine (1‐PEA) through fluorescence enhancement in a crystalline state. From single‐crystal X‐ray diffraction and DFT calculations, the intrinsic chiral recognition and discrimination were attributed to the specific binding of enantiomers in the chiral microenvironment of (*R*)‐1–3. The selectivity and binding affinity were found to be greatly affected by the available NH groups in the metallacycles. The enantioselective fluorescence change was also explained by the donor–acceptor electron‐transfer mechanism as shown in **Figure** **32**. Furthermore, manipulation of chiral NH functionalities in the metallacycles could control the enantiorecognition of biomolecular complexes, which might provide a new opportunity for the assembly of more effective chiral supramolecular materials for enantioselective processes.^[^ ^351^ ^]^

**Figure 33:**
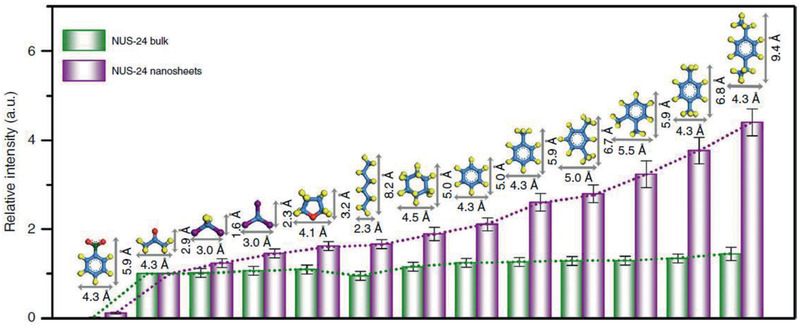
Relative fluorescence intensity of bulk and nanosheets of NUS‐24 in various VOC solutions. The error bars show average and range of measured values based on at least five repeats in every single measurement. Reproduced with permission.^[^ ^352^ ^]^ Copyright 2017, Springer Nature.

Using flexible tetraphenylethylene (TPE) units as molecular rotors, Zhao and co‐workers synthesized all‐carbon, *π*‐conjugated 2D porous organic nanosheets (NUS‐24) with high stability, large lateral size and ultrathin thickness (2–5 nm). The dynamic TPE rotors exposed on the surface of NUS‐24 nanosheets could be restricted in an aggregated state with different water fractions, thereby leading to size‐selective turn‐on fluorescence by VOCs. As demonstrated in **Figure** **33**, NUS‐24 nanosheets were observed to exhibit stronger turn‐on fluorescence than bulk NUS‐24 upon contact with electron‐rich VOCs, and the fluorescence enhancement was positively correlated with the molecular size of VOC molecules. This was attributed to the restriction of liberated TPE rotors on the external surface of NUS‐24 nanosheets when interacting with VOC molecules. Meanwhile, DFT calculations revealed a donor–acceptor electron transfer mechanism for the observed fluorescence quenching.^[^ ^352^ ^]^ They further synthesized three azine‐linked and imine‐linked 2D COFs (NUS 30–32) using monomers containing AIE rotor‐active TPE moieties. The COF nanosheets showed signal amplification in biomolecular recognition of amino acids and small pharmaceutical molecules, exhibiting much higher sensitivity than their stacked bulk powders, TPE monomer and COF model compound.^[^ ^353^ ^]^ These studies suggest the potential applications of 2D materials containing molecular rotors in explosive detection and environmental monitoring.

**Figure 34:**
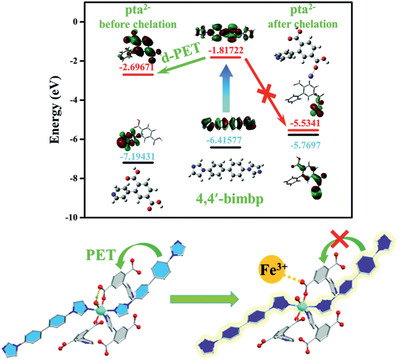
a) HOMO and LUMO energy levels of 4,4′‐bimbp and pta^2−^. b) PET process and turn‐on sensing of Fe^3+^. Reproduced with permission.^[^ ^355^ ^]^ Copyright 2019, Royal Society of Chemistry.

Recently, Qian and co‐workers measured photo‐induced electron transfer (PET) in ZJU‐109 as a fluorescent turn‐on sensor for Fe^3+^ in aqueous solutions, then used DFT calculations to explain PET process between 4,4′‐bimbp and pta^2−^. As depicted in **Figure** **34**, ZJU‐109 was observed to show relatively weak fluorescence originating from 4,4′‐bimbp because of efficient intermolecular PET process between 4,4′‐bimbp and pta^2−^. In the presence of Fe^3+^ ions, the emission intensity was increased as a result of blocked quenching process, thus demonstrating its interesting application as a highly selective luminescent turn‐on sensor for Fe^3+^ with a detection limit of 0.053 × 10^−6^ m. Compared with traditional molecular sensors, the separated and ordered arrangement of ligands in ZJU‐109 could prohibit aggregation‐caused quenching. By integrating fluorescent and receptor ligands in a single MOF, this study opens up a new avenue to develop various luminescent turn‐on sensors for ions and small molecules.^[^ ^355^ ^]^

With several possible sensing mechanisms (e.g., photoluminescence, electrochemical, and mechanical), MOFs have received increasing attention for chemical sensing. In addition, highly selective sensitivity under competing compounds and precise detection from very low concentrations are intriguing features of MOFs compared with other materials. MOFs with multifunctionality of sensing as well as capture/degradation of harmful compounds are rapidly growing due to the strong interest in complex practical applications. Most of the studies reported in Table 5 were for the direct use of powders of luminescent MOFs, which could act as standalone sensors to recognize and produce signals. Nevertheless, other forms such as thin films and fibers derived from nanoscale MOFs (nanoMOFs) have been combined with external transducers to produce electrochemical or mechanical sensing devices.^[^ ^321^, ^334^, ^339^ ^]^

## 4 Pharmaceutical Sector

### 4.1 Chiral Separation

Majority of naturally occurring or manmade bioactive molecules are chiral enantiomers. For a pair of enantiomers, one may exhibit useful pharmacological activity and the other may be neutral or even harmful. With overwhelming increasing use of chiral compounds as therapeutic drugs, agrochemicals and food flavorings, the separation of enantiomers is crucial. However, chiral separation is challenging because two enantiomers have identical chemical and physical properties in a symmetric environment. Among available methods, liquid chromatography is considered as efficient technique in which chiral stationary phases (CSPs) are the key to discriminate enantiomers.^[^ ^357^, ^358^ ^]^ In the past, CSPs were largely based on natural bio‐organic compounds and a small fraction on functionalized zeolites. Recently, chiral MOFs have been developed by incorporating chiral ligands into frameworks. Particularly, homochiral MOFs can be readily produced and used for chiral separation, as summarized in **Table** **6**.

**Table 6:** Chiral Separation

Racemic mixture	MOF	Ref.
[Ru(2,2‐bipy)_3_]Cl_2_	L‐POST‐1	^[^ ^359^ ^]^
1,2‐diaminocyclohexane	Ln(L‐H_2_)(L‐H_3_)(H_2_O)_4_	^[^ ^360^ ^]^
2‐butanol, 2‐mehtyl‐1‐butanol	Cd(QA)_2_	^[^ ^361^ ^]^
2‐butanol	Cu_5_Cl_6_(VB‐N‐CIN)_2_	^[^ ^362^ ^]^
Menthone, fenchone, binaphthol	Ni_3_(btc)_2_(3‐pic)_6_(1,2‐pd)_3_	^[^ ^363^ ^]^
1‐phenylethanol	[CdL_2_(H_2_O)_2_](ClO_4_)_2_·2DMF·3EtOH·5/3H_2_O	^[^ ^364^ ^]^
Diols	Ni_2_(*L*‐asp)_2_(bipy)	^[^ ^365^ ^]^
2‐butanol	Cu(PPh_3_)(N,N’‐(2‐pyridyl‐(4‐pyridyl methyl)‐amine)_1.5_⋅ClO_4_	^[^ ^366^ ^]^
Sulfoxides	Zn_2_(bdc)(*L*‐lac)(dmf)	^[^ ^367^ ^]^
Alkyl aryl sulfoxides	Zn_2_(bdc)(*L*‐lac)(dmf) (HPLC)	^[^ ^368^ ^]^
2‐naphSOMe, PhSOCH_2_Ph	Zn_2_(ndc)(*R*‐man)(dmf), Zn_2_(bpdc)(*R*‐man)(dmf)	^[^ ^369^ ^]^
2‐butanol, 2‐methyl‐1‐butanol, 1‐phenyl‐1‐ethanol, 1‐phenyl‐1‐propanol	Zn_2_(bdc)(*S*‐lac)(dmf)	^[^ ^370^ ^]^
2‐butanol, 2‐mehtyl‐2‐butanol, 2‐pentanol	ZnL	^[^ ^371^, ^372^ ^]^
2‐butanol, 3‐mehtyl‐2‐butanol, 2‐pentanol	Cu_2_L_2_	^[^ ^373^ ^]^
2‐butanol, 3‐methyl‐2‐butanol	M_4_L_6_	^[^ ^374^ ^]^
1‐phenylethyl alcohol	Zn_3_(BDC)_3_[Cu(SalPycy)], Zn_3_(CDC)_3_[Cu(SalPycy)]	^[^ ^375^ ^]^
16 sulfoxides	CuMOF‐1‐silica composite (HPLC)	^[^ ^376^ ^]^
2,5‐hexandiol	SURMOF	^[^ ^377^ ^]^
Alcohol, ketone, flavone, phenol, base, and amide racemates	(CH_3_)_2_NH_2_][Cd(bpdc)_1.5_]·2DMA (HPLC)	^[^ ^378^ ^]^
Secondary alcohols	HOF‐2	^[^ ^379^ ^]^
2‐butanol, 1‐phenylethanol	Chir‐UMCM‐1 (HPLC)	^[^ ^380^ ^]^
Alcohol, phenol, aldehyde, ketone, base, amide, and a chiral drug	[Zn_2_(d‐Cam)_2_(4,4′‐bpy)]*_n_* (HPLC)	^[^ ^381^ ^]^
Three alcohols, a diol, a naphthol, and a ketone	[Cu_2_(d‐Cam)_2_(4,4′‐bpy)]*_n_* (LC)	^[^ ^382^ ^]^
3‐benzyloxy‐1,2‐propanediol, 3,5‐dinitro‐*N*‐ (1‐henylethyl) benzamide, praziquantel, *trans*‐stilbene oxide, 1‐ (1‐naphthyl)ethanol, Tröger's base, positional isomers	Co_2_(d‐cam)_2_(TMDPy) (LC)	^[^ ^383^ ^]^
Ibuprofen, 1‐phenyl‐1‐propanol, benzoin, ketoprofen, naproxen	[ZnLBr]·H_2_O*_n_*	^[^ ^384^ ^]^
1‐phenyl‐2,2,2‐trifluoroethanol	DUT‐32‐NHProBoc	^[^ ^385^ ^]^
Ibuprofen, 1‐phenyl‐1,2‐ethanediol	(Me_2_NH_2_)_2_[Mn_4_O(d‐cam)_4_]·(H_2_O)_5_	^[^ ^386^ ^]^
1‐ (1‐naphthyl)ethanol, 1‐ (4‐chlorophenyl)ethanol, 1‐ (9‐anthryl) ‐2,2,2‐trifluoroethanol, 1,1’‐bi‐2‐naphthol, benzoin, hydrobenzoin, trans‐stilbene oxide, praziquantel, and warfarin sodium	[Cd_2_(d‐cam)_3_]·2Hdma·4dma	^[^ ^387^ ^]^
Sulfoxides, *sec*‐alcohols, *β*‐lactams, benzoins, flavanones, and epoxides	CuMOF‐1–silica composite, (R)‐ZnMOF‐1–silica composite, and (R)‐CuMOF‐2–silica composite	^[^ ^388^ ^]^
Limonene and 1‐phenylethanol	CD‐MOF	^[^ ^389^ ^]^
2‐methyl‐2,4‐pentanediol	Ni_2_(*L*‐asp)_2_(bipy) (Ni‐LAB membrane on ceramic support)	^[^ ^390^ ^]^
2‐methyl‐2,4‐pentanediol	Ni_2_(*L*‐asp)_2_(bipy) (Ni‐LAB pervaporation)	^[^ ^391^ ^]^
3‐methoxyphenyl methyl sulfoxide	Zn‐DHIP MOF, Cd‐DHIP MOF	^[^ ^392^ ^]^
Alcohols, amines, ketones, ethers, organic acids	[Zn(L‐tyr)]*_n_*(L‐tyrZn), [Zn_4_(btc)_2_(Hbtc)(L‐His)_2_(H_2_O)_4_]·1.5H_2_O,Zn_2_(Ltrp)_2_(bpe)_2_(H_2_O)_2_](NO_3_)_2_·2H_2_O, [Co_2_(L‐Trp)(INT)_2_(H_2_O)_2_(ClO_4_)], [Co_2_(sdba)(L‐Trp)_2_], [Co(L‐Glu)(H_2_O)·H_2_O]	^[^ ^393^ ^]^
1‐phenylethanol or 2‐butanol	[Zn_2_(bdc)(*L*‐lac)(dmf)]	^[^ ^394^ ^]^
Alcohols and sulfoxides	[Cd_2_L(DMF)_2_(H_2_O)]·DMF·3H_2_O	^[^ ^395^ ^]^
Alcohols, sulfoxides, carboxylic acids, and esters	chiral COFs	^[^ ^396^ ^]^
Methyl phenyl sulfoxide	Zn_2_(bdc)(*L*‐lac)(dmf) membrane	^[^ ^397^ ^]^ (sim.)
1,3‐dimethylallene, 1,2‐dimethylcyclobutane, 1,2‐dimethylcyclopropane	Cd‐BINOL	^[^ ^398^ ^]^ (sim.)
19 compounds	9 homochiral MOFs	^[^ ^399^ ^]^ (sim.)
Metamphetamine and ephedrine	Cu(GHG) peptide MOF	^[^ ^400^ ^]^ (sim.)
Aromatic and aliphatic amines	1,10‐biphenol‐based MOFs	^[^ ^401^ ^]^ (sim.)
2‐butanol and 2‐butylamine	Zn_6_(salalen)_6_, Zn_6_(salalen)_3_(salen)_3_, and Zn_6_(salen)_6_	^[^ ^351^ ^]^ (sim.)

Lin and co‐workers synthesized a series of stable homochiral MOFs (lamellar lanthanide bisphosphonates) and determined their crystal structures. The MOFs were then packed in a flash chromatography for the chiral separation of racemic *trans*‐1,2‐diaminocyclohexane from a methanol/triethylamine mixture. In the beginning fractions, (*S*,*S*)‐1,2‐diaminocyclohexane was observed with enantiomeric excess (*ee*) of 13.6%; however, (*R*,*R*)‐1,2‐diaminocyclohexane was detected in the ending fractions with *ee* of 10.0%.^[^ ^360^ ^]^ Meanwhile, Xiong et al. produced a zeolite‐analogue diamondoid MOF, Cd(QA)_2_, composed of Cd^2+^ ions and enantiopure chiral quitenine linker. With intra‐framework voids, Cd(QA)_2_ was shown to provide a chiral environment for guest molecules. Upon mixing powdered Cd(QA)_2_ sample with racemic 2‐butanol and 2‐methyl‐1‐butanol, respectively, (*S*)‐enantiomers were found to be included in Cd(QA)_2_. The *ee* was estimated to be 98.2% for 2‐butanol and 98.4% for 2‐methyl‐1‐butanol.^[^ ^361^ ^]^

A homochiral MOF with 47% permanent porosity was reported by Rosseinsky and co‐workers and tested for the enantioselective sorption of menthone, fenchone, and binaphthol. While *ee* of 8.3% was obtained for binaphthol, no enantioselection was observed for larger menthone and fenchone. Thus, the enantioselectivity was found to be strongly dependent on guest size.^[^ ^363^ ^]^ Later, they synthesized a family of MOFs formed by Ni^2+^ and *L*‐aspartic acid (*L*‐asp). With 1D channels of 3.8 × 4.7 Å, Ni_2_(*L*‐asp)_2_(bipy) was composed of chiral Ni(*L*‐asp) layers connected by 4,4’‐bipyridine linkers to afford a pillared structure. Enantioselective sorption in this MOF was tested for a library of diols. At 278 K, the highest *ee* was measured to be 53.7% for 2‐methyl‐2,4‐pentanedoil. Strikingly, diols with similar chain lengths but differing locations of hydroxyl groups exhibited different levels of enantioselection.^[^ ^365^ ^]^

**Figure 35:**
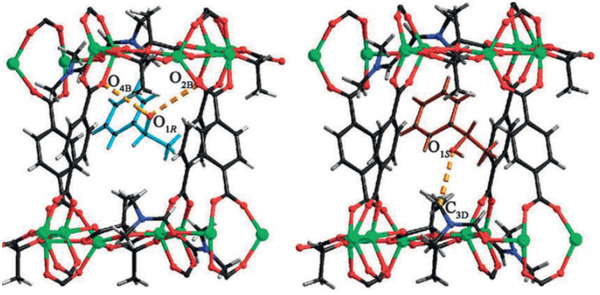
Locations of *R*‐PhEtOH and *S*‐PhEtOH in Zn_2_(bdc)(*S*‐lac)(dmf). Reproduced with permission.^[^ ^370^ ^]^ Copyright 2012, Royal Society of Chemistry.

Homochiral Zn_2_(bdc)(*L*‐lac)(dmf) with chiral centers of *L*‐lactate (*L‐*lac) moieties exposed on interconnected pores was prepared by Dybtsev et al. The MOF was examined for the chiral separation of several substituted sulfoxides dissolved in CH_2_Cl_2_. The *ee* value was determined between 20% and 27% with the *S*‐enantiomer more preferentially sorbed. In addition, remarkable catalytic activity with size‐ and chemo‐selectivity was also observed.^[^ ^367^ ^]^ The Zn_2_(bdc)(*L*‐lac)(dmf) was developed as a stationary phase in a chromatographic column, for the first time, to separate racemic alkyl aryl sulfoxides. Clear peak resolutions were demonstrated, indicating the efficient separation of enantiomers. Furthermore, the separation capability was uniquely combined with catalytic reaction for the isolation of both *R*‐ and *S*‐enantiomers in an one‐pot procedure.^[^ ^368^ ^]^ Dybtsev et al. further synthesized Zn_2_(bdc)(*S*‐lac)(dmf) and examined for the enantioselective sorption of chiral alcohols. The *ee* values for different alcohols were relatively modest with the highest of 21%. A detailed analysis was conducted for PhEtOH‐host complexes. The inclusion of 1‐phenyl‐1‐ethanol (PhEtOH) molecules was found not to cause any notable distortion of original host structure, as shown in **Figure** **35**, with both *R*‐ and *S*‐PhEtOH occupying similar position. Nevertheless, a notable difference was observed in the direction of OH group of PhEtOH, which was speculated to drive the modest enantioselective sorption.^[^ ^370^ ^]^

Cui and co‐workers reported a large arrays of cyclic clusters with enzyme‐like cavities by efficient assembly of a nanoscale metallacyle ZnL. The chiral and dynamic cavities exhibited high enantioselectivity for the separation of racemic alcohols. For 2‐butanol, 2‐mehtyl‐2‐butanol and 2‐pentanol, the *R*‐enantiomers were found to be preferentially adsorbed with *ee* values > 99.5%. This study suggests that the ready tunability of molecular building‐block approach would be promising for the development of unique and useful chiral adsorbents.^[^ ^372^ ^]^ In a later study, Cu_2_L_2_ with a chiral 2D corrugated network was prepared with large asymmetric channels of 6.93 Å. High *ee* values > 99.5% were observed for the separation of 2‐butanol, 3‐mehtyl‐2‐butanol and 2‐pentanol, while C_6_ and C_7_ alcohols were excluded in the network. In addition, the Cu_2_L_2_ host adsorbed with different alcohols was found to exhibit guest‐responsive ferroelectric behavior.^[^ ^373^ ^]^

**Figure 36:**
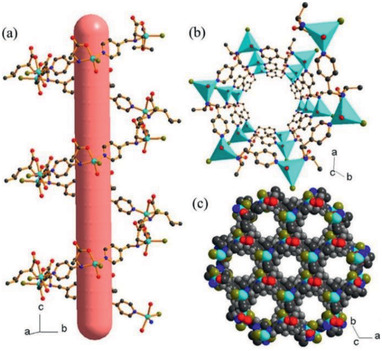
a) A single helical chain in ([ZnLBr]·H_2_O)*_n_*. b) Top view of a single helical chain. c) Space‐filling diagram of ([ZnLBr]·H_2_O)*_n_* network. Reproduced with permission.^[^ ^384^ ^]^ Copyright 2014, American Chemical Society.

A modular approach was developed by Padmanaban et al. for the synthesis a chiral analogue of UMCM‐1 (Chir‐UMCM‐1). The framework structure, topology and pore shape in both Chir‐UMCM‐1 and UMCM‐1 were found to be similar. Chir‐UMCM‐1 was implemented as a stationary phase for a HPLC column with *n*‐heptane as a mobile phase to separate racemic 2‐butanol and 1‐phenylethanol, respectively. Significant enantioselection was observed compared with a column packed with UMCM‐1. The separation selectivity and resolution of 1‐phenylethanol were estimated as 1.6 and 0.65, respectively. To further improve efficiency, it was suggested to use adapted column length or a gradient elution.^[^ ^380^ ^]^ Kuang et al. reported a 3D noninterpenetrated stable homochiral MOFs ([ZnLBr]·H_2_O)*_n_* containing 1D helix channel as shown in **Figure** **36** formed by using an enantiopure pyridyl‐functionalized salen [(N‐(4‐Pyridylmethyl)‐*L*‐leucine·HBr)]. When tested as a chiral stationary phase for HPLC, the material showed excellent performance in enantioseparation of drugs. A high‐resolution enantioseparation of (±)‐ibuprofen was observed with a good selectivity factor of 2.4 and the elution sequence was left‐handed followed by right‐handed. This MOF could be regarded as a novel chiral material possessing the size based separation function to achieve molecular sieve‐like enantioseparation.^[^ ^384^ ^]^

**Figure 37:**
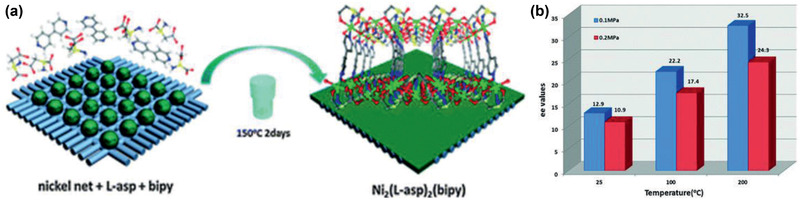
a) A homochiral MOF membrane preparation by in situ growth on a nickel net. b) *ee* values for racemic diol mixtures. Reproduced with permission.^[^ ^391^ ^]^ Copyright 2013, Royal Society of Chemistry.

The studies discussed above for chiral separation used MOFs as either adsorbents or stationary phases. Alternatively, MOFs can be fabricated into membranes for chiral separation. This is attractive because of low cost, high capacity, continuous operation of membrane separation. Via a method of single metal source, Qiu and co‐workers fabricated a homochiral MOF membrane on a nickel net. This in situ facile growth method has the advantages of cheap raw materials, simple operation and high thermal stability. The nickel net played a role as the only nickel source added to the reaction system and also as a substrate to support the membrane. As shown in **Figure** **37**, the nickel net substrate was placed horizontally in a Teflon‐lined autoclave and allowed to react with organic ligands in a solution of appropriate concentration. The crystals of Ni_2_(*L*‐asp)_2_(bipy) started to grow around the wires of the nickel net, and then continued to intergrow until a layer of film was formed, resulting in a thin and defect‐free membrane. This stable homochiral membrane was used to separate racemic diol mixtures in a pervaporation process under varied conditions and resulted in *ee* value of 32.5%.^[^ ^391^ ^]^

**Figure 38:**
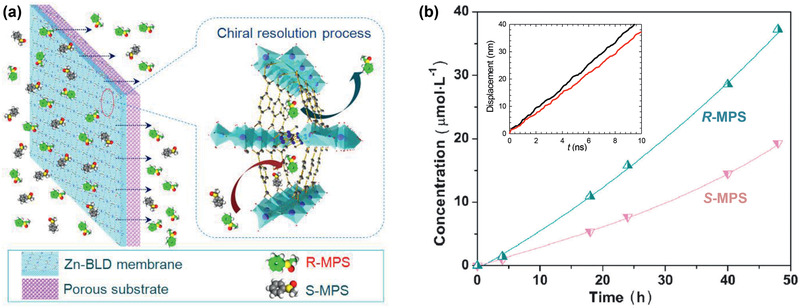
a) Homochiral MOF membrane for the separation of racemic MPS mixture. b) *R*‐/*S*‐MPS concentrations on the permeate side (the inset indicates simulation results). Reproduced with permission.^[^ ^397^ ^]^ Copyright 2012, Royal Society of Chemistry.

By integrating experimental and simulation methods, Jin and co‐workers examined the separation of racemic *R*‐/*S*‐methyl phenyl sulfoxide (MPS) by a membrane produced from homochiral Zn_2_(bdc)(*L*‐lac)(dmf). As illustrated in **Figure** **38**a, the membrane was prepared on ZnO support by a reactive seeding method. The porous support acted as inorganic source reacting with chiral ligand and an organic connector to grow a seeding layer for the secondary growth of MOF membrane. Thus, a strong adhesive and uniform seeding layer was achieved to aid in the preparation of defect free MOF membrane. Experimentally, *R*‐MPS was found to diffuse through the membrane relatively faster than *S*‐MPS. As shown in Figure 38b, the simulation predicted same transport order. To provide microscopic details into the chiral separation, the interaction energies of *R*‐/S‐MPS with the MOF were estimated. *S*‐MPS was found to possess a stronger interaction with the MOF, thus exhibiting slow transport. This study potentially establishes a new, sustainable and highly efficient technique for chiral separation.^[^ ^397^ ^]^

It should be noted that Snurr and co‐workers simulated chiral separation in MOFs; however, their simulations were not explicitly in a liquid phase. Specifically, they examined the adsorption of chiral hydrocarbons including 1,3‐dimethylallene, 1,2‐dimethylcyclobutane and 1,2‐dimethylcyclopropane in homochiral MOFs consisting of cadmium centers and BINOL‐type linkers. The enantioselectivity was attributed to small zig‐zag channels rather than large ones in these MOFs. Therefore, proper fitting of an adsorbate inside a chiral framework was revealed to be crucial for enantioselectivity.^[^ ^398^ ^]^ They further computationally screened eight homochiral MOFs to separate 19 chiral compounds by enantioselective adsorption. It was found that high *ee* values could be achieved when the size of chiral adsorbate matching well with channel size. Even with a good match, however, there was no guarantee for enantioseparation due to oscillating nature.^[^ ^399^ ^]^ Navarro‐Sánchez et al. reported a simulation study on the use of a chiral Cu‐MOF based on tripeptide Gly‐*L*‐His‐Gly (GHG) for the enantioselective separation of metamphetamine and ephedrine. It was suggested that chiral recognition was linked to preferential binding of one enantiomer, as a result of either stronger or additional H‐bonds with the framework; meanwhile, experimental solid‐phase extraction of a racemic mixture by using Cu(GHG) as an extractive phase allowed isolating > 50% of (+)‐ephedrine enantiomer as a target compound within only 4 min. This represents the first example of a MOF capable of separating chiral polar drugs.^[^ ^400^ ^]^

**Figure 39:**
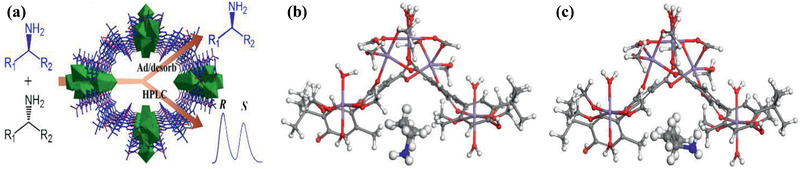
a) Separation of racemic amines in HPLC. b,c) Binding sites of (*R*)‐2‐butylamine and (*S*)‐2‐butylamine in the host. Reproduced with permission.^[^ ^401^ ^]^ Copyright 2014, Springer Nature.

Cui and co‐workers synthesized two homochiral robust 1,1′‐biphenol‐based MOFs containing 1D nanosized channels decorated with chiral dihydroxyl or dimethoxy groups. The MOFs exhibited high thermal stability and framework robustness as well as permanent porosity. Particularly, the framework containing chiral dihydroxyl auxiliaries could be utilized as an adsorbent for the separation of racemic aromatic and aliphatic amines with up to 98.3% *ee*, and as a chiral stationary phase in HPLC to separate benzoylated aromatic amines (**Figure** **39**). Experimental and simulation results revealed that the intrinsic chiral recognition and separation were attributed to the different orientations and binding energies of two enantiomers within the microenvironment of the host.^[^ ^401^ ^]^

**Figure 40:**
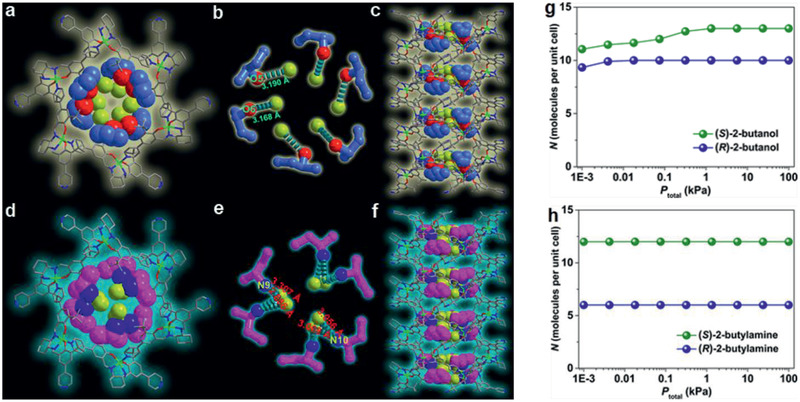
X‐ray crystal structures of a) 6(*S*)‐2‐butanol·6H_2_O, b) hexamer of six (*S*)‐2‐butanol, d) 6(*S*)‐2‐butylamine·9H_2_O, e) (*S*)‐2‐butylamine with water molecules in a slipped parallel conformation in the chiral pocket. The side view of c) 6(*S*)‐2‐butanol·6H_2_O and f) 6(*S*)‐2‐butylamine·9H_2_O in the chiral pocket. Adsorption isotherms of racemic mixtures: g) (*S*)‐/(*R*)‐2‐butanol h) (*S*)‐/(*R*)‐2‐butylamine from simulation. Reproduced with permission.^[^ ^351^ ^]^ Copyright 2017, American Chemical Society.

Furthermore, Cui and co‐workers reported three chiral fluorescent Zn_6_L_6_ metallacycles from pyridyl‐functionalized Zn(salalen) and Zn(salen) complexes, each with a nanoscale hydrophobic cavity decorated by six, three, and zero chiral NH functionalities. The metallacycles were observed to pack into 3D supramolecular porous frameworks shown in **Figure** **40**, on which chiral drug analytes could be detected immediately upon mixing without any extra sample treatment. This was the first example of enantioselective sensing of *l*‐dopa and *d*‐penicillamine chiral drugs with high quenching constants and selectivity factors. The binding affinity and enantioselectivity of the metallacycles towards *α*‐hydroxycarboxylic acids, amino acids, small molecule pharamaceuticals (*L*‐dopa, *D*‐penicillamine), and chiral amines were found to have a consistent relation with the number of chiral NH moieties in the cyclic structures. Based on single‐crystal X‐ray diffraction, molecular simulation and DFT calculation, the chiral recognition and discrimination were attributed to the specific binding of enantiomers in the chiral pockets of the metallacycles. This study suggests that manipulation of chiral NH functionalities in metallacycles would control the enantiorecognition of biomolecular complexes and facilitate the design of more effective supramolecular assemblies for enantioselective separation.^[^ ^351^ ^]^

Possibility of making homochiral porous materials is a unique feature of MOFs. Chiral MOFs can be synthesized from one of the following approaches: using enantiopure linkers or chiral templates to impart chiral structures, or postsynthetic modification of linkers or metal sites. The textural properties of chiral MOFs produced by the two de novo methods are still maintained, whereas the postsynthetic modification may alter the structures. The enantioselective capability of chiral MOFs mainly comes from the interaction and orientation of guest molecules. Several enantiomers such as alcohols and polar drugs were tested in the above discussed studies. At present, chiral MOFs are mostly tested in adsorption‐based separation, which however is associated with small separation capacity and high regeneration cost. As an alternative, chiral MOF membranes have emerged in pervaporation process for chiral separation. It is worthwhile to note that porous organic frameworks with chiral structures may be synthesized, but they are usually flexible, amorphous and not well‐suited for chiral separation. On the other hand, it is challenging to synthesize porous inorganic frameworks with chiral functionality. These shortcomings in both organic and inorganic frameworks are potentially addressed by using MOFs for chiral separation.

### 4.2 Drug Delivery

High‐efficacy drug delivery in a safe manner is of paramount importance for therapeutic treatment. Materials with ordered porous structures allow fine control of drug loading and release. The loading capacity and release of drug in a porous host are governed by several factors such as pore size, surface area and host affinity. In conventional porous materials (e.g., inorganic silica and carbon), the loading capacity is usually not sufficiently high and drug is difficult to be specifically released. With the readily tunable and well‐defined structures, MOFs have been increasingly investigated for in vitro and in vivo drug delivery as summarized in **Table** **7**.^[^ ^402^ ^]^

**Table 7:** Drug delivery

Drug	MOF	Ref.
Ibuprofen	MIL‐101	^[^ ^403^ ^]^
Ibuprofen	MIL‐53	^[^ ^404^ ^]^
Nicotinic acid	BioMIL‐1	^[^ ^405^ ^]^
Busulfan, azidothymidine triphosphate, cidofovir, doxorubicin, ibuprofen, caffeine, urea, benzophenone	MIL‐53, MIL‐88A, MIL‐89, MIL‐100, MIL‐101‐NH_2_	^[^ ^406^ ^]^
Busulfan	MIL‐53, MIL‐88A, MIL‐89, MIL‐100	^[^ ^407^ ^]^
Caffeine	Functionalized MIL‐88B	^[^ ^408^ ^]^
Caffeine	NH_2_‐MIL‐88B(Fe)	^[^ ^409^ ^]^
Caffeine, ibuprofen	Functionalized UiO‐66	^[^ ^410^ ^]^
Camptothecin, doxorubicin, daunomycin, SN‐38	Zn(bix)	^[^ ^411^ ^]^
Nimesulide	HKUST‐1/Fe_3_O_4_ composite	^[^ ^412^ ^]^
Deferiprone	[Zn_3_(bdc)_2_(dfp)_2_]‐2DMF, [Zn_3_(bdc)_2(_dfp_)2_(H_2_O)_2_]	^[^ ^413^ ^]^
Indomethacin	MIL‐101	^[^ ^414^ ^]^
Fluorescein, camptothecin	ZIF‐8 nanosphere	^[^ ^415^ ^]^
Alendronate	UiO‐66 NPs	^[^ ^416^ ^]^
Doxorubicin	MIL‐100(Fe)	^[^ ^417^ ^]^
Doxorubicin	UiO‐68	^[^ ^418^ ^]^
Disuccinatocisplatin	NCP‐1	^[^ ^419^ ^]^
Cisplatin	MIL‐101	^[^ ^420^ ^]^
Bisphosphonates	Ca‐Pam, Ca‐Zol	^[^ ^421^ ^]^
1‐amino‐adamantane, aspirin, nicotine	[Co_8_L_12_]^16+^	^[^ ^422^ ^]^
Gallic acid	Mg(H_2_gal)	^[^ ^423^ ^]^
Rhodamine 6G and doxorubicin hydrochloride	CP5‐capped UMCM‐1‐NH–Py	^[^ ^424^ ^]^
Resorufin, caffeine, and procainamide	UiO‐66‐PNIPAM	^[^ ^425^ ^]^
Procainamide	Bio‐MOF‐1	^[^ ^426^ ^]^
Fluorescein dye	Lipid bilayer‐coated MIL‐100/MIL‐101	^[^ ^427^ ^]^
Calcein dye	Amorphous UiO‐66	^[^ ^428^ ^]^
Ibuprofen	Magnetic HKUST‐1	^[^ ^429^ ^]^
Ibuprofen, captopril, caffeine	PI‐COFs	^[^ ^430^ ^]^
Ibuprofen	Zn(ibu)_2_(SCS), ZnCl(ibu)(SCS), Zn(ibu)_2_(H_2_O)_2_, ZnCl_2_(SCS)	^[^ ^431^ ^]^
Ibuprofen	PAF‐6	^[^ ^432^ ^]^
5‐fluorouracil	Cu(pi) nanocage	^[^ ^433^ ^]^
5‐fluorouracil	C‐dots@ZIF‐8 NPs	^[^ ^434^ ^]^
5‐fluorouracil	MOP‐15	^[^ ^435^ ^]^
5‐fluorouracil	BIT‐1, BIT‐2, BIT‐3	^[^ ^436^ ^]^
5‐fluorouracil	IFMC‐1	^[^ ^437^ ^]^
5‐fluorouracil	NTU‐Z11, GDMU	^[^ ^438^ ^]^ (exp./sim.)
5‐fluorouracil	[(CH_3_)_2_NH_2_], [Sm_3_(L1)_2_(HCOO)_2_(DMF)_2_(H_2_O)]·2DMF·18H_2_O, [Cu_2_(L2)(H_2_O)_2_]·2.22DMA, [Zn_2_(L1)(DMA)]·1.75DMA	^[^ ^439^ ^]^ (exp./sim.)
5‐fluorouracil	[Zn_8_(O)_2_(CDDB)_6_(DMF)_4_(H_2_O)	^[^ ^440^ ^]^
5‐fluorouracil	Zn(TATAT)_2/3_	^[^ ^441^ ^]^
5‐fluorouracil	ZIF‐8	^[^ ^442^ ^]^
5‐fluorouracil	CP5‐capped UiO‐66‐NH‐A	^[^ ^443^ ^]^
Rhodamine B, methyl red, and salicylic acid	MOF/CW microcapsules	^[^ ^444^ ^]^
Allyl isothiocyanate food preservative	Zn_3_(bpdc)_3_(apy)	^[^ ^445^ ^]^
Curcumin	Zr‐TCPE NCP	^[^ ^446^ ^]^
Caffeine, Ibuprofen	PVA‐MIL‐100, GEL‐MIL‐100	^[^ ^447^ ^]^
Calcein, *α*‐cyano‐4‐hydroxycinnamic	Amorphous Zr‐MOFs	^[^ ^448^ ^]^
5‐fluorouracil	TpASH, TpASH‐FA,TpASH‐APTES	^[^ ^449^ ^]^
Gallic acid	Mg(H_2_gal), MIL‐155, and MIL‐156	^[^ ^450^ ^]^
Alphacyano‐4‐hydroxycinnamic acid	NU‐1000 and NU‐901	^[^ ^451^ ^]^
Ibuprofen	CD‐MOF	^[^ ^452^ ^]^
Calcein	Exosome‐coated MIL‐88A	^[^ ^453^ ^]^
Ibuprofen, rhodamine B, and doxorubicin	MIL‐101(Fe)‐(NH_2_)x, MIL‐101(Fe)‐(C_4_H_4_)*_x_*, and MIL‐101(Fe)‐(C_4_H_4_)*_x_*(NH_2_)_1−_ *_x_*	^[^ ^454^ ^]^
Ibuprofen and lansoprazole	CD‐MOF/PAA composites	^[^ ^455^ ^]^
Gemcitabine monophosphate	MIL‐100(Fe) NPs	^[^ ^456^ ^]^
Fluorouracil and niacin	Hofmann MOFs	^[^ ^457^ ^]^
Doxorubicin or Rhodamine 6G	VEGF aptamer functionalized NMOF	^[^ ^458^ ^]^
Single‐stranded DNA	Ni‐IRMOF‐74‐II to ‐V	^[^ ^459^ ^]^
Insulin	NU‐1000	^[^ ^460^ ^]^
Doxorubicin	PEG‐CCM@APTES‐COF‐1	^[^ ^461^ ^]^
Dichloroacetate	UiO‐66‐FA	^[^ ^462^ ^]^
Doxorubicin	Magnetic micromotors ZIF‐67/Fe_3_O_4_	^[^ ^463^ ^]^
Doxorubicin	ZrMOF‐PEG‐TPP nanocubes	^[^ ^464^ ^]^
Insulin	GOx/ZIF‐8	^[^ ^465^ ^]^
In vivo toxicity	MIL‐88A, MIL‐88B_4CH3, MIL‐100	^[^ ^466^ ^]^
In vivo toxicity	MIL‐100(Fe, Al, Cr)	^[^ ^467^ ^]^
Ibuprofen	MIL‐101, UMCM‐1	^[^ ^468^ ^]^ (sim.)
Ibuprofen, methylene blue, amoxicillin and gentamicin	[Zn(BDC)(H_2_O)_2_]_n_	^[^ ^469^ ^]^ (sim.)
Caffeine	MIL‐100, UiO‐66, MIL‐53, MIL‐127	^[^ ^470^ ^]^ (sim.)
Ibuprofen	MIL‐53, CD‐MOF‐1, MOF‐74, MIL‐100, MIL‐101, BioMOF‐100	^[^ ^471^ ^]^ (sim.)
Tamoxifen	IRMOF‐14, IRMOF‐16	^[^ ^472^ ^]^ (sim.)
Caffeine, urea, niacin, and glycine	HKUST‐1	^[^ ^473^ ^]^ (sim.)
Ibuprofen, caffeine and urea	24 MOFs	^[^ ^474^ ^]^ (sim.)
Methotrexate and 5‐fluorouracil	Isoreticular structures of MOF‐74	^[^ ^475^ ^]^ (sim.)
Gemcitabine	IRMOF‐74‐III, OH‐IRMOF‐74‐III	^[^ ^476^ ^]^ (sim.)
6‐mercaptopurine	Peptide‐based MOF	^[^ ^477^ ^]^ (sim.)

**Figure 41:**
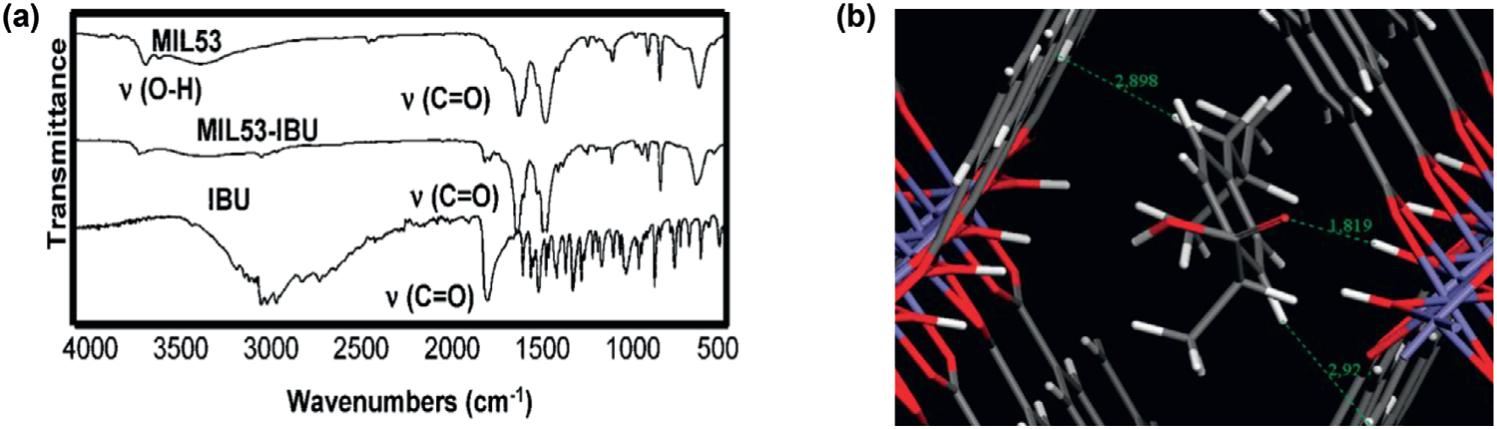
a) Fourier transform infrared spectroscopy of MIL‐53 and ibuprofen. b) Optimized arrangement of ibuprofen in MIL‐53. Fe: blue, O: red, C: gray, H: white. Reproduced with permission.^[^ ^404^ ^]^ Copyright 2008, American Chemical Society.

Férey and co‐workers first explored the potential of MOFs for drug loading and controlled delivery. Specifically, anti‐inflammatory and analgesic ibuprofen was loaded from hexane solution by dehydrated MIL‐100 and MIL‐101. Due to their different structural features and selective occupation of cages, MIL‐100 and MIL‐101 exhibited drastically different loading amounts of ibuprofen (0.35 g g^−1^ MIL‐100 and 1.4 g g^−1^ MIL‐101). The loading in MIL‐101 was almost four and nine times higher than in mesoporous silica and zeolites, respectively. For delivery, three different stages were observed corresponding to the release of ibuprofen from different cages in MIL‐100 and MIL‐101. This behavior is completely different from the continuous release profile observed in MCM‐41, and demonstrates the important effects of pore size, surface area and structural properties on drug loading and release in MOFs.^[^ ^403^ ^]^ Ibuprofen delivery was further examined in flexible MIL‐53(Cr, Fe) that could reversibly modulate pore size to the dimension of drug to optimize drug‐host interactions. Around 20 wt% of ibuprofen was loaded in MIL‐53(Cr) and MIL‐53(Fe), implying independence of metal type in MIL‐53. As depicted in **Figure** **41**a, the *ν*(C=O) band of carboxylic group in ibuprofen was observed to shift from 1695 to 1725 cm^−1^ upon loading, meanwhile, the vibrational band (O–H) of inorganic chain in MIL‐53 varied from 3656 to 3639 cm^−1^. The shift was attributed to the formation of hydrogen bond between carboxylic group in ibuprofen and hydroxyl group in MIL‐53 (Figure 41b) as confirmed by DFT calculations.^[^ ^404^ ^]^ Subsequently, they functionalized non‐toxic Fe‐based MOFs (MIL‐53, MIL‐89, MIL‐89A, MIL‐100, and MIL‐101_NH_2_) with engineered cores and surfaces as superior nanocarriers for efficient controlled delivery of challenging antiumoural and retroviral drugs (e.g., busulfan, azidothymidine triphosphate, doxorubicin, and cidofovir). These MOFs exhibited unprecedented loadings for a large number drugs ranging from hydrophilic, hydrophobic to amphiphilic, thus potential useful in therapeutics, diagnostics and personalized patient treatment.^[^ ^406^ ^]^

Later, Serre, Maurin and co‐workers experimentally measured the loading capacities of caffeine in a series of MIL‐88B functionalized by polar and apolar groups (–H, –Br, –F, –CH_3_, –NH_2_, –NO_2_, and –OH). The highest amount of loading was observed with hydrophilic –NH_2_ and –OH, while the lowest was with hydrophobic –CH_3_. Due to hydrogen bonding or electrostatic interaction, –NH_2_ and –OH were shown to possess strong affinity for drug. The effect of functionalization was found being in accord with previous studies in amine functionalized MCM‐41 and revealed the dominant role of hydrophilic groups in drug loading.^[^ ^408^ ^]^ Similar measurements were also conducted for caffeine and ibuprofen in functionalized UiO‐66, with loadings dependent on the nature of functional groups. While hydrophobic –CH_3_ was observed to be preferential for caffeine loading, a different dependence was seen for ibuprofen. Moreover, the solvent used to dissolve drug was found to be crucial in drug loading, as attributed to the competitive adsorption between drug and solvent. Based on these experiments, quantitative structure‐activity relationships were proposed to identify the most relevant chemical and structural features of MOFs that would significantly affect drug loading and predict drug loading in other MOFs.^[^ ^410^ ^]^

Lin and co‐workers reported a novel and general strategy using nanoscale degradable NCP‐1 for the delivery of Pt‐based drugs into cancer cells. NCP‐1 was constructed from Tb^3+^ ions and *c*,*c*,*t*‐(diamminedichlorodisuccinato) Pt bridging ligands. Although the Pt‐based NCP‐1 was stable and dispersible in most organic solvents, particle formation was reversible under excessive water. To prevent rapid dissolution and effectively control release, NCP‐1 was coated with amorphous silica shell and its anticancer efficacy was demonstrated in vitro on HT‐29 cells.^[^ ^419^ ^]^ The same strategy was incorporated to post‐synthetically modify iron‐carboxylate nanoscale MIL‐101 for delivery of anticancer drugs, as well as high payloads of imaging contrast agents.^[^ ^420^ ^]^ Recently, they also synthesized nanoscale Ca‐Pam and Ca‐Zol by linking Ca^2+^ with N‐containing bisphosphonates, pamidronate and zoledronate. To slow down the dissolution in biological media, the nanoparticles were coated with single lipid bilayer consisting of 1:1 DOTAP/DOPE (DOTAP = dioleoyl trimethylammonium propane, DOPE = dioleoyl *L*‐*α*‐phosphatidylethanolamine). Compared with as‐synthesized particles and free bisphosphonates, the nanoparticles showed superior anti‐tumor efficacy in vitro against human lung and pancreatic cancer cells.^[^ ^421^ ^]^

**Figure 42:**
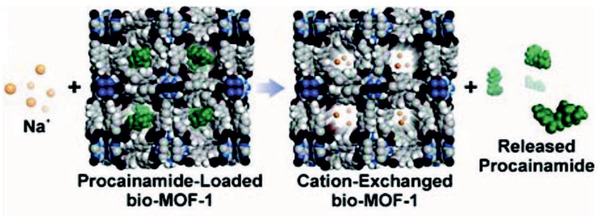
Cation‐triggered procainamide release from bio‐MOF‐1. Reproduced with permission.^[^ ^426^ ^]^ Copyright 2009, American Chemical Society.

Using adenine (a purine nucleobase) as biomolecular ligand, Rosi and co‐workers synthesized permanently porous bio‐MOF‐1. With an anionic framework, bio‐MOF‐1 was able to maintain its crystallinity for weeks in water, various organic solvents and biological buffers. A cationic drug, procainamide, was introduced into the pores of bio‐MOF‐1 by exchanging with dimethylammonium cations. Such an ion exchange did not affect the crystalline integrity. Approximately, 2.5 procainamide molecules per formula unit were loaded in bio‐MOF‐1. As depicted in **Figure** **42**, the loaded procainamide could be triggered to release steadily by exogenous cations in a phosphate buffered saline (PBS). This study represents the first example to demonstrate the potential biomedical application of a MOF with biomolecular building blocks.^[^ ^426^ ^]^

Based on the nucleophilic substitution of cyanuric chloride, Zhao et al. designed and synthesized a 2D porous organic framework (PAF‐6) with tubular channels of 11.5 × 12.6 Å^2^ in diameter. No cytotoxicity was found in PAF‐6 and in vitro delivery of ibuprofen was tested. PAF‐6 could load 0.35 g g^−1^ and a large initial burst release was observed within 10 h.^[^ ^432^ ^]^ Sun et al. prepared a pair of chiral non‐interpenetrated MOFs with two types of nanoscale cages (hexagonal prism‐shaped and trigonal prismatic). The frameworks were anionic and counterbalanced by (CH_3_)_2_NH_2_^+^ cations. Adsorption of anti‐cancer drug 5‐fluorouracil (5‐FU) in these MOFs from methanol solutions was measured and the loading was about 0.5 g g^−1^. No burst effect was observed in the release of 5‐FU in a PBS buffer.^[^ ^441^ ^]^ They further evaluated ZIF‐8 as a drug delivery vehicle for 5‐FU. A loading of 0.66 g g^−1^ was determined as the highest reported loading capacity of 5‐FU. Interestingly, the release of 5‐FU was pH‐sensitive. In a PBS buffer (pH = 7.4), the release was slow with about 17% of 5‐FU released within 1 h. In an acetate buffer (pH = 5), however, a remarkably faster release was observed with over 45% of 5‐FU released within 1 h. This suggests that the drug release could be accelerated upon approaching cancer cells.^[^ ^442^ ^]^

**Figure 43:**
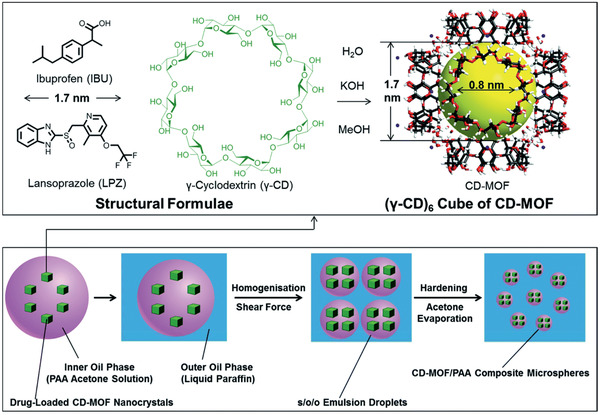
(Bottom) Preparation of CD‐MOF/PAA composite microspheres. (Top) Structural formulae for ibuprofen, lansoprazole and *γ*‐cyclodextrin and CD‐MOF. Reproduced with permission.^[^ ^455^ ^]^ Copyright 2017, Royal Society of Chemistry.

MOF/polymer composites are emerging class of carriers for sustained drug delivery and reduced cell toxicity. Li et al. prepared low toxic composite microspheres of *γ*‐cyclodextrin (*γ*‐CD) MOFs (CD‐MOFs) and polyacrylic acid (PAA) by a solid in oil‐in‐oil (s/o/o) emulsifying solvent evaporation method. The drugs (ibuprofen and lansoprazole) were loaded in the CD‐MOF/PAA composites by co‐crystallization, more effectively than impregnation and without any MOF crystal degradation. It was observed that good dispersion of drug‐loaded CD‐MOF nanocrystals inside PAA matrices resulted in microspheres with better sphericities than those prepared using the pure drug or drug‐*γ*‐CD complexes (**Figure** **43**). Moreover, the homogeneous distribution of drug molecules in these nanocrystals contributed towards sustained almost linear drug release over a prolonged period of time. Strikingly different from a very fast burst and uncontrolled release from drug‐*γ*‐CD complexes, no burst release of ibuprofen and lansoprazole was observed from the CD‐MOF/PAA composite microspheres. It suggests that an even distribution of drug as well as strong electrostatic and hydrophobic carrier‐drug interactions inside the CD‐MOF.^[^ ^455^ ^]^

**Figure 44:**
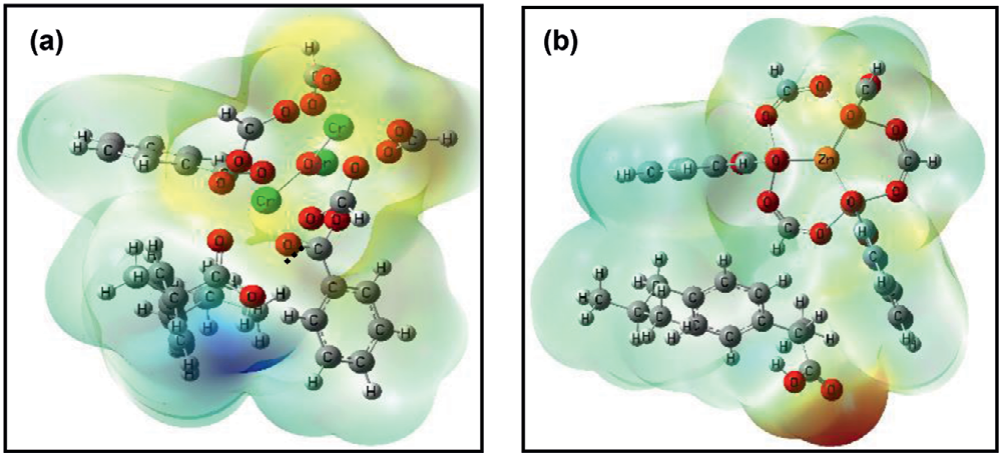
Optimized conformations of ibuprofen in a) MIL‐101 and b) UMCM‐1. The dotted line: a coordination bond between ibuprofen and the Cr site in MIL‐101. Reproduced with permission.^[^ ^468^ ^]^ Copyright 2009, American Chemical Society.

Along with the above discussed experimental efforts, computational tools have been implemented to investigate drug loading in MOFs. Babarao and Jiang conducted molecular simulations to understand the microscopic properties of ibuprofen in MIL‐101 and UMCM‐1, respectively. The loading in MIL‐101 was predicted to be 1.11 g g^−1^, agreeing well with experimentally determined 1.37 g g^−1^. In both MIL‐101 and UMCM‐1, ibuprofen loading was about four times higher than in MCM‐41. The substantially high loadings in these two MOFs suggest that they might be useful for drug delivery. The mobility of ibuprofen in MIL‐101 was found to be smaller than in UMCM‐1, attributed to the formation of coordination bond between ibuprofen and MIL‐101 as shown in **Figure** **44**. However, no such bond was observed with UMCM‐1 due to the absence of unsaturated metal sites. This study provided the energetic and dynamic insights into ibuprofen in two mesoscopic MOFs. It should be noted that the experimental study of ibuprofen loading/delivery in MIL‐101 was conducted in hexane, which was not included in simulation. The inclusion of solvent might affect MOF‐drug interaction and accessibility.^[^ ^468^ ^]^ Using molecular docking, Rodrigues et al. predicted the binding conformations and affinities of ibuprofen, amoxicillin, gentamicin in [Zn(BDC)(H_2_O)_2_]_n_ under different pH conditions. Based on the lowest energy conformer, ibuprofen was found to interact with the framework through electrostatic and *π*–*π* interactions; amoxicillin was bound mainly onto Zn^2+^; gentamicin was not bound due to strong electrostatic repulsion and large molecular size. These predictions agreed with experimental measurements from X‐ray powder diffraction, differential thermal analysis and UV–vis spectroscopy. The approach is useful to distinguish good and poor drug candidates for loading into MOFs.^[^ ^469^ ^]^

**Figure 45:**
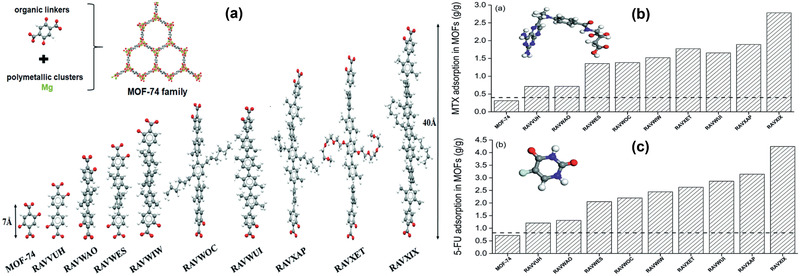
a) Organic linkers in MOF‐74 structures. b) MTX and c) 5‐FU loadings in MOF‐74. Reproduced with permission.^[^ ^475^ ^]^ Copyright 2017, Royal Society of Chemistry.

To identify suitable MOFs for ibuprofen loading, Snurr and co‐workers performed molecular simulations to analyse ibuprofen adsorption in a series of bio‐compatible MOFs with different textural properties (MIL‐53, MIL‐100, MIL‐101, MOF‐74, CD‐MOF‐1, and bioMOF‐100). The loading capacities were found to correlate well with pore volume. With the highest pore volume among these six MOFs, bioMOF‐100 exhibited the highest loading capacity (1975 mg g^−1^), which is almost 6–13 times higher than in mesoporous silicas and dealuminated faujasites. The potential energy of an ibuprofen inside a host material was able to explain both short and controlled delivery time observed in experiments.^[^ ^471^ ^]^ Alternatively, DFT calculations were used to investigate the interaction energies of biologically important organic molecules such as caffeine, urea, niacin, and glycine in HKUST‐1. Two types of binding modes were analyzed, one being adsorption on Cu sites and the other adsorption in pores. The binding energies between the nitrogen or oxygen atoms of the molecule with the Cu sites were predicted much stronger than hydrogen bonding with the carboxylate groups in HKUST‐1.^[^ ^473^ ^]^ Erucar and Keskin examined the storage and delivery of two anticancer drug molecules, 5‐FU and methotrexate (MTX), in 10 different MOF‐74 structures. The 10 MOFs were built by increasing the number of phenylene rings from one (MOF‐74) to ten (RAVXIX) as shown in **Figure** **45**a. The predicted loadings of 5‐FU and MTX as in Figure 45b,c were found to rise with the number of phenylene rings. The higher loading capacities than other porous materials and slow diffusion of drug molecules suggest that MOF‐74s have strong potential as multi‐drug carrier systems.^[^ ^475^ ^]^

**Figure 46:**
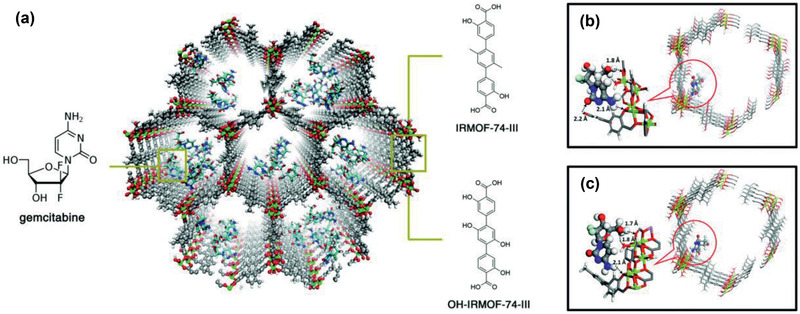
a) 32 wt% GEM loading. Optimized geometries of GEM in b) OH‐IRMOF‐74‐III and c) IRMOF‐74‐III. MOF: C, gray; Mg, green; O, red; H, white. GEM: C, cyan; O, red; N, blue; H, white; F, light blue. Reproduced with permission.^[^ ^476^ ^]^ Copyright 2017, Royal Society of Chemistry.

Froudakis and co‐workers investigated the adsorption and diffusion of anti‐cancer drug gemcitabine (GEM) in IRMOF‐74‐III and functionalized OH‐IRMOF‐74‐III using a multiscale computational approach (**Figure** **46**). The GEM‐MOF interaction energies from DFT calculations in both MOFs were predicted to be suitable for drug loading and control release. The calculated maximum loadings of GEM in both MOFs using Monte Carlo simulations were nearly same and three times higher than in lipid‐coated mesoporous silica nanoparticles. The mobility of GEM was found slightly lower in IRMOF‐74‐III than in OH‐IRMOF‐74‐III due to the formation of drug aggregations. Overall, the mobility was slow and of the same magnitude in both MOFs, indicating the slow release of GEM. This work highlights the importance studying microscopic behavior of a drug in porous materials for the better control of drug loading and release.^[^ ^476^ ^]^ Recently, a peptide‐based MOF (MPF) was examined by Shahabi and Raissi for the delivery of 6‐mercaptopurine (6‐MP). From molecular dynamics simulations, the interaction strength between 6‐MP and MPF was found to decrease under an electric field. Based on radial distribution function patterns, the probability to find 6‐MP molecules near MPF surface was lower at a higher field strength. With increasing electric field strength, 6‐MP molecules around MPF exhibited higher dynamic movement. The simulation study suggests that the applied electric field could act as a trigger on drug liberation in a porous nanomaterial.^[^ ^477^ ^]^

To be useful in drug delivery, MOFs should be biocompatible, biodegradable, and non‐toxic. In this regard, iron‐based MILs and MOFs with biomolecular building blocks have been tested extensively. Important factors considered include MOF‐drug interaction, loading amount, delivery time, and mechanism. Along with the metal‐ligand built‐in features of MOFs, functionalization may be used to tune drug delivery. The in vitro studies based on bulk MOFs demonstrate the promise of MOFs as drug delivery vehicles; however, direct use in this form is not well‐suited for systemic circulation. Several other forms of MOFs such as microspheres and core–shells have been investigated to increase biocompatibility and slow dissolution after loading; nanoscale MOFs appear to be more suitable for in vivo application. Thus, downsizing to nanoregime while maintaining the exceptional properties of bulk form is expected to further advance MOFs for drug delivery.

### 4.3 Biomolecule Encapsulation and Separation

In various industrial relevant and in vitro applications, it is important to encapsulate and separate biomolecules. Biomolecule encapsulation on a host matrix improves the stability and applicability for enzymatic catalysis, bioimaging and biospecimen preservation. Porous hosts such as mesoporous silica have been examined; due to the lack of specific interactions, however, the encapsulated biomolecules may leach during application and cause poor long‐term usability. Solid phase extraction (SPE) is commonly used in peptidomic/proteomic analysis and biosample preparation. Functional porous materials with features of different selection mechanisms for biomolecule separation are suitable for SPE. In the past a few years, MOFs have been explored as a platform for biomolecule encapsulation and separation, as listed in **Table** **8**, towards the development of biomolecule‐MOF composites, peptidomic technology, biosample purification, etc.

**Table 8:** Biomolecule encapsulation and separation

Biomolecule	MOF	Ref.
**Biomolecule encapsulation**		
Microperoxidase‐11	Tb‐mesoMOF	^[^ ^478^ ^]^
Hem protein cytochrome *c*	Tb‐mesoMOF	^[^ ^479^ ^]^
GFPs, RFPs	Hairy Cu‐MOF	^[^ ^480^ ^]^
HeLa (human cervical cancer) cells	DNA‐UiO66 conjugate	^[^ ^481^ ^]^
Cytochrome c	MN_3_(NDC)_3_DMF_4_	^[^ ^482^ ^]^
Cytochrome c	ZIF‐8	^[^ ^483^ ^]^
3D DNA	M_6_L_4_ sphere framework	^[^ ^484^ ^]^
Ferritin, glucose oxidase	HKUST‐1 film	^[^ ^485^ ^]^
HRP, cytochrome c, MP‐11	PCN‐333(Al)	^[^ ^486^ ^]^
Myoglobin, green fluorescent protein	IRMOF‐74	^[^ ^487^ ^]^
Catalase	ZIF‐90	^[^ ^488^ ^]^
DNA, proteins, enzymes	ZIF‐8, HKUST‐1,Eu‐BDC, Tb‐BDC, MIL‐88A	^[^ ^489^ ^]^
Proline, proline‐glycine dipeptide, polypeptides	Al‐MIL‐101‐NH2, In‐MIL‐68‐NH_2_, and Zr‐UiO‐66‐NH_2_	^[^ ^490^ ^]^
Glucose oxidase, horseradish peroxidase	ZIF‐8	^[^ ^491^ ^]^
Urease	ZIF‐8	^[^ ^492^ ^]^
Phospholipid bilayers	Zr‐NMOFs	^[^ ^493^ ^]^
Catalase	ZIF‐90 and ZIF‐8	^[^ ^494^ ^]^
*α*‐chymotrypsin	Cu(bpy)2(OTf)_2_	^[^ ^495^ ^]^
Circulating tumor cells	ZnMOF‐COOH	^[^ ^496^ ^]^
Diphenylalanine	Cu_2_L_2_ted	^[^ ^497^ ^]^
Diphenylalanine	N_3_‐bio‐MOF‐100	^[^ ^498^ ^]^
HIV DNA, thrombin	Cu(H_2_dtoa)	^[^ ^499^ ^]^
Cytochrome c peroxidase	CuBDC	^[^ ^496^ ^]^
Lipase PS	COF‐OMe, PCN‐128y	^[^ ^500^ ^]^
Bovine serum albumin	PVP coated ZIF‐8 NPs	^[^ ^501^ ^]^
Protein biomarkers in urine, serum, plasma, and blood	ZIF‐8	^[^ ^502^ ^]^
Fatty acids	MIL‐101 and ZIF‐8	^[^ ^503^ ^]^
Enzyme	ZIF‐8	^[^ ^504^ ^]^
**Biomolecule separation**		
Peptides, proteins	MIL‐53, MIL‐100, and MIL‐101	^[^ ^505^ ^]^
Phosphopeptides	Er_2_(PDA)(H_2_O)	^[^ ^506^ ^]^
Angiotensin II (low‐abundance peptides), MYO and BSA tryptic digests	Fe_3_O_4_@[Cu_3_(btc)_2_]	^[^ ^507^ ^]^
Endogenous peptides in human serum	Fe_3_O_4_@MIL‐100(Fe)	^[^ ^508^ ^]^
*β*‐casein and *α*‐casein	Fe_3_O_4_@PDA@Zr‐MOF	^[^ ^509^ ^]^
Glycopeptides	MIL‐101(Cr)‐NH_2_	^[^ ^510^ ^]^
Glycopeptides	cross‐linked CD‐MOFs (LCD‐MOFs)	^[^ ^511^ ^]^
Histidine‐rich proteins, hemoglobin from a protein mixture and human blood samples	Fe_3_O_4_@ZIF‐8	^[^ ^512^ ^]^
BSA tryptic digests	Fe_3_O_4_‐COOH@MIL‐101	^[^ ^513^ ^]^
Phosphopeptides	UiO‐66, UIO‐67	^[^ ^514^ ^]^
Phosphopeptides	Fe_3_O_4_@MIL‐100(Fe)	^[^ ^515^ ^]^
Phosphopeptides	Fe_3_O_4_@PDA@Er(btc)	^[^ ^516^ ^]^
Glycopeptides	MG@Zn‐MOFs	^[^ ^517^ ^]^
33 glycopeptides	MIL‐101(Cr)‐maltose	^[^ ^518^ ^]^
Angiotensin II (peptide) and BSA (protein)	MGMOF	^[^ ^519^ ^]^
Human serum digest peptides	Fe_3_O_4_/C@MIL‐100(Fe)	^[^ ^520^ ^]^
BSA tryptic digest	Fe_3_O_4_@PDA@ZIF‐8	^[^ ^521^ ^]^
Glycopeptides	MIL‐101(NH_2_)@Au‐Cys	^[^ ^522^ ^]^
N‐glycopeptides	MIL‐101(Cr)‐NH_2_@PAMAM	^[^ ^523^ ^]^
N‐linked glycopeptides	UiO‐66‐COOH	^[^ ^524^ ^]^
N‐linked glycopeptides	Fe_3_O_4_@PDA@Zr‐SO_3_H	^[^ ^525^ ^]^
Phosphopeptides of *β*‐casein	DZMOF	^[^ ^526^ ^]^
Phosphopeptides from tilapia eggs	Fe_3_O_4_/MIL‐101(Fe)	^[^ ^527^ ^]^
Phosphopeptides in a tryptic digest of *β*‐casein	MIL‐101(Cr)‐UR2	^[^ ^528^ ^]^
Glycopeptides from 125 glycoproteins	Fe_3_O_4_@Mg‐MOF‐74	^[^ ^529^ ^]^
Nucleic acids (DNA and RNA)	Co‐IRMOF‐74‐II, ‐III, and ‐IV	^[^ ^530^ ^]^
Arginine, phenylalanine, tryptophan	MIL‐101	^[^ ^128^ ^]^ (sim.)
b‐adrenoceptor	IRMOF‐74‐II and ‐III	^[^ ^531^ ^]^ (sim.)
Trp‐cage	IRMOF‐74 I, ‐V1, ‐V2, ‐V1‐HA, and ‐V1‐DGA	^[^ ^532^ ^]^ (sim.)

**Figure 47:**
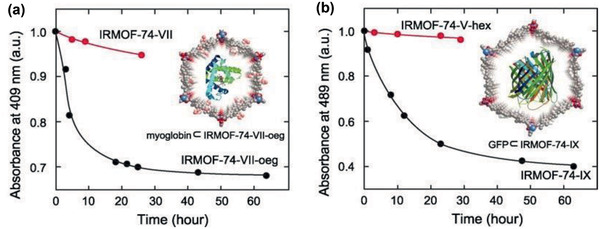
Encapsulation of a) myoglobin and b) green fluorescent protein in IRMOF‐74s. Reproduced with permission.^[^ ^487^ ^]^ Copyright 2012, American Association for the Advancement of Science.

Ma and co‐workers encapsulated a microenzyme microperoxidase‐11 (MP‐11) from HEPES (4‐(2‐hydroxyethyl)‐1‐piperazineethanesulfonic acid) buffer into a hydrophobic MOF known as Tb‐mesoMOF. MP‐11 has dimensions of 33 × 17 × 11 Å, smaller than the window sizes (13 and 17 Å) of Tb‐mesoMOF, thus it can enter into the mesoporous cages in Tb‐mesoMOF. Compared with the case immobilized in MCM‐41, MP‐11 in Tb‐mesoMOF achieved higher loading and exhibited higher enzymatic performance. This was attributed to the higher surface area and strong hydrophobic surface interaction between the encapsulated MP‐11 with Tb‐mesoMOF. The high catalytic activity along with the recyclability of MP‐11 suggested that Tb‐mesoMOF might serve as an interesting host material to immobilize enzymes for catalysis.^[^ ^478^ ^]^ Furthermore, they found that heme protein cytochrome *c* (Cyt *c*) could enter into the hydrophobic interior cavities of Tb‐mesoMOF. However, the molecular dimension of Cyt *c* (26 × 32 × 33 Å) is larger relative to the window size of Tb‐mesoMOF. From fluorescence measurement, it was revealed that Cyt *c* must undergo a significant conformational change during translocation upon contact with MOF surface.^[^ ^479^ ^]^ Yaghi and co‐workers reported a strategy to systematically expand the pore aperture of MOF‐74 by adding various numbers of phenylene rings. The produced homologous series of IRMOF‐74s possess non‐interpenetrating structures with hexagonal channels ranging from 14 to 98 Å and exhibit robust architectures with permanent porosity and high thermal stability (up to 300 °C). Compared with mesoporous silica, carbon, and zeolites, IRMOF‐74s have much higher surface areas, which is a desired characteristic for an encapsulation host to provide interaction environment. The encapsulation of large inorganic, organic and biological molecules into IRMOF‐74s was demonstrated. **Figure** **47**a,b illustrates that myoglobin (21 × 35 × 44 Å) and biomarker green fluorescent protein (34 × 34 × 45 Å) could be encapsulated in oligoethylene glycol‐functionalized IRMOF‐74‐VII and IRMOF‐74‐IX by tuning pore hydrophilicity and size, respectively. The crystal structures of IRMOF‐74s were fully maintained during encapsulation process.^[^ ^487^ ^]^

Through the encapsulation of diphenylalanine inside the nanoscale pores of Cu_2_L_2_ted, autonomous biomimetic motors were developed by Ikezoe et al. After dropping into an aqueous solution containing ethylenediaminetetraacetate, the peptide was triggered to release from Cu_2_L_2_ted and assemble at the MOF‐solution interface, creating a surface tension gradient and hence autonomous motion. The energy source of the motors was attributed to the free energy change of peptide emission, similar to biological motors working through dissipation of chemical energy. Due to the encapsulation and release of biomolecules in a highly ordered manner, the MOF‐based biochemical motors showed faster unidirectional velocities and higher kinetic energies compared with polymer gel‐based motors. This study opens the route towards reconfigurable molecular assembly, possibly evolving into smart micro‐devices.^[^ ^497^ ^]^ Liu et al. applied strain‐promoted click chemistry to modify the pore walls of mesoporous bio‐MOF‐100. Various functional groups were introduced by this method including succinimidyl ester bioconjugation moieties. The succinimide‐decorated bio‐MOF‐100 could anchor diphenylalanine onto its pore walls. This straightforward bioconjugation strategy could be applied to tether other peptides, enzymes, proteins, nucleic acids, etc.^[^ ^498^ ^]^ A 2D‐sheet MOF, Cu(H_2_dtoa), exhibiting a conjugated *π*‐electron system and a large surface area was synthesized by Zhu et al. The binding between a probe DNA and Cu(H_2_dtoa) due to hydrophobic and *π*‐stacking interactions quenched fluorescence emission, which could be restored by introducing targets. Based on this principle, HIV, DNA and thrombin were detected with high sensitivity and selectivity.^[^ ^499^ ^]^

**Figure 48:**
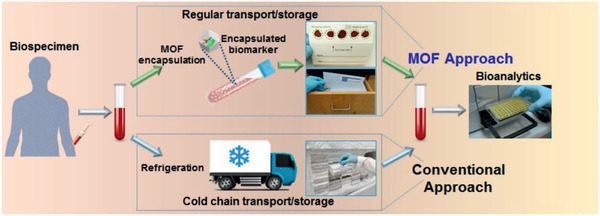
Using MOF encapsulation for biospecimen preservation. Reproduced with permission.^[^ ^502^ ^]^ Copyright 2018, American Chemical Society.

Wang et al. proposed a biospecimen preservation concept using MOFs to increase the thermal stability of protein biomarkers in biosamples under nonrefrigeration conditions. As depicted in **Figure** **48**, the concept was demonstrated with urinary NGAL (neutrophil gelatinase‐associated lipocalin) and serum/plasma CA‐125 biomarkers encapsulated in ZIF‐8. The protein biomarkers containing relevant biosamples were first mixed with ZIF‐8 precursors to be encapsulated during crystal formation and then the sample was dried on paper with dry spot sample collection method. It was observed that the MOF encapsulated protein biomarkers in urine, serum, plasma, and blood could be preserved at normal room temperature and 40 °C, with preservation efficacy comparable to refrigeration method at −20 °C. Thus, the route of biospecimen preservation by encapsulating into MOFs provides an alternative energy‐efficient, cost effective, and accessible approach to the existing cold chain system.^[^ ^502^ ^]^

Separation of peptides and proteins is important in the pharmaceutical industry. In this context, Yan and co‐workers reported the first example for MOF‐based enrichment of peptides with the simultaneous exclusion of proteins. Specifically, MIL‐53, MIL‐100, and MIL‐101 were introduced respectively into a standard peptide mixture, human plasma and urine sample; the enriched peptides were analyzed using mass spectrometry. The MILs were stable throughout the enrichment process and the peptides were unchanged after the enrichment. In the peptide mixture, MIL‐53, MIL‐100, and MIL‐101 gave an enrichment factor of 19, 64, and 98, respectively. Due to molecular sieving effect, the exclusion of proteins was observed in human plasma and urine samples.^[^ ^505^ ^]^ Similarly, a chemical stable MOF namely Er_2_(PDA)(H_2_O) was prepared by Messner et al. for the selective enrichment of phosphopeptides. A standard protein digest (*α*‐casein, *β*‐casein and ovalbumin), as well as digested egg‐white, was used to measure selectivity and efficiency. It was found that 14 phosphopeptides could be recovered from the peptide mixture and 4 from the digested egg‐white. To explain the high affinity, ab initio calculations were conducted to estimate the binding energies between the MOF and phosphopeptides. It was observed that with decreasing crystal size, the loading of phosphopeptide was improved because of the increased surface area and interaction sites.^[^ ^506^ ^]^ In the above studies of biosample separation, centrifugation at a high speed was required, which could lead to undesirable loss of low abundant peptides/proteins. Xiong et al. prepared magnetic MOF composites, Fe_3_O_4_@MIL‐100(Fe), to synergize the rich functional properties of MOFs with fast magnetic responsivity of magnetic materials. It was demonstrated that Fe_3_O_4_@MIL‐100(Fe) core/shell magnetic nanospheres were fast and effective in the enrichment of hydrophobic peptides from protein tryptic digest and achieved size selective enrichment of endogenous peptides from human serum with exclusion of large proteins.^[^ ^508^ ^]^

The analysis of protein glycosylation is critical in assessing various disease initiation and progression but complex due to the low abundance of glycopeptides. Zhang and co‐workers synthesized an amino‐functionalized MIL‐101(Cr)‐NH_2_ by using 2‐aminoterephthalic acid as a linker and reported a highly specific glycopeptide enrichment using this MOF. With a large surface area and strong hydrophilicity, the MOF could selectively enrich glycopeptides even under highly abundant protein suppression effect from a limited volume of a complex biological sample without any pretreatment. The study suggests the potential applications of MOFs in the field of glycoproteomics and glycomics.^[^ ^510^ ^]^ In a similar study, Ma et al. prepared a maltose‐functionalized MOF, MIL‐101(Cr)‐maltose, via two step post‐synthetic modification of MIL‐101(Cr)‐NH_2_. Using this MOF containing numerous hydrophilic maltose groups, highly efficient and sensitive glycopeptide enrichment was achieved from both standard glycopeptides and complex biological samples. This study introduces a generic functionalization route to design new materials from amino‐derived MOFs.^[^ ^518^ ^]^

**Figure 49:**
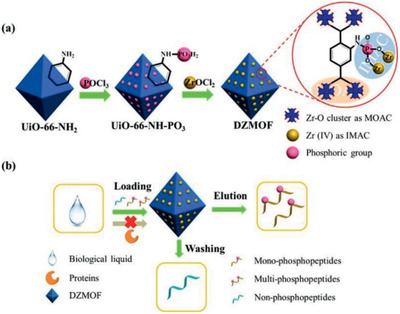
a) Synthesis of DZMOF. b) Enrichment of phosphopeptides from a biological sample. Reproduced with permission.^[^ ^526^ ^]^ Copyright 2016, American Chemical Society.

An interesting dual‐metal centered zirconium MOF (DZMOF) endowed with interactions suitable for both metal oxide affinity chromatography (MOAC) and immobilized metal ion affinity chromatography (IMAC) was produced by immobilizing zirconium(IV) on UiO‐66‐NH_2_. As shown in **Figure** **49**, amine functionalized UiO‐66 was first converted to phosphonate‐functionalized UiO‐66. The dual‐metal centers were caused by immobilized Zr(IV) along with the inherent Zr‐O cluster. DZMOF showed excellent performance as specific metal‐affinity probe towards the extraction of mono‐phosphorylated and multi‐phosphorylated peptides in MOAC and IMAC, respectively. Furthermore, unprecedented anti‐interference performance of DZMOF against the nonphosphorylated peptides was observed upon capturing of *β*‐casein from mixtures with a molar ratio up to 1:5000 for *β*‐casein versus bovine serum albumin. This work demonstrates the great potential of DZMOF in the extraction of low‐abundance phosphopeptides from complex biosamples.^[^ ^526^ ^]^

**Figure 50:**
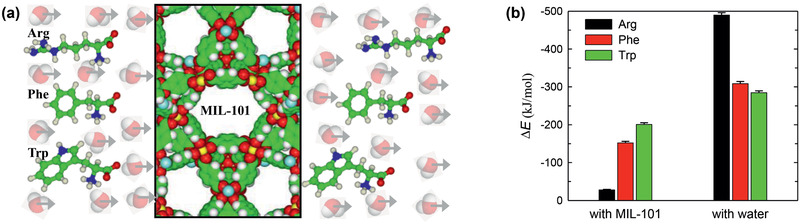
a) Separation of amino acids (Arg, Phe, and Trp) from aqueous solution by MIL‐101. b) Interaction energies of amino acids with MIL‐101 and water, respectively. Reproduced with permission.^[^ ^128^ ^]^ Copyright 2013, American Chemical Society.

There have been a handful of simulation studies reported to examine biomolecule encapsulation and separation by MOFs. Using non‐equilibrium MD simulation, Jiang and co‐workers investigated the capability of MIL‐101 for the separation of a mixture of amino acids from aqueous solution. As shown in **Figure** **50**, MIL‐101 was considered as a stationary phase; the mixture consisted of arginine (Arg), phenylalanine (Phe), and tryptophan (Trp), differing in molecular size, weight, and charge. The elution order was predicted to be Arg > Phe > Trp. Among the three amino acids, Arg exhibited the most hydrophilic and experienced the strongest interaction with water but the weakest interaction with MIL‐101, thus Arg transported the fastest. Furthermore, Arg was found to form the largest number of hydrogen‐bonds with water and the largest hydrophilic solvent‐accessible surface area. In contrast, Trp had the weakest interaction with water and the closest contact with MIL‐101. From detailed energetic and structural analysis, the underlying separation mechanism of the three amino acids was attributed to cooperative solute‐solvent and solute‐framework interactions. This simulation study suggests that MIL‐101 could be an interesting material for the separation of important biomolecules.^[^ ^128^ ^]^

**Figure 51:**
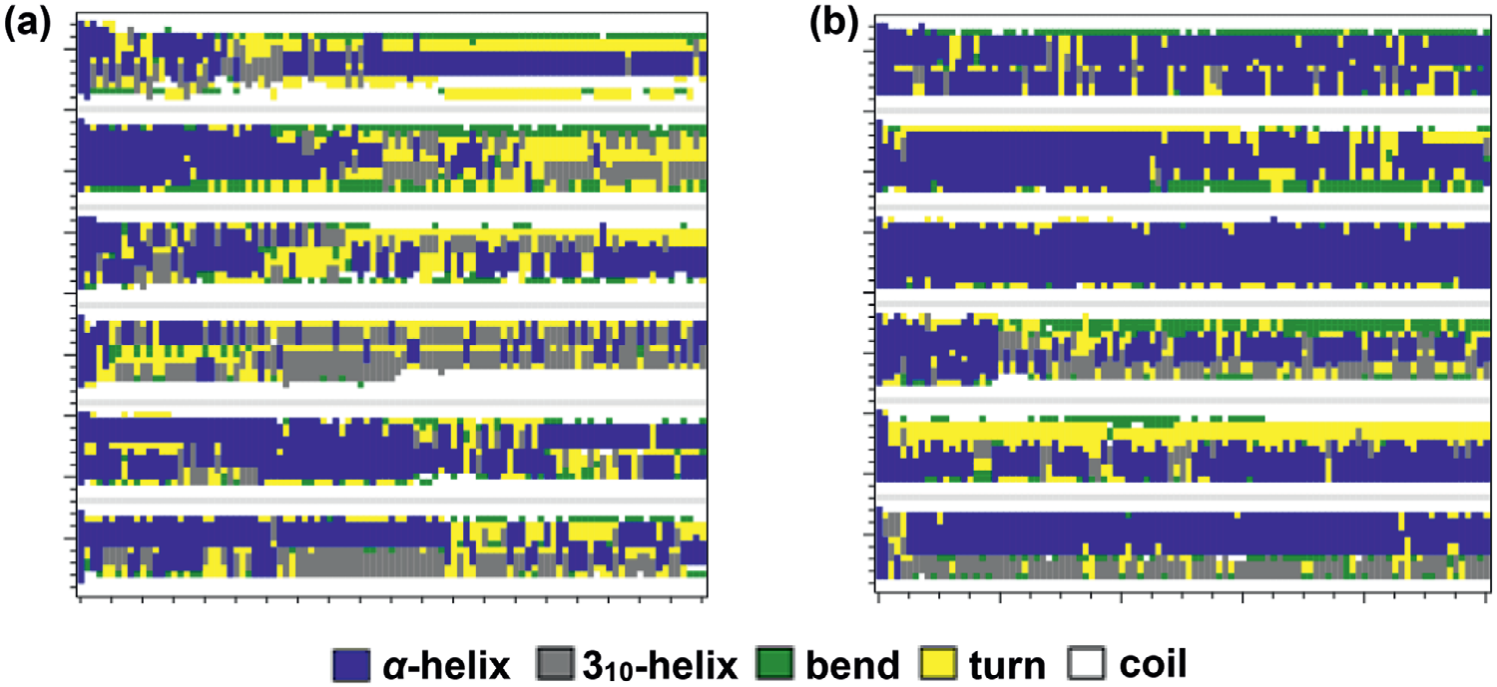
Structural evolution of six peptide chains of *β*‐adrenoceptor in a) IRMOF‐74‐II and b) IRMOF‐74‐III at 290 K. Reproduced with permission.^[^ ^531^ ^]^ Copyright 2016, Elsevier.

Jiang and co‐workers further reported a molecular simulation study to examine a helical peptide (*β*‐adrenoceptor) confined in two MOFs (IRMOF‐74‐II and ‐III). It was observed that the peptide in MOFs underwent reversible structural transformation between major (*α*‐helix and 3_10_‐helix) and minor structures (bend, turn, and coil). In IRMOF‐74‐II, the peptide was over‐confined and the helicity was lower compared with that in bulk water; however, the helicity was enhanced upon moderately confined in IRMOF‐74‐III (**Figure** **51**). The thermal fluctuations of the peptide in both MOFs were smaller than in bulk water due to the loss of conformational entropy. In contrast to the hydrophilic counterpart, the hydrophobic solvent‐accessible surface area of the peptide was largely reduced upon confined in both MOFs because of the favorable interactions between hydrophobic residues and MOFs. This study provides microscopic insight into peptide confined in MOFs and suggest a rational way of nanocarrier design for peptides and other biomolecules.^[^ ^531^ ^]^

**Figure 52:**
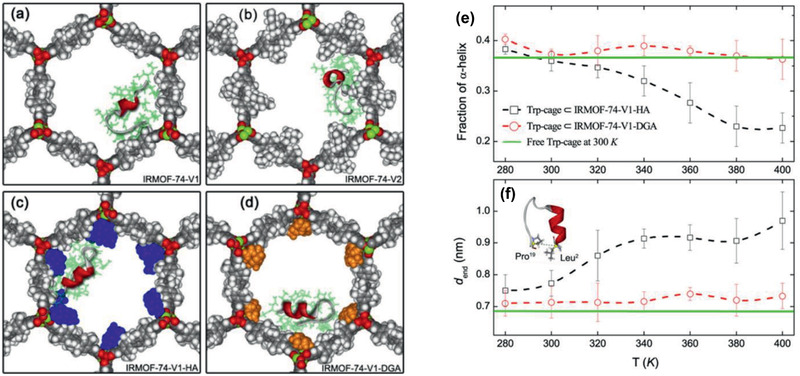
a–d) Equilibrated structures of Trp‐cage in IRMOF‐74‐Vs. e) Order parameters of *α*‐helix in Trp‐cage and f) distance between two ends of secondary structure in Trp‐cage versus temperature. Reproduced with permission.^[^ ^532^ ^]^ Copyright 2016, American Chemical Society.

Zhang et al. simulated the encapsulation of mini‐protein Trp‐cage in a series of IRMOF‐74s with different pore apertures and surface chemistries. To elucidate the effects of guest size, the monomers and tetramers of amino acid side chain analogues were employed for binding in IRMOF‐74‐I. Evaluation of binding energies revealed the van der Waals interaction as main driving force for binding and the guest size as a key factor for protein binding with MOFs. The equilibrated structures of Trp‐cage encapsulated in IRMOF‐74‐Vs were derived from simulations as shown in **Figure** **52**. Trp‐cage was observed to denature in IRMOF‐74‐V1 and IRMOF‐74‐V2 by the dissociation of *α*‐helix and/or detachment of *α*‐helix from loops. With increasing temperature, the fraction of *α*‐helix exhibited sharp decrease and the distance between two ends of secondary structure became longer for Trp‐cage⊂IRMOF‐74‐V1‐HA. However, Trp‐cage in IRMOF‐74‐V1‐DGA showed high stability and the fraction of *α*‐helix was well preserved. It was concluded that the encapsulation could be achieved by maintaining a polar/nonpolar balance on the MOF surface through tunable modification of organic linkers and Mg–O chelating moieties. This work presents guidelines for selective encapsulation of biomolecules within MOFs and would facilitate MOFs as a new class of host materials and molecular chaperones.^[^ ^532^ ^]^

Similar to drug delivery, MOFs for biomolecule encapsulation and bioseparation should be also biocompatible and non‐toxic. For encapsulation, guest–host interaction is crucial to long‐term usability. Other than enzymatic catalysis, guest molecules can impart new features to MOFs as molecular motors and sensors. For separation, MOFs are mostly investigated as stationary phase materials to enrich endogenous, glycol and phospopetides from complex biosamples. Being with the inherent features such as high surface area, tunable porosity and surface chemistry, MOFs provide an excellent platform for introducing amino acids/peptides into their frameworks to produce bioaffinity‐MOFs. These MOFs show enhanced properties in terms of high biocompatibility, low toxicity, multiple interaction sites, hydrophobic/hydrophilic affinities, and specific molecular recognition.

## 5 Summary and Outlook

**Figure 53:**
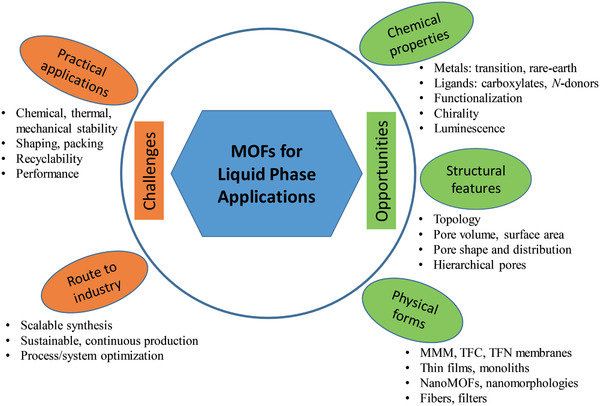
Challenges and opportunities of MOFs for liquid phase applications.

In the past several years, many interesting MOFs have been synthesized that are resistant to liquids and stable under harsh conditions. Remarkable progress and significant advance have been achieved to apply MOFs for liquid phase applications. We have summarized the recent experimental and computational studies on MOFs for liquid phase applications including liquid fuel purification and aromatics separation in energy sector, water treatment, solvent recovery and chemical sensing in environmental sector, as well as chiral separation, drug delivery, biomolecule encapsulation and separation in pharmaceutical sector. At present, these studies are largely at laboratory scale. Apparently, there is still a long way for MOFs to be practically utilized in industrial applications. A number of challenges and opportunities are schematically illustrated in **Figure** **53** for future research and development in this important field.

1) With de novo synthesis and postsynthetic modification, many chemically and thermally stable MOFs with different structures and functionalities have been synthesized. Nevertheless, this area is not completely mature and the continual quest for advanced methods is highly desired. Reduction of metal‐ligand lability, which is the main strategy to synthesize stable MOFs, brings several challenges such as slow crystallization and defect formation. It is thus important to investigate the mechanisms of MOF crystallization and growth.^[^ ^533^ ^]^ In addition to chemical and thermal stability, mechanical stability is equally important under practical application conditions. During the shaping and packing of MOFs, synthesized powders are densified typically at elevated pressures. Mechanical stability measures the capability of materials to withstand textural and functional properties under a mechanical load. A systematic approach combining sophisticated experimental and computational techniques is appealing to predict, characterize and design chemically, thermally and mechanically stable MOFs.^[^ ^534^, ^535^ ^]^ For long‐term recyclability, the structures and properties of MOF samples under practical cyclic operating conditions should be examined.
2) Crystalline nature is one of the key features of MOFs; consequently, it is easy to characterize structures, rationally design new functional MOFs, and develop structure‐property relationships. On the other hand, crystalline MOFs are most commonly available in a powder form, which is not favorable for processing and handling. For practical applications, MOF samples are required to be processed into different physical forms while preserving or even improving their properties. There have been ongoing efforts in this direction to fabricate MOFs into membranes, thin films, nanoMOFs and fibers. Exploration on macro MOF forms (aerogels, hydrogels and hierarchical structures) are currently limited but expected to rise,^[^ ^536^, ^537^, ^538^ ^]^ along with the continual research on low‐cost fabrication techniques for existing forms.
3) Liquids in practical applications are complex with compounds of different physical and chemical properties. Most MOFs examined have been focused on a specific liquid phase application. Multifunctional MOFs applicable for widespread applications in various liquids are very appealing. Particularly, MOFs containing hierarchical and/or different functional pores are highly desired.^[^ ^539^ ^]^ At present, postsynthetic modification is commonly used to impart multifunctionality into MOFs; however, rational evaluation of performance by this method is to a certain extent difficult. Thus, it is important to develop new de novo synthesis method to produce multifunctional all‐in‐one MOFs, and it will need judicious selection of building blocks with appropriate functionalities. Effective exploration of mixed‐metals, mixed‐linkers, and domain building blocks might provide a new avenue to produce multifunctional MOFs.^[^ ^540^, ^541^ ^]^
4) To realize the great potential of MOFs for practical liquid phase applications, it is of paramount importance to understand fundamental mechanisms and factors governing application performance. For the time being, experimental studies are predominant in this field by many advanced techniques such as X‐ray and electron diffractions, NMR, IR, and Raman spectroscopies. Nevertheless, interdisciplinary computational and experimental joint efforts are urgently needed.^[^ ^542^ ^]^ Computations can provide a wealth of atomic‐resolution and time‐resolved microscopic insights, which are instrumental to secure correct interpretation of experimental observations and establish quantitative structure‐property‐performance relationships.
5) There are primarily three types of computations for liquid phase applications. i) Grand‐canonical Monte Carlo method for liquid adsorption. Due to high density in a liquid phase, it is usually inefficient to sample molecular configurations and thus slow to achieve equilibration. This is particularly true for highly polar molecules like water and alcohols. ii) Molecular dynamics method for diffusion and permeation in a liquid phase. Taking ion diffusion in a dilute aqueous solution as an example, most of the simulation time is indeed used to sample the motion of water molecules rather than ions; thus, a long simulation duration is required to reliably capture ion diffusion. iii) First‐principles methods like density‐functional theory for binding energy of solute in a solvent. The common practice is to adopt polarizable continuum model with a certain dielectric constant to mimic the solvent. In such a treatment, the solvent molecules and their specific effects are not explicitly taken into account and thus may not be accurate. Nevertheless, with even‐increasing computational capability, resource and advanced algorithms, it is expected that these challenges will be progressively overcome.

We envision that MOFs are intriguing and versatile materials for a wide range of liquid phase applications. It is the prime time to move from laboratory to realistic tests under industrially relevant conditions. Towards high‐performance practical applications, more collective research endeavors are expected in developing new synthesis methods to produce stable and multifunctional MOFs, in preparing physical forms well‐suited for various applications, in quantitative and mechanistic understanding of key governing factors. As the demands for clean water, food, energy, high‐value chemicals, and high‐purity drugs continually rise, we foresee the rapid development of task‐specific MOFs for important and emerging liquid phase applications.

## Conflict of Interest

The authors declare no conflict of interest.

## References

[1] K. Ishizaki , S. Komarneni , M. Nanko , Porous Materials, Springer, Boston, MA 1998.

[2] C. Baerlocher , L. B. McCusker , *Database of Zeolite Structures* , http://www.iza-structure.org/databases/

[3] H. Furukawa , K. E. Cordova , M. O'Keeffe , O. M. Yaghi , Science 2013, 341, 1230444. 23990564 10.1126/science.1230444

[4] https://www.ccdc.cam.ac.uk/ (accessed: August 2020).

[5] G. Ferey , Chem. Soc. Rev. 2008, 37, 191. 18197340 10.1039/b618320b

[6] A. U. Czaja , N. Trukhan , U. Muller , Chem. Soc. Rev. 2009, 38, 1284. 19384438 10.1039/b804680h

[7] S. T. Meek , J. A. Greathouse , M. D. Allendorf , Adv. Mater. 2011, 23, 249. 20972981 10.1002/adma.201002854

[8] M. P. Suh , H. J. Park , T. K. Prasad , D. W. Lim , Chem. Rev. 2012, 112, 782. 22191516 10.1021/cr200274s

[9] R. B. Getman , Y. S. Bae , C. E. Wilmer , R. Q. Snurr , Chem. Rev. 2012, 112, 703. 22188435 10.1021/cr200217c

[10] K. Sumida , D. L. Rogow , J. A. Mason , T. M. McDonald , E. D. Block , Z. R. Herm , T. H. Bae , J. R. Long , Chem. Rev. 2012, 112, 724. 22204561 10.1021/cr2003272

[11] J. W. Jiang , Coordination Polymers and Metal–Organic Frameworks (Eds: O. L. Ortiz , L. D. Ramírez ), Nova Science Publishers, Hauppauge, NY 2012.

[12] J.‐R. Li , J. Sculley , H.‐C. Zhou , Chem. Rev. 2012, 112, 869. 21978134 10.1021/cr200190s

[13] G. Maurin , C. Serre , A. Cooper , G. Férey , Chem. Soc. Rev. 2017, 46, 3104. 28561090 10.1039/c7cs90049j

[14] O. M. Yaghi , G. M. Li , H. L. Li , Nature 1995, 378, 703.

[15] S. Mollick , S. Mukherjee , D. Kim , Z. Qiao , A. V. Desai , R. Saha , Y. D. More , J. W. Jiang , M. S. Lah , S. K. Ghosh , Angew. Chem., Int. Ed. 2019, 58, 1041. 10.1002/anie.201811037 30511777

[16] Y. Bai , Y. Dou , L. H. Xie , W. Rutledge , J. R. Li , H. C. Zhou , Chem. Soc. Rev. 2016, 45, 2327. 26886869 10.1039/c5cs00837a

[17] K. K. Tanabe , S. M. Cohen , Chem. Soc. Rev. 2011, 40, 498. 21103601 10.1039/c0cs00031k

[18] K. A. Cychosz , R. Ahmad , A. J. Matzger , Chem. Sci. 2010, 1, 293.

[19] B. Van de Voorde , B. Bueken , J. Denayer , D. De Vos , Chem. Soc. Rev. 2014, 43, 5766. 24647892 10.1039/c4cs00006d

[20] X. Li , Y. Liu , J. Wang , J. Gascon , J. Li , B. Van der Bruggen , Chem. Soc. Rev. 2017, 46, 7124. 29110013 10.1039/c7cs00575j

[21] C. Yin , G. Zhu , D. Xia , Fuel Process. Technol. 2002, 79, 135.

[22] C. Song , X. L. Ma , Appl. Catal., B 2003, 41, 207.

[23] K. A. Cychosz , A. G. Wong‐Foy , A. J. Matzger , J. Am. Chem. Soc. 2008, 130, 6938. 18465854 10.1021/ja802121u

[24] K. A. Cychosz , A. G. Wong‐Foy , A. J. Matzger , J. Am. Chem. Soc. 2009, 131, 14538. 19757809 10.1021/ja906034k

[25] J. K. Schnobrich , O. Lebel , K. A. Cychosz , A. Dailly , A. G. Wong‐Foy , A. J. Matzger , J. Am. Chem. Soc. 2010, 132, 13941. 20839886 10.1021/ja107423k

[26] T.‐H. Park , K. A. Cychosz , A. G. Wong‐Foy , A. Dailly , A. J. Matzger , Chem. Commun. 2011, 47, 1452. 10.1039/c0cc03482g 21132184

[27] S. Achmann , G. Hagen , M. Hammerle , I. Malkowsky , C. Kiener , R. Moos , Chem. Eng. Technol. 2010, 33, 275.

[28] N. A. Khan , J. W. Jun , J. H. Jeong , S. H. Jhung , Chem. Commun. 2011, 47, 1306. 10.1039/c0cc04759g 21152546

[29] N. A. Khan , S. H. Jhung , Angew. Chem., Int. Ed. 2012, 51, 1198. 10.1002/anie.201105113 22121078

[30] N. A. Khan , S. H. Jhung , Fuel Process. Technol. 2012, 100, 49.

[31] N. A. Khan , S. H. Jhung , J. Hazard. Mater. 2013, 260, 1050. 23892314 10.1016/j.jhazmat.2013.06.076

[32] N. A. Khan , Z. Hasan , S. H. Jhung , Chem. ‐ Eur. J. 2014, 20, 376. 24390909 10.1002/chem.201304291

[33] Z. Hasan , S. H. Jhung , ACS Appl. Mater. Interfaces 2015, 7, 10429. 25912936 10.1021/acsami.5b01642

[34] I. Ahmed , Z. Hasan , N. A. Khan , S. H. Jhung , Appl. Catal. B 2013, 129, 123.

[35] I. Ahmed , N. A. Khan , Z. Hasan , S. H. Jhung , J. Hazard. Mater. 2013, 250, 37. 23434477 10.1016/j.jhazmat.2013.01.024

[36] I. Ahmed , N. A. Khan , S. H. Jhung , Inorg. Chem. 2013, 52, 14155. 24299306 10.1021/ic402012d

[37] I. Ahmed , S. H. Jhung , Chem. Eng. J. 2014, 251, 35.

[38] I. Ahmed , J. W. Jun , B. K. Jung , S. H. Jhung , Chem. Eng. J. 2014, 255, 623.

[39] I. Ahmed , S. H. Jhung , J. Hazard. Mater. 2015, 283, 544. 25464294 10.1016/j.jhazmat.2014.10.002

[40] I. Ahmed , N. A. Khan , S. H. Jhung , Chem. Eng. J. 2017, 321, 40.

[41] N. A. Khan , D. K. Yoo , S. H. Jhung , ACS Appl. Mater. Interfaces 2018, 10, 35639. 30256614 10.1021/acsami.8b13256

[42] D. Peralta , G. Chaplais , A. Simon‐Masseron , K. Barthelet , G. D. Pirngruber , Energy Fuels 2012, 26, 4953. 10.1021/ja211864w 22397705

[43] S. L. Li , Y. Q. Lan , H. Sakurai , Q. Xu , Chem. ‐ Eur. J. 2012, 18, 16302. 23168579 10.1002/chem.201203093

[44] H. X. Zhang , H. L. Huang , C. X. Li , H. Meng , Y. Z. Lu , C. Zhong , D. Liu , Q. Yang , Ind. Eng. Chem. Res. 2012, 51, 12449.

[45] W.‐W. He , S.‐L. Li , W.‐L. Li , J.‐S. Li , G.‐S. Yang , S.‐R. Zhang , Y.‐Q. Lan , P. Shen , Z.‐M. Su , J. Mater. Chem. A 2013, 1, 11111.

[46] B. Van de Voorde , A. S. Munn , N. Guillou , F. Millange , D. E. De Vos , R. I. Walton , Phys. Chem. Chem. Phys. 2013, 15, 8606. 23439974 10.1039/c3cp44349c

[47] W. Dai , J. Hu , L. M. Zhou , S. Li , X. Hu , H. Huang , Energy Fuels 2013, 27, 816.

[48] S. Y. Jia , Y. F. Zhang , Y. Liu , F. X. Qin , H. T. Ren , S. H. Wu , J. Hazard. Mater. 2013, 262, 589. 24095999 10.1016/j.jhazmat.2013.08.056

[49] T. Jin , Q. Yang , C. Meng , J. Xu , H. L. Liu , J. Hu , H. Ling , RSC Adv. 2014, 4, 41902.

[50] A. L. Nuzhdin , K. A. Kovalenko , D. N. Dybtsev , G. A. Bukhtiyarova , Mendeleev Commun. 2010, 20, 57.

[51] M. Maes , L. Alaerts , F. Vermoortele , R. Ameloot , S. Couck , V. Finsy , J. F. M. Denayer , D. E. De Vos , J. Am. Chem. Soc. 2010, 132, 2284. 20121122 10.1021/ja9088378

[52] M. Maes , M. Trekels , M. Boulhout , S. Schouteden , F. Vermoortele , L. Alaerts , D. Heurtaux , Y.‐K. Seo , Y. K. Hwang , J.‐S. Chang , I. Beurroies , R. Denoyel , K. Temst , A. Vantomme , P. Horcajada , C. Serre , D. E. De Vos , Angew. Chem., Int. Ed. 2011, 50, 4210. 10.1002/anie.201100050 21472941

[53] B. Van de Voorde , M. Boulhout , F. Vermoortele , P. Horcajada , D. Cunha , J. S. Lee , J. S. Chang , E. Gibson , M. Daturi , J. C. Lavalley , A. Vimont , I. Beurroies , D. E. De Vos , J. Am. Chem. Soc. 2013, 135, 9849. 23646935 10.1021/ja403571z

[54] Z. Y. Wang , Z. G. Sun , L. H. Kong , G. Li , J. Energy Chem. 2013, 22, 869.

[55] L. Wu , J. Xiao , Y. Wu , S. Xian , G. Miao , H. Wang , Z. Li , Langmuir 2014, 30, 1080. 24432826 10.1021/la404540j

[56] S. N. Yu , Z. Y. Jiang , H. Ding , F. S. Pan , B. Y. Wang , J. Yang , X. Z. Cao , J. Membr. Sci. 2015, 481, 73.

[57] S. N. Yu , Z. Y. Jiang , W. D. Li , J. Q. Mayta , H. Ding , Y. M. Song , Z. Li , Z. W. Dong , F. S. Pan , B. Y. Wang , P. Zhang , X. Z. Cao , Chem. Eng. Process. 2018, 123, 12.

[58] S. N. Yu , F. S. Pan , S. Yang , H. Ding , Z. Y. Jiang , B. Y. Wang , Z. X. Li , X. Z. Cao , Chem. Eng. Sci. 2015, 135, 479.

[59] F. S. Pan , M. D. Wang , H. Ding , Y. M. Song , W. D. Li , H. Wu , Z. Y. Jiang , B. Y. Wang , X. Z. Cao , J. Membr. Sci. 2018, 552, 1.

[60] Y. Zhang , Z. Jiang , J. Song , J. Song , F. Pan , P. Zhang , X. Cao , Ind. Eng. Chem. Res. 2019, 58, 16911.

[61] X. Liang , Y. Zhang , Y. Qu , Y. Han , X. Wang , A. Cheng , A. Duan , L. Zhao , RSC Adv. 2018, 8, 13750. 10.1039/c8ra01490f PMC9079811 35539321

[62] B. L. Chen , Y. Y. Ji , M. Xue , F. R. Fronczek , E. J. Hurtado , J. U. Mondal , C. D. Liang , S. Dai , Inorg. Chem. 2008, 47, 5543. 18512902 10.1021/ic8004008

[63] J. Cousin Saint Remi , T. Remy , V. Van Hunskerken , S. van de Perre , T. Duerinck , M. Maes , D. De Vos , E. Gobechiya , C. E. A. Kirschhock , G. V. Baron , J. F. M. Denayer , ChemSusChem 2011, 4, 1074. 21744509 10.1002/cssc.201100261

[64] J. Cousin Saint Remi , G. Baron , J. Denayer , Adsorption 2012, 18, 367.

[65] Y. Chiang , W. Liang , S. Yang , C. R. Bond , W. You , R. P. Lively , S. Nair , ACS Sustainable Chem. Eng. 2019, 7, 16560.

[66] S. Takamizawa , C. Kachi‐Terajima , M. A. Kohbara , T. Akatsuka , T. Jin , Chem. ‐ Asian J. 2007, 2, 837. 17523183 10.1002/asia.200600404

[67] X. L. Liu , Y. S. Li , G. Q. Zhu , Y. J. Ban , L. Y. Xu , W. S. Yang , Angew. Chem., Int. Ed. 2011, 50, 10636. 10.1002/anie.201104383 21898739

[68] M. H. Huang , G. G. Chang , Y. Su , H. B. Xing , Z. G. Zhang , Y. W. Yang , Q. L. Ren , Z. B. Bao , B. L. Chen , Chem. Commun. 2015, 51, 12205. 10.1039/c5cc03648h 26136210

[69] N. A. Khan , Z. Hasan , S. H. Jhung , Chem. Commun. 2016, 52, 2561. 10.1039/c5cc08896h 26744746

[70] Z. P. Zhao , Z. Zuhra , L. B. Qin , Y. S. Zhou , L. J. Zhang , F. Tang , C. C. Mu , Fuel Process. Technol. 2018, 176, 276.

[71] Y. X. Hu , X. L. Dong , J. P. Nan , W. Q. Jin , X. M. Ren , N. P. Xu , Y. M. Lee , Chem. Commun. 2011, 47, 737. 10.1039/c0cc03927f 21060926

[72] X. L. Dong , Y. S. Lin , Chem. Commun. 2013, 49, 1196. 10.1039/c2cc38512k 23295282

[73] H. W. Fan , Q. Shi , H. Yan , S. L. Ji , J. X. Dong , G. J. Zhang , Angew. Chem., Int. Ed. 2014, 53, 5578. 10.1002/anie.201309534 24711246

[74] L. H. Wee , Y. Li , K. Zhang , P. Davit , S. Bordiga , J. W. Jiang , I. F. J. Vankelecom , J. A. Martens , Adv. Funct. Mater. 2015, 25, 516.

[75] L. Zhao , X. Yu , Y. Wang , J. Zhang , Y. Ying , Y. Cheng , S. B. Peh , G. Liu , X. Wang , Y. Cai , D. Zhao , J. Membr. Sci. 2020, 610, 118239.

[76] F. C. Wu , L. Lin , H. O. Liu , H. T. Wang , J. S. Qiu , X. F. Zhang , J. Membr. Sci. 2017, 544, 342.

[77] A. Nalaparaju , X. S. Zhao , J. W. Jiang , Energy Environ. Sci. 2011, 4, 2107.

[78] K. Zhang , A. Nalaparaju , Y. Chen , J. W. Jiang , Phys. Chem. Chem. Phys. 2014, 16, 9643. 24727907 10.1039/c4cp00739e

[79] K. Zhang , K. M. Gupta , Y. Chen , J. W. Jiang , AIChE J. 2015, 61, 2763.

[80] Y. Li , S. I. Sandler , D. G. Vlachos , C. J. Peng , H. L. Liu , Y. Hu , Ind. Eng. Chem. Res. 2011, 50, 14084.

[81] J. Fabri , U. Graeser , T. A. Simo , Xylenes: Ullmann's Encyclopedia of Industrial Chemistry, Wiley‐VCH Verlag GmbH & Co, New York 2000.

[82] W. J. Cannella , Xylenes and Ethylbenzene: Kirk‐Othmer Encyclopedia of Chemical Technology, John Wiley & Sons, New York 2000.

[83] L. Alaerts , C. E. A. Kirschhock , M. Maes , M. A. Van Der Veen , V. Finsy , A. Depla , J. A. Martens , G. V. Baron , P. A. Jacobs , J. F. M. Denayer , D. E. De Vos , Angew. Chem., Int. Ed. 2007, 46, 4293. 10.1002/anie.200700056 17477460

[84] L. Alaerts , M. Maes , L. Giebeler , P. A. Jacobs , J. A. Martens , J. F. M. Denayer , C. E. A. Kirschhock , D. E. De Vos , J. Am. Chem. Soc. 2008, 130, 14170. 18826226 10.1021/ja802761z

[85] L. Alaerts , M. Maes , P. A. Jacobs , J. F. M. Denayer , D. E. De Vos , Phys. Chem. Chem. Phys. 2008, 10, 2979. 18473046 10.1039/b719513c

[86] L. Alaerts , M. Maes , M. A. Van Der Veen , P. A. Jacobs , D. E. De Vos , Phys. Chem. Chem. Phys. 2009, 11, 2903. 19421505 10.1039/b823233d

[87] R. Ameloot , A. Liekens , L. Alaerts , M. Maes , A. Galarneau , B. Coq , G. Desmet , B. F. Sels , J. F. M. Denayer , D. E. De Vos , Eur. J. Inorg. Chem. 2010, 3735.

[88] M. Maes , F. Vermoortele , L. Alaerts , S. Couck , C. E. A. Kirschhock , J. F. M. Denayer , D. E. De Vos , J. Am. Chem. Soc. 2010, 132, 15277. 20942418 10.1021/ja106142x

[89] M. Maes , F. Vermoortele , L. Alaerts , J. F. M. Denayer , D. E. De Vos , J. Phys. Chem. C 2011, 115, 1051.

[90] M. Maes , S. Schouteden , K. Hirai , S. Furukawa , S. Kitagawa , D. E. De Vos , Langmuir 2011, 27, 9083. 21696201 10.1021/la2018049

[91] F. Vermoortele , M. Maes , P. Z. Moghadam , M. J. Lennox , F. Ragon , M. Boulhout , S. Biswas , K. G. M. Laurier , I. Beurroies , R. Denoyel , M. Roeffaers , N. Stock , T. Duren , C. Serre , D. E. De Vos , J. Am. Chem. Soc. 2011, 133, 18526. 22022950 10.1021/ja207287h

[92] M. Maes , F. Vermoortele , M. Boulhout , T. Boudewijns , C. Kirschhock , R. Ameloot , I. Beurroies , R. Denoyel , D. E. De Vos , Microporous Mesoporous Mater. 2012, 157, 82.

[93] R. E. Osta , A. Carlin‐Sinclair , N. Guillou , R. I. Walton , F. Vermoortele , M. Maes , D. E. De Vos , F. Millange , Chem. Mater. 2012, 24, 2781.

[94] W. De Malsche , S. Van der Perre , S. Silverans , M. Maes , D. E. De Vos , F. Lynen , J. F. M. Denayer , Microporous Mesoporous Mater. 2012, 162, 1.

[95] F. Niekiel , J. Lannoeye , H. Reinsch , A. S. Munn , A. Heerwig , I. Zizak , S. Kaskel , R. I. Walton , D. de Vos , P. Llewellyn , A. Lieb , G. Maurin , N. Stock , Inorg. Chem. 2014, 53, 4610. 24720876 10.1021/ic500288w

[96] J. Lannoeye , B. Van de Voorde , B. Bozbiyik , H. Reinsch , J. Denayer , D. De Vos , Microporous Mesoporous Mater. 2016, 226, 292.

[97] S. Van der Perre , A. Liekens , B. Bueken , D. E. De Vos , G. V. Baron , J. F. M. Denayer , J. Chromatogr. A 2016, 1469, 68. 27692645 10.1016/j.chroma.2016.09.057

[98] C. X. Yang , X. P. Yan , Anal. Chem. 2011, 83, 7144. 21809852 10.1021/ac201517c

[99] S. S. Liu , C. X. Yang , S. W. Wang , X. P. Yan , Analyst 2012, 137, 816. 22159194 10.1039/c2an15925b

[100] C. X. Yang , S. S. Liu , H. F. Wang , S. W. Wang , X. P. Yan , Analyst 2012, 137, 133. 22034617 10.1039/c1an15600d

[101] Y. Y. Fu , C. X. Yang , X. P. Yan , Langmuir 2012, 28, 6794. 22480159 10.1021/la300298e

[102] Y. Y. Fu , C. X. Yang , X. P. Yan , J. Chromatogr. A 2013, 1274, 137. 23290359 10.1016/j.chroma.2012.12.015

[103] Y.‐Y. Fu , C.‐X. Yang , X.‐P. Yan , Chem. Commun. 2013, 49, 7162. 10.1039/c3cc43017k 23835621

[104] L.‐H. Liu , C.‐X. Yang , X.‐P. Yan , J. Chromatogr. A 2017, 1479, 137. 27939601 10.1016/j.chroma.2016.12.004

[105] H.‐Y. Huang , C.‐L. Lin , C.‐Y. Wu , Y.‐J. Cheng , C.‐H. Lin , Anal. Chim. Acta 2013, 779, 96. 23663677 10.1016/j.aca.2013.03.071

[106] R. Ahmad , A. G. Wong‐Foy , A. J. Matzger , Langmuir 2009, 25, 11977. 19754060 10.1021/la902276a

[107] A. Henschel , I. Senkovska , S. Kaskel , Adsorption 2011, 17, 219.

[108] M. A. Moreira , J. C. Santos , A. F. P. Ferrreira , J. M. Loureiro , F. Ragon , P. Horcajada , K. E. Shim , Y. K. Hwang , U. H. Lee , J. S. Chang , C. Serre , A. E. Rodrigues , Langmuir 2011, 28, 5715. 10.1021/la3004118 22404208

[109] D. Peralta , G. Chaplais , J. L. Paillaud , A. Simon‐Masseron , K. Barthelet , G. D. Pirngruber , Microporous Mesoporous Mater. 2013, 173, 1.

[110] A. Centrone , E. E. Santiso , T. A. Hatton , Small 2011, 7, 2356. 21626684 10.1002/smll.201100098

[111] A. Ahmed , N. Hodgson , M. Barrow , R. Clowes , C. M. Robertson , A. Steiner , P. McKeown , D. Bradshaw , P. Myers , H. F. Zhang , J. Mater. Chem. A 2014, 2, 9085.

[112] W. W. Zhao , C. Y. Zhang , Z. G. Yan , L. P. Bai , X. Y. Wang , H. L. Huang , Y. Y. Zhou , Y. B. Xie , F. S. Li , J. R. Li , J. Chromatogr. A 2014, 1370, 121. 25454136 10.1016/j.chroma.2014.10.036

[113] Z. M. Yan , W. M. Zhang , J. Gao , Y. F. Lin , J. R. Li , Z. Lin , L. Zhang , RSC Adv. 2015, 5, 40094.

[114] L. Diestel , H. Bux , D. Wachsmuth , J. Caro , Microporous Mesoporous Mater. 2012, 164, 288.

[115] Z. Kang , J. Ding , L. Fan , M. Xue , D. Zhang , L. Gao , S. Qiu , Inorg. Chem. Commun. 2013, 30, 74.

[116] A. Kasik , Y. S. Lin , Sep. Purif. Technol. 2014, 121, 38.

[117] A. Ibrahim , Y. S. Lin , Ind. Eng. Chem. Res. 2016, 55, 8652.

[118] C. Zhao , N. X. Wang , L. Wang , H. L. Huang , R. Zhang , F. Yang , Y. B. Xie , S. L. Ji , J. R. Li , Chem. Commun. 2014, 50, 13921. 10.1039/c4cc05279j 25260031

[119] Y. Zhang , N. X. Wang , S. L. Ji , R. Zhang , C. Zhao , J. R. Li , J. Membr. Sci. 2015, 489, 144.

[120] C. Zhao , N. X. Wang , L. Wang , S. N. Sheng , H. W. Fan , F. Yang , S. L. Ji , J. R. Li , J. M. Yu , AIChE J. 2016, 62, 3706.

[121] Y. Zhang , N. X. Wang , C. Zhao , L. Wang , S. L. Ji , J. R. Li , J. Membr. Sci. 2016, 520, 646.

[122] W. W. Zhao , C. Y. Zhang , Z. G. Yan , Y. Y. Zhou , J. R. Li , Y. B. Xie , L. P. Bai , L. Jiang , F. S. Li , PLoS One 2017, 12, 0178513.

[123] S. Chen , X. X. Li , L. Shu , P. Somsundaran , J. R. Li , J. Chromatogr. A 2017, 1510, 25. 28662853 10.1016/j.chroma.2017.06.033

[124] L. D. Tran , J. L. Ma , A. G. Wong‐Foy , A. J. Matzger , Chem. ‐ Eur. J. 2016, 22, 5509. 26868729 10.1002/chem.201600526

[125] A. L. Nuzhdin , A. S. Shalygin , E. A. Artiukha , A. M. Chibiryaev , G. A. Bukhtiyarova , O. N. Martyanov , RSC Adv. 2016, 6, 62501.

[126] S. Ehrling , C. Kutzscher , P. Freund , P. Muller , I. Senkovska , S. Kaskel , Microporous Mesoporous Mater. 2018, 263, 268.

[127] M. I. Gonzalez , M. T. Kapelewski , E. D. Bloch , P. J. Milner , D. A. Reed , M. R. Hudson , J. A. Mason , G. Barin , C. M. Brown , J. R. Long , J. Am. Chem. Soc. 2018, 140, 3412. 29446932 10.1021/jacs.7b13825 PMC8224533

[128] Z. Q. Hu , Y. F. Chen , J. W. Jiang , Langmuir 2013, 29, 1650. 23297767 10.1021/la3045972

[129] A. Torres‐Knoop , R. Krishna , D. Dubbeldam , Angew. Chem., Int. Ed. 2014, 53, 7774. 10.1002/anie.201402894 24916723

[130] M. Agrawal , S. Bhattacharyya , Y. Huang , K. C. Jayachandrababu , C. R. Murdock , J. A. Bentley , A. Rivas‐Cardona , M. M. Mertens , K. S. Walton , D. S. Sholl , S. Nair , J. Phys. Chem. C 2018, 122, 386.

[131] J. A. Gee , K. Zhang , S. Bhattacharyya , J. Bentley , M. Rungta , J. S. Abichandani , D. S. Sholl , S. Nair , J. Phys. Chem. C 2016, 120, 12075.

[132] X. Wu , W. Wei , J. W. Jiang , J. Caro , A. Huang , Angew. Chem., Int. Ed. 2018, 57, 15354. 10.1002/anie.201807935 30248220

[133] F. L. Fu , Q. Wang , J. Environ. Manage. 2011, 92, 407. 21138785 10.1016/j.jenvman.2010.11.011

[134] L. W. Mi , H. W. Hou , Z. Y. Song , H. Y. Han , H. Xu , Y. T. Fan , S. W. Ng , Cryst. Growth Des. 2007, 7, 2553.

[135] L. W. Mi , H. W. Hou , Z. Y. Song , H. Y. Han , Y. T. Fan , Chem. ‐ Eur. J. 2008, 14, 1814. 18092315 10.1002/chem.200700782

[136] M. Plabst , L. B. McCusker , T. Bein , J. Am. Chem. Soc. 2009, 131, 18112. 19921820 10.1021/ja904636y

[137] Q.‐R. Fang , D.‐Q. Yuan , J. Sculley , J.‐R. Li , Z.‐B. Han , H.‐C. Zhou , Inorg. Chem. 2010, 49, 11637. 21082837 10.1021/ic101935f

[138] Y. Wang , G. Ye , H. Chen , X. Hu , Z. Niu , S. Ma , J. Mater. Chem. A 2015, 3, 15292.

[139] M. Mon , F. Lloret , J. Ferrando‐Soria , C. Martí‐Gastaldo , D. Armentano , E. Pardo , Angew. Chem., Int. Ed. 2016, 55, 11167. 10.1002/anie.201606015 27529544

[140] F. Ke , L. G. Qiu , Y. P. Yuan , F. M. Peng , X. Jiang , A. J. Xie , Y. H. Shen , J. F. Zhu , J. Hazard. Mater. 2011, 196, 36. 21924826 10.1016/j.jhazmat.2011.08.069

[141] B. J. Zhu , X. Y. Yu , Y. Jia , F. M. Peng , B. Sun , M. Y. Zhang , T. Luo , J. H. Liu , X. J. Huang , J. Phys. Chem. C 2012, 116, 8601.

[142] A. Shahat , H. M. A. Hassan , H. M. E. Azzazy , Anal. Chim. Acta 2013, 793, 90. 23953211 10.1016/j.aca.2013.07.012

[143] K.‐K. Yee , N. Reimer , J. Liu , S.‐Y. Cheng , S.‐M. Yiu , J. Weber , N. Stock , Z. Xu , J. Am. Chem. Soc. 2013, 135, 7795. 23646999 10.1021/ja400212k

[144] S. D. T. , L. Peng , W. S. Reeder , S. M. Moosavi , D. Tiana , D. K. Britt , E. Oveisi , W. L. Queen , ACS Cent. Sci. 2018, 4, 349. 29632880 10.1021/acscentsci.7b00605 PMC5879484

[145] Y. Zhang , X. Zhao , H. Huang , Z. Li , D. Liu , C. Zhong , RSC Adv. 2015, 5, 72107.

[146] X. Meng , R.‐L. Zhong , X.‐Z. Song , S.‐Y. Song , Z.‐M. Hao , M. Zhu , S.‐N. Zhao , H.‐J. Zhang , Chem. Commun. 2014, 50, 6406. 10.1039/c4cc00553h 24811275

[147] L. Wang , X. Zhao , J. Zhang , Z. Xiong , Environ. Sci. Pollut. Res. 2017, 24, 14198. 10.1007/s11356-017-9002-9 28421521

[148] A. R. Geisse , C. M. Ngule , D. T. Genna , Chem. Commun. 2020, 56, 237. 10.1039/c9cc09022c 31803866

[149] B. Aguila , D. Banerjee , Z. Nie , Y. Shin , S. Ma , P. K. Thallapally , Chem. Commun. 2016, 52, 5940. 10.1039/c6cc00843g 27055254

[150] Y. Peng , H. Huang , D. Liu , C. Zhong , ACS Appl. Mater. Interfaces 2016, 8, 8527. 26999358 10.1021/acsami.6b00900

[151] C. Yu , Z. Shao , L. Liu , H. Hou , Cryst. Growth Des. 2018, 18, 3082.

[152] Y. Feng , H. Jiang , S. Li , J. Wang , X. Jing , Y. Wang , M. Chen , Colloids Surf. A 2013, 431, 87.

[153] M. Carboni , C. W. Abney , S. B. Liu , W. B. Lin , Chem. Sci. 2013, 4, 2396.

[154] W. Yang , Z.‐Q. Bai , W.‐Q. Shi , L.‐Y. Yuan , T. Tian , Z.‐F. Chai , H. Wang , Z.‐M. Sun , Chem. Commun. 2013, 49, 10415. 10.1039/c3cc44983a 24079004

[155] B.‐C. Luo , L.‐Y. Yuan , Z.‐F. Chai , W.‐Q. Shi , Q. Tang , J. Radioanal. Nucl. Chem. 2016, 307, 269.

[156] S. Liu , M. Luo , J. Li , F. Luo , L. Ke , J. Ma , J. Radioanal. Nucl. Chem. 2016, 310, 353.

[157] Z. Wang , Q. Meng , R. Ma , Z. Wang , Y. Yang , H. Sha , X. Ma , X. Ruan , X. Zou , Y. Yuan , G. Zhu , Chem 2020, 6, 1683.

[158] J. Ai , F.‐Y. Chen , C.‐Y. Gao , H.‐R. Tian , Q.‐J. Pan , Z.‐M. Sun , Inorg. Chem. 2018, 57, 4419. 29570281 10.1021/acs.inorgchem.8b00099

[159] L. Huang , J. Cai , M. He , B. Chen , B. Hu , Ind. Eng. Chem. Res. 2018, 57, 6201.

[160] Z. A. Ghazi , A. M. Khattak , R. Iqbal , R. Ahmad , A. A. Khan , M. Usman , F. Nawaz , W. Ali , Z. Felegari , S. U. Jan , A. Iqbal , A. Ahmad , New J. Chem. 2018, 42, 10234.

[161] Y. Georgiou , J. A. Perman , A. B. Bourlinos , Y. Deligiannakis , J. Phys. Chem. C 2018, 122, 4859.

[162] A. Mehdinia , D. Jahedi Vaighan , A. Jabbari , ACS Sustainable Chem. Eng. 2018, 6, 3176.

[163] L. Esrafili , V. Safarifard , E. Tahmasebi , M. D. Esrafili , A. Morsali , New J. Chem. 2018, 42, 8864.

[164] R. Custelcean , V. Sellin , B. A. Moyer , Chem. Commun. 2007, 43, 1541. 10.1039/b616761f 17406701

[165] X. Zhao , D. Liu , H. Huang , W. Zhang , Q. Yang , C. Zhong , Microporous Mesoporous Mater. 2014, 185, 72.

[166] N. Zhang , X. Yang , X. Yu , Y. Jia , J. Wang , L. Kong , Z. Jin , B. Sun , T. Luo , J. Liu , Chem. Eng. J. 2014, 252, 220.

[167] Y. Wu , Y. Xie , F. Zhong , J. Gao , J. Yao , Microporous Mesoporous Mater. 2020, 306, 110386.

[168] H.‐R. Fu , Z.‐X. Xu , J. Zhang , Chem. Mater. 2015, 27, 205.

[169] Q. Zhang , J. Yu , J. Cai , L. Zhang , Y. Cui , Y. Yang , B. Chen , G. Qian , Chem. Commun. 2015, 51, 14732. 10.1039/c5cc05927e 26291500

[170] A. V. Desai , B. Manna , A. Karmakar , A. Sahu , S. K. Ghosh , Angew. Chem., Int. Ed. 2016, 55, 7811. 10.1002/anie.201600185 26855323

[171] L. Aboutorabi , A. Morsali , E. Tahmasebi , O. Büyükgüngor , Inorg. Chem. 2016, 55, 5507. 27195982 10.1021/acs.inorgchem.6b00522

[172] H. H. Fei , D. L. Rogow , S. R. J. Oliver , J. Am. Chem. Soc. 2010, 132, 7202. 20426466 10.1021/ja102134c

[173] C. Wang , X. Liu , J. P. Chen , K. Li , Sci. Rep. 2015, 5, 16613. 26559001 10.1038/srep16613 PMC4642326

[174] C. O. Audu , H. G. T. Nguyen , C.‐Y. Chang , M. J. Katz , L. Mao , O. K. Farha , J. T. Hupp , S. T. Nguyen , Chem. Sci. 2016, 7, 6492. 28451107 10.1039/c6sc00490c PMC5355942

[175] D. Xie , Y. Ma , Y. Gu , H. Zhou , H. Zhang , G. Wang , Y. Zhang , H. Zhao , J. Mater. Chem. A 2017, 5, 23794.

[176] Y. Chen , F. Chen , S. Zhang , Y. Cai , S. Cao , S. Li , W. Zhao , S. Yuan , X. Feng , A. Cao , X. Ma , B. Wang , J. Am. Chem. Soc. 2017, 139, 16482. 29083177 10.1021/jacs.7b10265

[177] A. J. Howarth , M. J. Katz , T. C. Wang , A. E. Platero‐Prats , K. W. Chapman , J. T. Hupp , O. K. Farha , J. Am. Chem. Soc. 2015, 137, 7488. 26000611 10.1021/jacs.5b03904

[178] M. B. Luo , Y. Y. Xiong , H. Q. Wu , X. F. Feng , J. Q. Li , F. Luo , Angew. Chem., Int. Ed. 2017, 56, 16376. 10.1002/anie.201709197 29094516

[179] D. Ma , G. Han , S. B. Pen , S. B. Chen , Ind. Eng. Chem. Res. 2017, 56, 12773.

[180] W. Liang , L. Li , J. Hou , N. D. Shepherd , T. D. Bennett , D. M. D'Alessandro , V. Chen , Chem. Sci. 2018, 9, 3508. 29780481 10.1039/c7sc05175a PMC5934739

[181] S. H. Jhung , J. H. Lee , J. W. Yoon , C. Serre , G. Ferey , J. S. Chang , Adv. Mater. 2007, 19, 121.

[182] E. Haque , J. E. Lee , I. T. Jang , Y. K. Hwang , J. S. Chang , J. Jegal , S. H. Jhung , J. Hazard. Mater. 2010, 181, 535. 10.1016/j.jhazmat.2010.05.047 20627406

[183] E. Haque , J. W. Jun , S. H. Jhung , J. Hazard. Mater. 2011, 185, 507. 20933323 10.1016/j.jhazmat.2010.09.035

[184] J. Cai , X. Mao , W.‐G. Song , Mater. Chem. Front. 2018, 2, 1389.

[185] J. Z. Gu , W. G. Lu , L. Jiang , H. C. Zhou , T. B. Lu , Inorg. Chem. 2007, 46, 5835. 17595076 10.1021/ic7004908

[186] T. R. C. Van Assche , T. Remy , G. Desmet , G. V. Baron , J. F. M. Denayer , Sep. Purif. Technol. 2011, 82, 76.

[187] C. Chen , M. Zhang , Q. X. Guan , W. Li , Chem. Eng. J. 2012, 183, 60.

[188] M. Maes , S. Schouteden , L. Alaerts , D. Depla , D. E. De Vos , Phys. Chem. Chem. Phys. 2011, 13, 5587. 21359287 10.1039/c0cp01703e

[189] D. V. Patil , P. B. S. Rallapalli , G. P. Dangi , R. J. Tayade , R. S. Somani , H. C. Bajaj , Ind. Eng. Chem. Res. 2011, 50, 10516.

[190] S. H. Huo , X. P. Yan , J. Mater. Chem. 2012, 22, 7449.

[191] F. Pu , X. Liu , B. Xu , J. Ren , X. Qu , Chem. ‐ Eur. J. 2012, 18, 4322. 22362560 10.1002/chem.201103524

[192] Y. Hu , Y. Fang , Z. Huang , C. Song , G. Li , Chem. Commun. 2012, 48, 3966. 10.1039/c2cc17048e 22419993

[193] X. S. Wang , J. Liu , J. M. Bonefont , D. Q. Yuan , P. K. Thallapally , S. Q. Ma , Chem. Commun. 2013, 49, 1533. 10.1039/c2cc38067f 23321927

[194] Z. L. Li , H. Li , X. Y. Guan , J. J. Tang , Y. R. Yusran , Z. Li , M. Xue , Q. R. Fang , Y. S. Yan , V. Valtchev , S. L. Qiu , J. Am. Chem. Soc. 2017, 139, 17771. 29179538 10.1021/jacs.7b11283

[195] K. A. Cychosz , A. J. Matzger , Langmuir 2011, 26, 17198. 10.1021/la103234u 20923216

[196] Z. Hasan , J. Jeon , S. H. Jhung , J. Hazard. Mater. 2012, 209, 151. 22277335 10.1016/j.jhazmat.2012.01.005

[197] J. Y. Song , B. N. Bhadra , N. A. Khan , S. H. Jhung , Microporous Mesoporous Mater. 2018, 260, 1.

[198] M. Barylak , K. Cendrowski , E. Mijowska , Ind. Eng. Chem. Res. 2018, 57, 4867.

[199] I. Akpinar , A. O. Yazaydin , J. Chem. Eng. Data 2018, 63, 2368.

[200] J. Dong , F. Xu , Z. Dong , Y. Zhao , Y. Yan , H. Jin , Y. Li , RSC Adv. 2018, 8, 19075. 10.1039/c8ra01968a PMC9080657 35539668

[201] M. Samal , J. Panda , B. P. Biswal , R. Sahu , CrystEngComm 2018, 20, 2486.

[202] Y. Yang , Z. Niu , H. Li , Y. Ma , Y. Zhang , H. Wang , Dalton Trans. 2018, 47, 6538. 29696290 10.1039/c8dt00184g

[203] C. Liu , A. Huang , New J. Chem. 2018, 42, 2372.

[204] W. Wu , M. Jia , J. Su , Z. Li , W. Li , AIChE J. 2020, 66, 16238.

[205] Z. Q. Hu , Y. F. Chen , J. W. Jiang , J. Chem. Phys. 2011, 134, 134705. 21476767 10.1063/1.3573902

[206] K. M. Gupta , K. Zhang , J. W. Jiang , Langmuir 2015, 31, 13230. 26588699 10.1021/acs.langmuir.5b03593

[207] K. M. Gupta , Z. Qiao , K. Zhang , J. W. Jiang , ACS Appl. Mater. Interfaces 2016, 8, 13392. 27195441 10.1021/acsami.6b01626

[208] K. M. Gupta , J. W. Jiang , ChemistrySelect 2017, 2, 3981.

[209] K. M. Gupta , J. Liu , J. W. Jiang , J. Membr. Sci. 2019, 572, 676.

[210] A. Nalaparaju , J. W. Jiang , J. Phys. Chem. C 2012, 116, 6925.

[211] A. Nalaparaju , J. W. Jiang , Langmuir 2012, 28, 15305. 23078288 10.1021/la3034116

[212] W. Wei , K. Zhang , Z. Qiao , J. W. Jiang , Chem. Eng. J. 2019, 356, 737.

[213] Y. Xiao , T. Han , G. Xiao , Y. Ying , H. Huang , Q. Yang , D. Liu , C. Zhong , Langmuir 2014, 30, 12229. 25251810 10.1021/la5030795

[214] S. Lin , D. H. Kumar Reddy , J. K. Bediako , M.‐H. Song , W. Wei , J.‐A. Kim , Y.‐S. Yun , J. Mater. Chem. A 2017, 5, 13557.

[215] Y. Zhu , K. M. Gupta , Q. Liu , J. W. Jiang , J. Caro , A. Huang , Desalination 2016, 385, 75.

[216] C. Liu , Y. Jiang , A. Nalaparaju , J. W. Jiang , A. Huang , J. Mater. Chem. A 2019, 7, 24205.

[217] S. Basu , M. Maes , A. Cano‐Odena , L. Alaerts , D. E. De Vos , I. F. J. Vankelecom , J. Membr. Sci. 2009, 344, 190.

[218] J. Campbell , G. Székely , R. P. Davies , D. C. Braddock , A. G. Livingston , J. Mater. Chem. A 2014, 2, 9260.

[219] J. Campbell , J. D. S. Burgal , G. Szekely , R. P. Davies , D. C. Braddock , A. Livingston , J. Membr. Sci. 2016, 503, 166.

[220] L. Zhu , H. Yu , H. Zhang , J. Shen , L. Xue , C. Gao , B. Van Der Bruggen , RSC Adv. 2015, 5, 73068.

[221] N. A. A. Sani , W. J. Lau , A. F. Ismail , RSC Adv. 2015, 5, 13000.

[222] N. A. A. Sani , W. J. Lau , N. A. H. M. Nordin , A. F. Ismail , Chem. Eng. Res. Des. 2016, 115, 66.

[223] J. Dai , S. Li , J. Liu , J. He , J. Li , L. Wang , J. Lei , J. Membr. Sci. 2019, 589, 117261.

[224] Z. Wang , Z. Si , D. Cai , G. Li , S. Li , P. Qin , T. Tan , Sep. Purif. Technol. 2019, 227, 115687.

[225] S. Sorribas , P. Gorgojo , C. Téllez , J. Coronas , A. G. Livingston , J. Am. Chem. Soc. 2013, 135, 15201. 24044635 10.1021/ja407665w

[226] C. Echaide‐Górriz , S. Sorribas , C. Téllez , J. Coronas , RSC Adv. 2016, 6, 90417.

[227] C. Echaide‐Górriz , M. Navarro , C. Téllez , J. Coronas , Dalton Trans. 2017, 46, 6244. 28443865 10.1039/c7dt00197e

[228] L. Sarango , L. Paseta , M. Navarro , B. Zornoza , J. Coronas , J. Ind. Eng. Chem. 2018, 59, 8.

[229] H. Yang , N. Wang , L. Wang , H. X. Liu , Q. F. An , S. Ji , J. Membr. Sci. 2018, 545, 158.

[230] X. Cheng , X. Jiang , Y. Zhang , C. H. Lau , Z. Xie , D. Ng , S. J. D. Smith , M. R. Hill , L. Shao , ACS Appl. Mater. Interfaces 2017, 9, 38877. 29022696 10.1021/acsami.7b07373

[231] X. Guo , D. Liu , T. Han , H. Huang , Q. Yang , C. Zhong , AIChE J. 2017, 63, 1303.

[232] X. Q. Cheng , K. Konstas , C. M. Doherty , C. D. Wood , X. Mulet , Z. Xie , D. Ng , M. R. Hill , L. Shao , C. H. Lau , ACS Appl. Mater. Interfaces 2017, 9, 14401. 28375614 10.1021/acsami.7b02295

[233] S.‐J. Xu , Q. Shen , G.‐E. Chen , Z.‐L. Xu , ACS Omega 2018, 3, 11770. 30320272 10.1021/acsomega.8b01808 PMC6173514

[234] L. Paseta , M. Navarro , J. Coronas , C. Téllez , J. Ind. Eng. Chem. 2019, 77, 344.

[235] M. Navarro , J. Benito , L. Paseta , I. Gascón , J. Coronas , C. Téllez , ACS Appl. Mater. Interfaces 2018, 10, 1278. 29243908 10.1021/acsami.7b17477

[236] C. Li , S. Li , L. Tian , J. Zhang , B. Su , M. Z. Hu , J. Membr. Sci. 2019, 572, 520.

[237] Y. Li , L. H. Wee , A. Volodin , J. A. Martens , I. F. J. Vankelecom , Chem. Commun. 2015, 51, 918. 10.1039/c4cc06699e 25435391

[238] J. Campbell , R. P. Davies , D. C. Braddock , A. G. Livingston , J. Mater. Chem. A 2015, 3, 9668.

[239] H. M. Tham , S. Japip , D. Hua , T.‐S. Chung , ChemSusChem 2018, 11, 2612. 29905033 10.1002/cssc.201800740

[240] M. Amirilargani , R. B. Merlet , P. Hedayati , A. Nijmeijer , L. Winnubst , L. C. P. M. de Smet , E. J. R. Sudhölter , Chem. Commun. 2019, 55, 4119. 10.1039/c9cc01624d 30889233

[241] J. Liu , S. Wang , T. Huang , P. Manchanda , E. Abou‐Hamad , S. P. Nunes , Sci. Adv. 2020, 6, eabb3188. 32875111 10.1126/sciadv.abb3188 PMC7438094

[242] W. Wei , K. M. Gupta , J. Liu , J. W. Jiang , ACS Appl. Mater. Interfaces 2018, 10, 33135. 30203646 10.1021/acsami.8b08364

[243] W. Wei , J. Liu , J. W. Jiang , ACS Sustainable Chem. Eng. 2019, 7, 1734.

[244] T. M. Reineke , M. Eddaoudi , M. Fehr , D. Kelley , O. M. Yaghi , J. Am. Chem. Soc. 1999, 121, 1651.

[245] B. Zhao , X.‐Y. Chen , P. Cheng , D.‐Z. Liao , S.‐P. Yan , Z.‐H. Jiang , J. Am. Chem. Soc. 2004, 126, 15394. 15563162 10.1021/ja047141b

[246] B. Zhao , H.‐L. Gao , X.‐Y. Chen , P. Cheng , W. Shi , D.‐Z. Liao , S.‐P. Yan , Z.‐H. Jiang , Chem. ‐ Eur. J. 2006, 12, 149.

[247] M. D. Allendorf , C. A. Bauer , R. K. Bhakta , R. J. T. Houk , Chem. Soc. Rev. 2009, 38, 1330. 19384441 10.1039/b802352m

[248] J. Tao , J.‐X. Shi , M.‐L. Tong , X.‐X. Zhang , X.‐M. Chen , Inorg. Chem. 2001, 40, 6328. 11703139 10.1021/ic010472u

[249] J. Lefebvre , R. J. Batchelor , D. B. Leznoff , J. Am. Chem. Soc. 2004, 126, 16117. 15584747 10.1021/ja049069n

[250] W. Liu , T. Jiao , Y. Li , Q. Liu , M. Tan , H. Wang , L. Wang , J. Am. Chem. Soc. 2004, 126, 2280. 14982412 10.1021/ja036635q

[251] E. Y. Lee , S. Y. Jang , M. P. Suh , J. Am. Chem. Soc. 2005, 127, 6374. 15853345 10.1021/ja043756x

[252] A. Douvali , A. C. Tsipis , S. V. Eliseeva , S. Petoud , G. S. Papaefstathiou , C. D. Malliakas , I. Papadas , G. S. Armatas , I. Margiolaki , M. G. Kanatzidis , T. Lazarides , M. J. Manos , Angew. Chem., Int. Ed. 2015, 54, 1651. 10.1002/anie.201410612 25487062

[253] B. J. Deibert , J. Li , Chem. Commun. 2014, 50, 9636. 10.1039/c4cc01938e 24801241

[254] J. Aguilera‐Sigalat , D. Bradshaw , Chem. Commun. 2014, 50, 4711. 10.1039/c4cc00659c 24675992

[255] Y. Xiao , Y. Cui , Q. Zheng , S. Xiang , G. Qian , B. Chen , Chem. Commun. 2010, 46, 5503. 10.1039/c0cc00148a 20593070

[256] C. Shao , Z. M. Su , Inorg. Chem. Commun. 2015, 57, 4.

[257] W. Dan , X. Liu , M. Deng , Y. Ling , Z. Chen , Y. Zhou , Dalton Trans. 2015, 44, 3794. 25609548 10.1039/c4dt03502j

[258] J.‐N. Hao , B. Yan , Chem. Commun. 2015, 51, 7737. 10.1039/c5cc01430a 25853430

[259] P. Wu , Y. Liu , Y. Liu , J. Wang , Y. Li , W. Liu , J. Wang , Inorg. Chem. 2015, 54, 11046. 26584394 10.1021/acs.inorgchem.5b01758

[260] W.‐Y. Li , S. Yang , Y.‐A. Li , Q.‐Y. Li , Q. Guan , Y.‐B. Dong , Dalton Trans. 2019, 48, 16502. 31528960 10.1039/c9dt02866h

[261] C. Liu , B. Yan , J. Colloid Interface Sci. 2015, 459, 206. 26298079 10.1016/j.jcis.2015.08.025

[262] Y. Zhou , H.‐H. Chen , B. Yan , J. Mater. Chem. A 2014, 2, 13691.

[263] Y. Gai , X. Zhao , Y. Chen , S. Yang , X. Xia , S. Liu , X. Wan , K. Xiong , Dalton Trans. 2018, 47, 6888. 29708255 10.1039/c8dt00979a

[264] Y.‐T. Liang , G.‐P. Yang , B. Liu , Y.‐T. Yan , Z.‐P. Xi , Y.‐Y. Wang , Dalton Trans. 2015, 44, 13325. 26133017 10.1039/c5dt01421b

[265] J. Zhang , L. Zhao , Y. Liu , M. Li , G. Li , X. Meng , New J. Chem. 2018, 42, 6839.

[266] X.‐Y. Xu , B. Yan , ACS Appl. Mater. Interfaces 2015, 7, 721. 25510710 10.1021/am5070409

[267] L.‐H. Cao , F. Shi , W.‐M. Zhang , S.‐Q. Zang , C. W. Mak Thomas , Chem. ‐ Eur. J. 2015, 21, 15705. 26493882 10.1002/chem.201501162

[268] T.‐B. Wei , X.‐Q. Ma , Y.‐Q. Fan , X.‐M. Jiang , H.‐Q. Dong , Q.‐Y. Yang , Y.‐F. Zhang , H. Yao , Q. Lin , Y.‐M. Zhang , Soft Matter 2019, 15, 6753. 31397832 10.1039/c9sm01385g

[269] F. Ming , J. Hou , D. Huo , J. Zhou , M. Yang , C. Shen , S. Zhang , C. Hou , Anal. Methods 2019, 11, 4382.

[270] W. Yang , J. Li , Z. Xu , J. Yang , Y. Liu , L. Liu , J. Mater. Chem. C 2019, 7, 10297.

[271] M. Huangfu , X. Tian , S. Zhao , P. Wu , H. Chu , X. Zheng , J. Tang , J. Wang , New J. Chem. 2019, 43, 10232.

[272] L. Deng , Y. Zhang , D. Zhang , S. Jiao , J. Xu , K. Liu , L. Wang , CrystEngComm 2019, 21, 6056.

[273] Z. Wang , J. Yang , Y. Li , Q. Zhuang , J. Gu , Dalton Trans. 2018, 47, 5570. 29632925 10.1039/c8dt00569a

[274] Y. Liu , M. Huangfu , P. Wu , M. Jiang , X. Zhao , L. Liang , L. Xie , J. Bai , J. Wang , Dalton Trans. 2019, 48, 13834. 31482925 10.1039/c9dt02962a

[275] Q.‐Q. Liu , S.‐H. Zhang , J. Yang , K.‐F. Yue , Analyst 2019, 144, 4534. 31268081 10.1039/c9an00858f

[276] D.‐D. Feng , Y.‐D. Zhao , X.‐Q. Wang , D.‐D. Fang , J. Tang , L.‐M. Fan , J. Yang , Dalton Trans. 2019, 48, 10892. 31111141 10.1039/c9dt01430f

[277] K.‐L. Wong , G.‐L. Law , Y.‐Y. Yang , W.‐T. Wong , Adv. Mater. 2006, 18, 1051.

[278] Y. Qiu , Z. Liu , Y. Li , H. Deng , R. Zeng , M. Zeller , Inorg. Chem. 2008, 47, 5122. 18470985 10.1021/ic702366t

[279] Y. Qiu , H. Deng , J. Mou , S. Yang , M. Zeller , S. R. Batten , H. Wu , J. Li , Chem. Commun. 2009, 5415. 10.1039/b907783a 19724803

[280] B. Chen , L. Wang , F. Zapata , G. Qian , E. B. Lobkovsky , J. Am. Chem. Soc. 2008, 130, 6718. 18452294 10.1021/ja802035e

[281] J. Sun , P. Guo , M. Liu , H. Li , J. Mater. Chem. C 2019, 7, 8992.

[282] A.‐M. Zhou , H. Wei , W. Gao , J.‐P. Liu , X.‐M. Zhang , CrystEngComm 2019, 21, 5185.

[283] B.‐W. Qin , X.‐Y. Zhang , J.‐P. Zhang , New J. Chem. 2019, 43, 13794.

[284] J. Zhang , W. Jia , J. Wu , G. Tang , C. Zhang , New J. Chem. 2019, 43, 16078.

[285] L.‐N. Wang , Y.‐H. Zhang , S. Jiang , Z.‐Z. Liu , CrystEngComm 2019, 21, 4557.

[286] F. Guo , C. Su , Y. Fan , W. Shi , Dalton Trans. 2019, 48, 12910. 31389461 10.1039/c9dt02921d

[287] J.‐J. Ma , W.‐s. Liu , Dalton Trans. 2019, 48, 12287. 31342032 10.1039/c9dt01907c

[288] C. Fan , Z. Zong , X. Meng , X. Zhang , X. Zhang , D. Zhang , C. Xu , H. Wang , Y. Fan , CrystEngComm 2019, 21, 4880.

[289] C. Gogoi , H. Reinsch , S. Biswas , CrystEngComm 2019, 21, 6252.

[290] Z. Zhan , Y. Jia , D. Li , X. Zhang , M. Hu , Dalton Trans. 2019, 48, 15255. 31580347 10.1039/c9dt03318a

[291] A. D. Pournara , A. Margariti , G. D. Tarlas , A. Kourtelaris , V. Petkov , C. Kokkinos , A. Economou , G. S. Papaefstathiou , M. J. Manos , J. Mater. Chem. A 2019, 7, 15432.

[292] D. Masih , V. Chernikova , O. Shekhah , M. Eddaoudi , O. F. Mohammed , ACS Appl. Mater. Interfaces 2018, 10, 11399. 29578682 10.1021/acsami.7b19282

[293] X. Sun , S. Yao , C. Yu , G. Li , C. Liu , Q. Huo , Y. Liu , J. Mater. Chem. A 2018, 6, 6363.

[294] Y. Wang , Y.‐C. He , F.‐H. Zhao , K. Zhu , J. Li , W.‐Q. Kan , Z. Jing , J. You , New J. Chem. 2019, 43, 13635.

[295] T. He , Y.‐Z. Zhang , X.‐J. Kong , J. Yu , X.‐L. Lv , Y. Wu , Z.‐J. Guo , J.‐R. Li , ACS Appl. Mater. Interfaces 2018, 10, 16650. 29733570 10.1021/acsami.8b03987

[296] Y. Shi , T.‐Q. Song , C.‐S. Cao , B. Zhao , CrystEngComm 2018, 20, 6040.

[297] Y.‐T. Yan , F. Cao , W.‐Y. Zhang , S.‐S. Zhang , F. Zhang , Y.‐Y. Wang , New J. Chem. 2018, 42, 9865.

[298] M. Zhang , Y. Zheng , M. Liu , Y. Ren , Z. Wang , J. Cao , J. Wang , RSC Adv. 2019, 9, 21086. 10.1039/c9ra03822a PMC9065990 35521299

[299] B. Joarder , V. Desai Aamod , P. Samanta , S. Mukherjee , K. Ghosh Sujit , Chem. ‐ Eur. J. 2014, 21, 965. 25424400 10.1002/chem.201405167

[300] S. S. Nagarkar , A. V. Desai , P. Samanta , S. K. Ghosh , Dalton Trans. 2015, 44, 15175. 25797881 10.1039/c5dt00397k

[301] J. Qin , B. Ma , X.‐F. Liu , H.‐L. Lu , X.‐Y. Dong , S.‐Q. Zang , H. Hou , J. Mater. Chem. A 2015, 3, 12690.

[302] Y. Guo , X. Feng , T. Han , S. Wang , Z. Lin , Y. Dong , B. Wang , J. Am. Chem. Soc. 2014, 136, 15485. 25325884 10.1021/ja508962m

[303] Y.‐X. Shi , F.‐L. Hu , W.‐H. Zhang , J.‐P. Lang , CrystEngComm 2015, 17, 9404.

[304] E. Zhou , Y. Zhang , Y. Li , X. He , Electroanalysis 2014, 26, 2526.

[305] J. Cui , Y.‐L. Wong , M. Zeller , D. Hunter Allen , Z. Xu , Angew. Chem., Int. Ed. 2014, 53, 14438. 10.1002/anie.201408453 25367865

[306] Y. Rachuri , B. Parmar , E. Suresh , Cryst. Growth Des. 2018, 18, 3062.

[307] X.‐D. Zhu , K. Zhang , Y. Wang , W.‐W. Long , R.‐J. Sa , T.‐F. Liu , J. Lü , Inorg. Chem. 2018, 57, 1060. 29308896 10.1021/acs.inorgchem.7b02471

[308] Y.‐M. Ying , C.‐L. Tao , M. Yu , Y. Xiong , C.‐R. Guo , X.‐G. Liu , Z. Zhao , J. Mater. Chem. C 2019, 7, 8383.

[309] Z. Zhou , M.‐L. Han , H.‐R. Fu , L.‐F. Ma , F. Luo , D.‐S. Li , Dalton Trans. 2018, 47, 5359. 29589624 10.1039/c8dt00594j

[310] J.‐K. Chen , S.‐M. Yang , B.‐H. Li , C.‐H. Lin , S. Lee , Langmuir 2018, 34, 1441. 29307176 10.1021/acs.langmuir.7b04240

[311] J.‐N. Hao , B. Yan , Chem. Commun. 2015, 51, 14509. 10.1039/c5cc05219j 26280941

[312] N. Li , D. Wu , N. Hu , G. Fan , X. Li , J. Sun , X. Chen , Y. Suo , G. Li , Y. Wu , J. Agric. Food Chem. 2018, 66, 3572. 29554797 10.1021/acs.jafc.8b00869

[313] D. Tian , X.‐J. Liu , R. Feng , J.‐L. Xu , J. Xu , R.‐Y. Chen , L. Huang , X.‐H. Bu , ACS Appl. Mater. Interfaces 2018, 10, 5618. 29350525 10.1021/acsami.7b15764

[314] W. Du , Z. Zhu , Y.‐L. Bai , Z. Yang , S. Zhu , J. Xu , Z. Xie , J. Fang , Chem. Commun. 2018, 54, 5972. 10.1039/c8cc02193g 29789816

[315] X.‐S. Zeng , H.‐L. Xu , Y.‐C. Xu , X.‐Q. Li , Z.‐Y. Nie , S.‐Z. Gao , D.‐R. Xiao , Inorg. Chem. Front. 2018, 5, 1622.

[316] X. Sun , X. Li , S. Yao , R. Krishna , J. Gu , G. Li , Y. Liu , J. Mater. Chem. A 2020, 8, 17106.

[317] J. Tao , X. Wang , T. Sun , H. Cai , Y. Wang , T. Lin , D. Fu , L. Ting , Y. Gu , D. Zhao , Sci. Rep. 2017, 7, 41640. 28139714 10.1038/srep41640 PMC5282571

[318] H.‐R. Fu , L.‐B. Yan , N.‐T. Wu , L.‐F. Ma , S.‐Q. Zang , J. Mater. Chem. A 2018, 6, 9183.

[319] T. Gong , P. Li , Q. Sui , J. Chen , J. Xu , E.‐Q. Gao , J. Mater. Chem. A 2018, 6, 9236.

[320] C. Zhuo , F. Wang , J. Zhang , CrystEngComm 2018, 20, 5925.

[321] C. Bao , Q. Niu , Z.‐A. Chen , X. Cao , H. Wang , W. Lu , RSC Adv. 2019, 9, 29474. 10.1039/c9ra05716a PMC9071849 35528419

[322] S. Gao , L. Zhao , L. Han , Z. Zhang , H. Zhao , CrystEngComm 2018, 20, 2237.

[323] S. Nandi , S. Biswas , Dalton Trans. 2019, 48, 9266. 31162519 10.1039/c9dt01180c

[324] M. Aghayan , A. Mahmoudi , S. Sohrabi , S. Dehghanpour , K. Nazari , N. Mohammadian‐Tabrizi , Anal. Methods 2019, 11, 3175.

[325] M. Z. Wu , J. Y. Shi , P. Y. Chen , L. Tian , New J. Chem. 2019, 43, 10575.

[326] S. M. Sheta , S. M. El‐Sheikh , M. M. Abd‐Elzaher , M. L. Ghanem , S. R. Salem , RSC Adv. 2019, 9, 20463. 10.1039/c9ra03030a PMC9065451 35514688

[327] X.‐M. Du , Q. Wang , Q. Liu , D. Ning , B. Zhao , Y. Li , W.‐J. Ruan , J. Mater. Chem. C 2019, 7, 8626.

[328] H. Li , J. Ren , X. Xu , L. Ning , R. Tong , Y. Song , S. Liao , W. Gu , X. Liu , Analyst 2019, 144, 4513. 31241067 10.1039/c9an00718k

[329] B.‐H. Wang , B. Yan , CrystEngComm 2019, 21, 4637.

[330] K. Ye , L. Wang , H. Song , X. Li , X. Niu , J. Mater. Chem. B 2019, 7, 4794. 31389965 10.1039/c9tb00951e

[331] C. Liu , T. Wang , J. Ji , C. Wang , H. Wang , P. Jin , W. Zhou , J. Jiang , J. Mater. Chem. C 2019, 7, 10240.

[332] M. S. Khan , M. Khalid , M. S. Ahmad , M. Shahid , M. Ahmad , Dalton Trans. 2019, 48, 12918. 31389450 10.1039/c9dt02578b

[333] J. Zeng , X. Ding , L. Chen , L. Jiao , Y. Wang , C. D. Windle , Q. Han , L. Qu , RSC Adv. 2019, 9, 28207. 10.1039/c9ra03802g PMC9071006 35530476

[334] J. Zeng , R. Xu , L. Jiao , Y. Wang , L. Chen , C. D. Windle , X. Ding , Z. Zhang , Q. Han , L. Qu , J. Mater. Chem. B 2019, 7, 5291. 31464334 10.1039/c9tb01223k

[335] Q. Liu , H. Wang , P. Han , X. Feng , Analyst 2019, 144, 6025. 31501829 10.1039/c9an00943d

[336] J.‐M. Liu , J.‐X. Hou , J. Liu , X. Jing , L.‐J. Li , J.‐L. Du , J. Mater. Chem. C 2019, 7, 11851.

[337] Q.‐Q. Liu , K. Yue , X.‐j. Weng , Y.‐Y. Wang , CrystEngComm 2019, 21, 6186.

[338] P. Verma , U. P. Singh , R. J. Butcher , CrystEngComm 2019, 21, 5470.

[339] M.‐J. Li , H.‐J. Wang , R. Yuan , Y.‐Q. Chai , Chem. Commun. 2019, 55, 10772. 10.1039/c9cc05086h 31432820

[340] M. Gong , J. Yang , Y. Li , Q. Zhuang , J. Gu , J. Mater. Chem. C 2019, 7, 12674.

[341] C. Li , L. Lu , J. Wang , Q. Yang , D. Ma , A. Alowais , A. Alarifi , A. Kumar , M. Muddassir , RSC Adv. 2019, 9, 29864. 10.1039/c9ra05167h PMC9071905 35531542

[342] R. Cui , Y. Wan , G. Ji , Z. Liu , Analyst 2019, 144, 5875. 31486467 10.1039/c9an01204d

[343] J. Gao , Y. Chen , W. Ji , Z. Gao , J. Zhang , Analyst 2019, 144, 6617. 31617506 10.1039/c9an01606f

[344] Y. Hua , Y. Cai , H. Liu , Y. Wan , X. Ding , S. Li , H. Wang , Nanoscale 2019, 11, 17401. 31528930 10.1039/c9nr05681e

[345] X. Zheng , R. Fan , H. Lu , B. Wang , J. Wu , P. Wang , Y. Yang , Dalton Trans. 2019, 48, 14408. 31509135 10.1039/c9dt02643f

[346] P. F. Muldoon , G. Collet , S. V. Eliseeva , T. Y. Luo , S. Petoud , N. L. Rosi , J. Am. Chem. Soc. 2020, 142, 8776. 32311264 10.1021/jacs.0c01426

[347] L. Liu , X. Chen , J. Qiu , C. Hao , Dalton Trans. 2015, 44, 2897. 25563388 10.1039/c4dt03185g

[348] L. Liu , J. Hao , Y. Shi , J. Qiu , C. Hao , RSC Adv. 2015, 5, 3045.

[349] Y.‐Y. Liu , R. Decadt , T. Bogaerts , K. Hemelsoet , A. M. Kaczmarek , D. Poelman , M. Waroquier , V. Van Speybroeck , R. Van Deun , P. Van Der Voort , J. Phys. Chem. C 2013, 117, 11302.

[350] Q. Li , X. Wu , X. Huang , Y. Deng , N. Chen , D. Jiang , L. Zhao , Z. Lin , Y. Zhao , ACS Appl. Mater. Interfaces 2018, 10, 3801. 29323480 10.1021/acsami.7b17762

[351] J. Dong , C. Tan , K. Zhang , Y. Liu , P. J. Low , J. W. Jiang , Y. Cui , J. Am. Chem. Soc. 2017, 139, 1554. 28059502 10.1021/jacs.6b11422

[352] J. Dong , K. Zhang , X. Li , Y. Qian , H. Zhu , D. Yuan , Q. H. Xu , J. W. Jiang , D. Zhao , Nat. Commun. 2017, 8, 1142. 29070835 10.1038/s41467-017-01293-x PMC5656651

[353] J. Dong , X. Li , S. B. Peh , Y. D. Yuan , Y. Wang , D. Ji , S. Peng , G. Liu , S. Ying , D. Yuan , J. W. Jiang , S. Ramakrishna , D. Zhao , Chem. Mater. 2019, 31, 146.

[354] J. Dong , Y. Pan , H. Wang , K. Yang , L. Liu , Z. Qiao , Y. D. Yuan , S. B. Pen , J. Zhang , L. Shi , H. Liang , Y. Han , X. Li , J. W. Jiang , B. Liu , D. Zhao , Angew. Chem., Int. Ed. 2020, 59, 10151. 10.1002/anie.201915199 31859381

[355] Y. J. Cui , D. Yue , Y. Huang , J. Zhang , Z. Wang , D. Yang , G. Qian , Chem. Commun. 2019, 55, 11231. 10.1039/c9cc05019a 31469133

[356] Y. Tang , H. Huang , B. Peng , Y. Chang , Y. Li , C. Zhong , J. Mater. Chem. A 2020, 8, 16542.

[357] M. Lammerhofer , J. Chromatogr. A 2010, 1217, 814. 19906381 10.1016/j.chroma.2009.10.022

[358] C. L. Chang , X. Wang , Y. Bai , H. W. Liu , Trends Anal. Chem. 2012, 39, 195.

[359] J. S. Seo , D. Whang , H. Lee , S. I. Jun , J. Oh , Y. J. Jeon , K. Kim , Nature 2000, 404, 982. 10801124 10.1038/35010088

[360] O. R. Evans , H. L. Ngo , W. B. Lin , J. Am. Chem. Soc. 2001, 123, 10395. 11603994 10.1021/ja0163772

[361] R. G. Xiong , X. Z. You , B. F. Abrahams , Z. L. Xue , C. M. Che , Angew. Chem., Int. Ed. 2001, 40, 4422. 10.1002/1521-3773(20011203)40:23<4422::aid-anie4422>3.0.co;2-g 12404434

[362] Y. R. Xie , X. S. Wang , H. Zhao , J. Zhang , L. H. Weng , C. Y. Duan , R. G. Xiong , X. Z. You , Z. L. Xue , Organometallics 2003, 22, 4396.

[363] D. Bradshaw , T. J. Prior , E. J. Cussen , J. B. Claridge , M. J. Rosseinsky , J. Am. Chem. Soc. 2004, 126, 6106. 15137776 10.1021/ja0316420

[364] C. D. Wu , W. Lin , Chem. Commun. 2005, 41, 3673. 10.1039/b505916j 16027907

[365] R. Vaidhyanathan , D. Bradshaw , J. N. Rebilly , J. P. Barrio , J. A. Gould , N. G. Berry , M. J. Rosseinsky , Angew. Chem., Int. Ed. 2006, 45, 6495. 10.1002/anie.200602242 16960821

[366] Y. M. Song , T. Zhou , X. S. Wang , X. N. Li , R. G. Xiong , Cryst. Growth Des. 2006, 6, 14.

[367] D. N. Dybtsev , A. L. Nuzhdin , H. Chun , K. P. Bryliakov , E. P. Talsi , V. P. Fedin , K. Kim , Angew. Chem., Int. Ed. 2006, 45, 916. 10.1002/anie.200503023 16385607

[368] A. L. Nuzhdin , D. N. Dybtsev , K. P. Bryliakov , E. P. Talsi , V. P. Fedin , J. Am. Chem. Soc. 2007, 129, 12958. 17924635 10.1021/ja076276p

[369] D. N. Dybtsev , M. P. Yutkin , D. G. Samsonenko , V. P. Fedin , A. L. Nuzhdin , A. A. Bezrukov , K. P. Bryliakov , E. P. Talsi , R. V. Belosludov , H. Mizuseki , Y. Kawazoe , O. S. Subbotin , V. R. Belosludov , Chem. ‐ Eur. J. 2010, 16, 10348. 20730747 10.1002/chem.201000522

[370] K. Suh , M. P. Yutkin , D. N. Dybtsev , V. P. Fedin , K. Kim , Chem. Commun. 2012, 48, 513. 10.1039/c1cc16209h 22127026

[371] G. Li , W. B. Yu , J. Ni , T. F. Liu , Y. Liu , E. H. Sheng , Y. Cui , Angew. Chem., Int. Ed. 2008, 47, 1245. 10.1002/anie.200704347 18176925

[372] G. Li , W. B. Yu , Y. Cui , J. Am. Chem. Soc. 2008, 130, 4582. 18345674 10.1021/ja078317n

[373] G. Yuan , C. F. Zhu , W. M. Xuan , Y. Cui , Chem. ‐ Eur. J. 2009, 15, 6428. 19462387 10.1002/chem.200900037

[374] T. F. Liu , Y. Liu , W. M. Xuan , Y. Cui , Angew. Chem., Int. Ed. 2010, 49, 4121. 10.1002/anie.201000416 20458719

[375] S. C. Xiang , Z. J. Zhang , C. G. Zhao , K. L. Hong , X. B. Zhao , D. R. Ding , M. H. Xie , C. D. Wu , M. C. Das , R. Gill , K. M. Thomas , B. L. Chen , Nat. Commun. 2011, 2, 204. 21343922 10.1038/ncomms1206

[376] K. Tanaka , T. Muraoka , D. Hirayama , A. Ohnish , Chem. Commun. 2012, 48, 8577. 10.1039/c2cc33939k 22806303

[377] B. Liu , O. Shekhah , H. K. Arslan , J. X. Liu , C. Woll , R. A. Fischer , Angew. Chem., Int. Ed. 2012, 51, 807. 10.1002/anie.201104240 22052527

[378] M. Zhang , Z. J. Pu , X. L. Chen , X. L. Gong , A. X. Zhu , L. M. Yuan , Chem. Commun. 2013, 49, 5201. 10.1039/c3cc41966e 23632942

[379] P. Li , Y. B. He , J. Guang , L. H. Weng , J. C. G. Zhao , S. C. Xiang , B. L. Chen , J. Am. Chem. Soc. 2014, 136, 547. 24392725 10.1021/ja4129795

[380] M. Padmanaban , P. Muller , C. Lieder , K. Gedrich , R. Grunker , V. Bon , I. Senkovska , S. Baumgartner , S. Opelt , S. Paasch , E. Brunner , F. Glorius , E. Klemm , S. Kaskel , Chem. Commun. 2011, 47, 12089. 10.1039/c1cc14893a 21960280

[381] M. Zhang , X. D. Xue , J. H. Zhang , S. M. Xie , Y. Zhang , L. M. Yuan , Anal. Methods 2014, 6, 341.

[382] M. Zhang , J. H. Zhang , Y. Zhang , B. J. Wang , S. M. Xie , L. M. Yuan , J. Chromatogr. A 2014, 1325, 163. 24373537 10.1016/j.chroma.2013.12.023

[383] J. Kong , M. Zhang , A. H. Duan , J. H. Zhang , R. Yang , L. M. Yuan , J. Sep. Sci. 2015, 38, 556. 25491646 10.1002/jssc.201401034

[384] X. Kuang , Y. Ma , H. Su , J. Zhang , Y. B. Dong , B. Tang , Anal. Chem. 2014, 86, 1277. 24380495 10.1021/ac403674p

[385] C. Kutzscher , H. C. Hoffmann , S. Krause , U. Stoeck , I. Senkovska , E. Brunner , S. Kaskel , Inorg. Chem. 2015, 54, 1003. 25490603 10.1021/ic502380q

[386] R. Hailili , L. Wang , J. Qv , R. Yao , X. M. Zhang , H. Liu , Inorg. Chem. 2015, 54, 3713. 25849610 10.1021/ic502861k

[387] M. Zhang , X. Chen , J. Zhang , J. Kong , L. Yuan , Chirality 2016, 28, 340. 26901397 10.1002/chir.22588

[388] K. Tanaka , T. Muraoka , Y. Otubo , H. Takahashi , A. Ohnishi , RSC Adv. 2016, 6, 21293.

[389] K. J. Hartlieb , J. M. Holcroft , P. Z. Moghadam , N. A. Vermeulen , M. M. Algaradah , M. S. Nassar , Y. Y. Botros , R. Q. Snurr , J. F. Stoddart , J. Am. Chem. Soc. 2016, 138, 2292. 26812983 10.1021/jacs.5b12860

[390] K. Huang , X. L. Dong , R. F. Ren , W. Q. Jin , AIChE J. 2013, 59, 4364.

[391] Z. X. Kang , M. Xue , L. L. Fan , J. Y. Ding , L. J. Guo , L. X. Gao , S. L. Qiu , Chem. Commun. 2013, 49, 10569. 10.1039/c3cc42376j 23792620

[392] J. Zhang , Z. Li , W. Gong , X. Han , Y. Liu , Y. Cui , Inorg. Chem. 2016, 55, 7229. 27227785 10.1021/acs.inorgchem.6b00894

[393] J. H. Zhang , R. Y. Nong , S. M. Xie , B. J. Wang , P. Ai , L. M. Yuan , Electrophoresis 2017, 38, 2513. 28678407 10.1002/elps.201700122

[394] B. Slater , Z. Wang , S. Jiang , M. R. Hill , B. P. Ladewig , J. Am. Chem. Soc. 2017, 139, 18322. 29179533 10.1021/jacs.7b10112

[395] A. Abbas , Z.‐X. Wang , Z. Li , H. Jiang , Y. Liu , Y. Cui , Inorg. Chem. 2018, 57, 8697. 30016086 10.1021/acs.inorgchem.8b00948

[396] X. Han , J. Huang , C. Yuan , Y. Liu , Y. Cui , J. Am. Chem. Soc. 2018, 140, 892. 29302963 10.1021/jacs.7b12110

[397] W. J. Wang , X. L. Dong , J. P. Nan , W. Q. Jin , Z. Q. Hu , Y. F. Chen , J. W. Jiang , Chem. Commun. 2012, 48, 7022. 10.1039/c2cc32595k 22575898

[398] X. Y. Bao , L. J. Broadbelt , R. Q. Snurr , Mol. Simul. 2009, 35, 50.

[399] X. Y. Bao , L. J. Broadbelt , R. Q. Snurr , Microporous Mesoporous Mater. 2011, 157, 118.

[400] J. Navarro‐Sánchez , A. I. Argente‐García , Y. Moliner‐Martínez , D. Roca‐Sanjuán , D. Antypov , P. Campíns‐Falcó , M. J. Rosseinsky , C. Martí‐Gastaldo , J. Am. Chem. Soc. 2017, 139, 4294. 28274119 10.1021/jacs.7b00280

[401] Y. Peng , T. Gong , K. Zhang , X. Lin , Y. Liu , J. W. Jiang , Y. Cui , Nat. Commun. 2014, 5, 4406. 25030529 10.1038/ncomms5406

[402] P. Horcajada , R. Gref , T. Baati , P. K. Allan , G. Maurin , P. Couvreur , G. Ferey , C. Serre , Chem. Rev. 2012, 112, 1232. 22168547 10.1021/cr200256v

[403] P. Horcajada , C. Serre , M. Vallet‐Regi , M. Sebban , F. Taulelle , G. Ferey , Angew. Chem., Int. Ed. 2006, 45, 5974. 10.1002/anie.200601878 16897793

[404] P. Horcajada , C. Serre , G. Maurin , N. A. Ramsahye , F. Balas , M. Vallet‐Regi , M. Sebban , F. Taulelle , G. Ferey , J. Am. Chem. Soc. 2008, 130, 6774. 18454528 10.1021/ja710973k

[405] S. R. Miller , D. Heurtaux , T. Baati , P. Horcajada , J. M. Greneche , C. Serre , Chem. Commun. 2010, 46, 4526. 10.1039/c001181a 20467672

[406] P. Horcajada , T. Chalati , C. Serre , B. Gillet , C. Sebrie , T. Baati , J. F. Eubank , D. Heurtaux , P. Clayette , C. Kreuz , J.‐S. Chang , Y. K. Hwang , V. Marsaud , P.‐N. Bories , L. Cynober , S. Gil , G. Ferey , P. Couvreur , R. Gref , Nat. Mater. 2010, 9, 172. 20010827 10.1038/nmat2608

[407] T. Chalati , P. Horcajada , P. Couvreur , C. Serre , M. Ben Yahia , G. Maurin , R. Gref , Nanomedicine 2011, 6, 1683. 22122581 10.2217/nnm.11.69

[408] C. Gaudin , D. Cunha , E. Ivanoff , P. Horcajada , G. Cheve , A. Yasri , O. Loget , C. Serre , G. Maurin , Microporous Mesoporous Mater. 2012, 157, 124.

[409] N. Liedana , P. Lozano , A. Galve , C. Tellez , J. Coronas , J. Mater. Chem. B 2014, 2, 1144. 32261350 10.1039/c3tb21707h

[410] D. Cunha , C. Gaudin , I. Colinet , P. Horcajada , C. Serre , G. Maurin , J. Mater. Chem. B 2013, 1, 1101. 32260833 10.1039/c2tb00366j

[411] I. Imaz , M. Rubio‐Martinez , L. Garcia‐Fernandez , F. Garcia , D. Ruiz‐Molina , J. Hernando , V. Puntes , D. Maspoch , Chem. Commun. 2010, 46, 4737. 10.1039/c003084h 20485835

[412] F. Ke , Y.‐P. Yuan , L.‐G. Qiu , Y.‐H. Shen , A.‐J. Xie , J.‐F. Zhu , X.‐Y. Tian , L.‐D. Zhang , J. Mater. Chem. 2011, 21, 3843.

[413] A. D. Burrows , M. Jurcic , L. L. Keenan , R. A. Lane , M. F. Mahon , M. R. Warren , H. Nowell , M. Paradowskic , J. Spencer , Chem. Commun. 2013, 49, 11260. 10.1039/c3cc45689g 24135827

[414] T. Cendak , E. Zunkovic , T. U. Godec , M. Mazaj , N. Z. Logar , G. Mali , J. Phys. Chem. C 2014, 118, 6140.

[415] J. Zhuang , C. H. Kuo , L. Y. Chou , D. Y. Liu , E. Weerapana , C. K. Tsung , ACS Nano 2014, 8, 2812. 24506773 10.1021/nn406590q

[416] X. Y. Zhu , J. L. Gu , Y. Wang , B. Li , Y. S. Li , W. R. Zhao , J. L. Shi , Chem. Commun. 2014, 50, 8779. 10.1039/c4cc02570a 24967656

[417] R. Anand , F. Borghi , F. Manoli , I. Manet , V. Agostoni , P. Reschiglian , R. Gref , S. Monti , J. Phys. Chem. B 2014, 118, 8532. 24960194 10.1021/jp503809w

[418] Y. A. Li , X. D. Zhao , H. P. Yin , G. J. Chen , S. Yang , Y. B. Dong , Chem. Commun. 2016, 52, 14113. 10.1039/c6cc07321b 27858003

[419] W. J. Rieter , K. M. Pott , K. M. L. Taylor , W. B. Lin , J. Am. Chem. Soc. 2008, 130, 11584. 18686947 10.1021/ja803383k

[420] K. M. L. Taylor‐Pashow , J. D. Rocca , Z. Xie , S. Tran , W. B. Lin , J. Am. Chem. Soc. 2009, 131, 14261. 19807179 10.1021/ja906198y PMC2760011

[421] D. M. Liu , S. A. Kramer , R. C. Huxford‐Phillips , S. Z. Wang , J. D. Rocca , W. B. Lin , Chem. Commun. 2012, 48, 2668. 10.1039/c2cc17635a PMC3778684 22428170

[422] W. Cullen , S. Turega , C. A. Hunter , M. D. Ward , Chem. Sci. 2015, 6, 625. 28936311 10.1039/c4sc02090a PMC5588781

[423] L. Cooper , T. Hidalgo , M. Gorman , T. Lozano‐Fernández , R. Simón‐Vázquez , C. Olivier , N. Guillou , C. Serre , C. Martineau , F. Taulelle , D. Damasceno‐Borges , G. Maurin , A. González‐Fernández , P. Horcajada , T. Devic , Chem. Commun. 2015, 51, 5848. 10.1039/c5cc00745c 25720815

[424] L. L. Tan , H. Li , Y. C. Qiu , D. X. Chen , X. Wang , R. Y. Pan , Y. Wang , S. X. A. Zhang , B. Wang , Y. W. Yang , Chem. Sci. 2015, 6, 1640. 30154997 10.1039/c4sc03749a PMC6085730

[425] S. Nagata , K. Kokado , K. Sada , Chem. Commun. 2015, 51, 8614. 10.1039/c5cc02339d 25896867

[426] J. An , S. J. Geib , N. L. Rosi , J. Am. Chem. Soc. 2009, 131, 8376. 19489551 10.1021/ja902972w

[427] S. Wuttke , S. Braig , T. Preiß , A. Zimpel , J. Sicklinger , C. Bellomo , J. O. Rädler , A. M. Vollmar , T. Bein , Chem. Commun. 2015, 51, 15752. 10.1039/c5cc06767g 26359316

[428] C. Orellana‐Tavra , E. F. Baxter , T. Tian , T. D. Bennett , N. K. H. Slater , A. K. Cheetham , D. Fairen‐Jimenez , Chem. Commun. 2015, 51, 13878. 10.1039/c5cc05237h 26213904

[429] M. R. Lohe , K. Gedrich , T. Freudenberg , E. Kockrick , T. Dellmann , S. Kaskel , Chem. Commun. 2011, 47, 3075. 10.1039/c0cc05278g 21293827

[430] Q. Fang , J. Wang , S. Gu , R. B. Kaspar , Z. Zhuang , J. Zheng , H. Guo , S. Qiu , Y. Yan , J. Am. Chem. Soc. 2015, 137, 8352. 26099722 10.1021/jacs.5b04147

[431] A. B. Lago , A. Pino‐Cuevas , R. Carballo , E. M. Vázquez‐López , Dalton Trans. 2016, 45, 1614. 26692254 10.1039/c5dt04031k

[432] H. Zhao , Z. Jin , H. Su , X. Jing , F. Sun , G. Zhu , Chem. Commun. 2011, 47, 6389. 10.1039/c1cc00084e 21552587

[433] D. Zhao , S. W. Tan , D. Q. Yuan , W. G. Lu , Y. H. Rezenom , H. L. Jiang , L. Q. Wang , H. C. Zhou , Adv. Mater. 2011, 23, 90. 20972982 10.1002/adma.201003012

[434] L. He , T. T. Wang , J. P. An , X. M. Li , L. Y. Zhang , L. Li , G. Z. Li , X. T. Wu , Z. M. Su , C. G. Wang , CrystEngComm 2014, 16, 3259.

[435] H. N. Wang , X. Meng , G. S. Yang , X. L. Wang , K. Z. Shao , Z. M. Su , C. G. Wang , Chem. Commun. 2011, 47, 7128. 10.1039/c1cc11932j 21614372

[436] R. Li , X. Q. Ren , J. S. Zhao , X. Feng , X. Jiang , X. X. Fan , Z. G. Lin , X. G. Li , C. W. Hu , B. Wang , J. Mater. Chem. A 2014, 2, 2168.

[437] J. S. Qin , D. Y. Du , W. L. Li , J. P. Zhang , S. L. Li , Z. M. Su , X. L. Wang , Q. Xu , K. Z. Shao , Y. Q. Lan , Chem. Sci. 2012, 3, 2114.

[438] J. Wang , J. Jin , F. Li , B. Li , J. Liu , J. Jin , C. Wang , Y. Zeng , Y. Wang , RSC Adv. 2015, 5, 85606.

[439] J. Q. Liu , X. F. Li , C. Y. Gu , J. C. S. Da Silva , A. L. Barros , S. Alves , B. H. Li , F. Ren , S. R. Batten , T. A. Soares , Dalton Trans. 2015, 44, 19370. 26501793 10.1039/c5dt02171e

[440] P. P. Bag , D. Wang , Z. Chen , R. Cao , Chem. Commun. 2016, 52, 3669. 10.1039/c5cc09925k 26853858

[441] C. Y. Sun , C. Qin , C. G. Wang , Z. M. Su , S. Wang , X. L. Wang , G. S. Yang , K. Z. Shao , Y. Q. Lan , E. B. Wang , Adv. Mater. 2011, 23, 5629. 22095878 10.1002/adma.201102538

[442] C. Y. Sun , C. Qin , X. L. Wang , G. S. Yang , K. Z. Shao , Y. Q. Lan , Z. M. Su , P. Huang , C. G. Wang , E. B. Wang , Dalton Trans. 2012, 41, 6906. 22580798 10.1039/c2dt30357d

[443] L. L. Tan , N. Song , S. X. A. Zhang , H. Li , B. Wang , Y. W. Yang , J. Mater. Chem. B 2016, 4, 135. 32262817 10.1039/c5tb01789k

[444] W. Li , Y. Zhang , Z. Xu , Q. Meng , Z. Fan , S. Ye , G. Zhang , Angew. Chem., Int. Ed. 2016, 55, 955. 10.1002/anie.201508795 26636438

[445] H. Wang , E. Lashkari , H. Lim , C. Zheng , T. J. Emge , Q. Gong , K. Yam , J. Li , Chem. Commun. 2016, 52, 2129. 10.1039/c5cc09634k 26696556

[446] L. Wang , W. Wang , Z. Xie , J. Mater. Chem. B 2016, 4, 4263. 32263407 10.1039/c6tb00952b

[447] A. G. Márquez , T. Hidalgo , H. Lana , D. Cunha , M. J. Blanco‐Prieto , C. Álvarez‐Lorenzo , C. Boissière , C. Sánchez , C. Serre , P. Horcajada , J. Mater. Chem. B 2016, 4, 7031. 32263570 10.1039/c6tb01652a

[448] C. Orellana‐Tavra , R. J. Marshall , E. F. Baxter , I. A. Lázaro , A. Tao , A. K. Cheetham , R. S. Forgan , D. Fairen‐Jimenez , J. Mater. Chem. B 2016, 4, 7697. 32263827 10.1039/c6tb02025a

[449] S. Mitra , H. S. Sasmal , T. Kundu , S. Kandambeth , K. Illath , D. Díaz Díaz , R. Banerjee , J. Am. Chem. Soc. 2017, 139, 4513. 28256830 10.1021/jacs.7b00925

[450] T. Hidalgo , L. Cooper , M. Gorman , T. Lozano‐Fernández , R. Simón‐Vázquez , G. Mouchaham , J. Marrot , N. Guillou , C. Serre , P. Fertey , Á. González‐Fernández , T. Devic , P. Horcajada , J. Mater. Chem. B 2017, 5, 2813. 32264168 10.1039/c6tb03101c

[451] M. H. Teplensky , M. Fantham , P. Li , T. C. Wang , J. P. Mehta , L. J. Young , P. Z. Moghadam , J. T. Hupp , O. K. Farha , C. F. Kaminski , D. Fairen‐Jimenez , J. Am. Chem. Soc. 2017, 139, 7522. 28508624 10.1021/jacs.7b01451

[452] K. J. Hartlieb , D. P. Ferris , J. M. Holcroft , I. Kandela , C. L. Stern , M. S. Nassar , Y. Y. Botros , J. F. Stoddart , Mol. Pharmaceutics 2017, 14, 1831. 10.1021/acs.molpharmaceut.7b00168 28355489

[453] B. Illes , P. Hirschle , S. Barnert , V. Cauda , S. Wuttke , H. Engelke , Chem. Mater. 2017, 29, 8042.

[454] Z. Dong , Y. Sun , J. Chu , X. Zhang , H. Deng , J. Am. Chem. Soc. 2017, 139, 14209. 28898070 10.1021/jacs.7b07392

[455] H. Li , N. Lv , X. Li , B. Liu , J. Feng , X. Ren , T. Guo , D. Chen , J. Fraser Stoddart , R. Gref , J. Zhang , Nanoscale 2017, 9, 7454. 28530283 10.1039/c6nr07593b

[456] T. Simon‐Yarza , M. Gimenez‐Marques , R. Mrimi , A. Mielcarek , R. Gref , P. Horcajada , C. Serre , P. Couvreur , Angew. Chem., Int. Ed. 2017, 56, 15565. 10.1002/anie.201707346 28960750

[457] B. Mandal , J. S. Chung , S. G. Kang , Phys. Chem. Chem. Phys. 2017, 19, 31316. 29148559 10.1039/c7cp04831a

[458] W. H. Chen , S. Yang Sung , M. Fadeev , A. Cecconello , R. Nechushtai , I. Willner , Nanoscale 2018, 10, 4650. 29465130 10.1039/c8nr00193f

[459] S. Peng , B. Bie , Y. Sun , M. Liu , H. Cong , W. Zhou , Y. Xia , H. Tang , H. Deng , X. Zhou , Nat. Commun. 2018, 9, 1293. 29615605 10.1038/s41467-018-03650-w PMC5882967

[460] Y. J. Chen , P. Li , J. A. Modica , R. J. Drout , O. K. Farha , J. Am. Chem. Soc. 2018, 140, 5678. 29641892 10.1021/jacs.8b02089

[461] G. Zhang , X. Li , Q. Liao , Y. Liu , K. Xi , W. Huang , X. Jia , Nat. Commun. 2018, 9, 2785. 30018290 10.1038/s41467-018-04910-5 PMC6050241

[462] I. Abánades Lázaro , S. Haddad , J. M. Rodrigo‐Muñoz , C. Orellana‐Tavra , V. del Pozo , D. Fairen‐Jimenez , R. S. Forgan , ACS Appl. Mater. Interfaces 2018, 10, 5255. 29356507 10.1021/acsami.7b17756

[463] L. Wang , H. Zhu , Y. Shi , Y. Ge , X. Feng , R. Liu , Y. Li , Y. Ma , L. Wang , Nanoscale 2018, 10, 11384. 29877544 10.1039/c8nr02493f

[464] H. Zhou , C. Fu , X. Chen , L. Tan , J. Yu , Q. Wu , L. Su , Z. Huang , F. Cao , X. Ren , J. Ren , P. Liang , X. Meng , Biomater. Sci. 2018, 6, 1535. 29670952 10.1039/c8bm00142a

[465] Y. Duan , F. Ye , Y. Huang , Y. Qin , C. He , S. Zhao , Chem. Commun. 2018, 54, 5377. 10.1039/c8cc02708k 29745409

[466] T. Baati , L. Njim , F. Neffati , A. Kerkeni , M. Bouttemi , R. Gref , M. F. Najjar , A. Zakhama , P. Couvreur , C. Serre , P. Horcajada , Chem. Sci. 2013, 4, 1597.

[467] R. Grall , T. Hidalgo , J. Delic , A. Garcia‐Marquez , S. Chevillard , P. Horcajada , J. Mater. Chem. B 2015, 3, 8279. 32262883 10.1039/c5tb01223f

[468] R. Babarao , J. W. Jiang , J. Phys. Chem. C 2009, 113, 18287. 10.1021/jp902253p 19534507

[469] M. O. Rodrigues , M. V. de Paula , K. A. Wanderley , I. B. Vasconcelos , S. Alves Jr. , T. A. Soares , Int. J. Quantum Chem. 2012, 112, 3346.

[470] D. Cunha , M. Ben Yahia , S. Hall , S. R. Miller , H. Chevreau , E. Elkaim , G. Maurin , P. Horcajada , C. Serre , Chem. Mater. 2013, 25, 2767.

[471] M. C. Bernini , D. Fairen‐Jimenez , M. Pasinetti , A. J. Ramirez‐Pastor , R. Q. Snurr , J. Mater. Chem. B 2014, 2, 766. 32261308 10.1039/c3tb21328e

[472] E. N. Koukaras , T. Montagnon , P. Trikalitis , D. Bikiaris , A. D. Zdetsis , G. E. Froudakis , J. Phys. Chem. C 2014, 118, 8885.

[473] B. Supronowicz , A. Mavrandonakis , T. Heine , J. Phys. Chem. C 2015, 119, 3024.

[474] I. Erucar , S. Keskin , Ind. Eng. Chem. Res. 2016, 55, 1929. 10.1021/acs.iecr.9b05487 PMC7076730 32201455

[475] I. Erucar , S. Keskin , J. Mater. Chem. B 2017, 5, 7342. 32264184 10.1039/c7tb01764b

[476] M. Kotzabasaki , I. Galdadas , E. Tylianakis , E. Klontzas , Z. Cournia , G. E. Froudakis , J. Mater. Chem. B 2017, 5, 3277. 32264393 10.1039/c7tb00220c

[477] M. Shahabi , H. Raissi , J. Mol. Model. 2019, 25, 304. 31493060 10.1007/s00894-019-4178-1

[478] V. Lykourinou , Y. Chen , X. S. Wang , L. Meng , T. Hoang , L. J. Ming , R. L. Musselman , S. Q. Ma , J. Am. Chem. Soc. 2011, 133, 10382. 21682253 10.1021/ja2038003

[479] Y. Chen , V. Lykourinou , C. Vetromile , T. Hoang , L. J. Ming , R. W. Larsen , S. Q. Ma , J. Am. Chem. Soc. 2012, 134, 13188. 22862180 10.1021/ja305144x

[480] G. Wang , Z. Xu , Z. Chen , W. Niu , Y. Zhou , J. Guo , L. Tan , Chem. Commun. 2013, 49, 6641. 10.1039/c3cc42711k 23770742

[481] W. Morris , W. E. Briley , E. Auyeung , M. D. Cabezas , C. A. Mirkin , J. Am. Chem. Soc. 2014, 136, 7261. 24818877 10.1021/ja503215w

[482] D. Rambabu , Pooja , C. P. Pradeep , A. Dhir , J. Mater. Chem. A 2014, 2, 8628.

[483] F. Lyu , Y. Zhang , R. N. Zare , J. Ge , Z. Liu , Nano Lett. 2014, 14, 5761. 25211437 10.1021/nl5026419

[484] Y. Jia , B. Wei , R. Duan , Y. Zhang , B. Wang , A. Hakeem , N. Liu , X. Ou , S. Xu , Z. Chen , X. Lou , F. Xia , Sci. Rep. 2014, 4, 1. 10.1038/srep05929 PMC4123201 25090047

[485] Y. Mao , J. Li , W. Cao , Y. Ying , P. Hu , Y. Liu , L. Sun , H. Wang , C. Jin , X. Peng , Nat. Commun. 2014, 5, 5532. 25405547 10.1038/ncomms6532

[486] D. Feng , T. F. Liu , J. Su , M. Bosch , Z. Wei , W. Wan , D. Yuan , Y. P. Chen , X. Wang , K. Wang , X. Lian , Z. Y. Gu , J. Park , X. Zou , H. C. Zhou , Nat. Commun. 2015, 6, 5979. 25598311 10.1038/ncomms6979

[487] H. X. Deng , S. Grunder , K. E. Cordova , C. Valente , H. Furukawa , M. Hmadeh , F. Gandara , A. C. Whalley , Z. Liu , S. Asahina , H. Kazumori , M. O'Keefe , O. Terasaki , J. F. Stoddart , O. M. Yaghi , Science 2012, 336, 1018. 22628651 10.1126/science.1220131

[488] F. K. Shieh , S. C. Wang , C. I. Yen , C. C. Wu , S. Dutta , L. Y. Chou , J. V. Morabito , P. Hu , M. H. Hsu , K. C. W. Wu , C. K. Tsung , J. Am. Chem. Soc. 2015, 137, 4276. 25781479 10.1021/ja513058h

[489] K. Liang , R. Ricco , C. M. Doherty , M. J. Styles , S. Bell , N. Kirby , S. Mudie , D. Haylock , A. J. Hill , C. J. Doonan , P. Falcaro , Nat. Commun. 2015, 6, 7240. 26041070 10.1038/ncomms8240 PMC4468859

[490] J. Bonnefoy , A. Legrand , E. A. Quadrelli , J. Canivet , D. Farrusseng , J. Am. Chem. Soc. 2015, 137, 9409. 26120932 10.1021/jacs.5b05327

[491] X. Wu , J. Ge , C. Yang , M. Hou , Z. Liu , Chem. Commun. 2015, 51, 13408. 10.1039/c5cc05136c 26214658

[492] K. Liang , C. J. Coghlan , S. G. Bell , C. Doonan , P. Falcaro , Chem. Commun. 2015, 52, 473. 10.1039/c5cc07577g 26548587

[493] J. Yang , X. Chen , Y. Li , Q. Zhuang , P. Liu , J. Gu , Chem. Mater. 2017, 29, 4580.

[494] F. S. Liao , W. S. Lo , Y. S. Hsu , C. C. Wu , S. C. Wang , F. K. Shieh , J. V. Morabito , L. Y. Chou , K. C. W. Wu , C. K. Tsung , J. Am. Chem. Soc. 2017, 139, 6530. 28460166 10.1021/jacs.7b01794

[495] M. Xu , S. Yuan , X. Y. Chen , Y. J. Chang , G. Day , Z. Y. Gu , H. C. Zhou , J. Am. Chem. Soc. 2017, 139, 8312. 28538098 10.1021/jacs.7b03450

[496] X. Qi , Z. Chang , D. Zhang , K. J. Binder , S. Shen , Y. Y. S. Huang , Y. Bai , A. E. H. Wheatley , H. Liu , Chem. Mater. 2017, 29, 8052.

[497] Y. Ikezoe , G. Washino , T. Uemura , S. Kitagawa , H. Matsui , Nat. Mater. 2012, 11, 1081. 23104155 10.1038/nmat3461 PMC3505225

[498] C. Liu , T. Li , N. L. Rosi , J. Am. Chem. Soc. 2012, 134, 18886. 23113571 10.1021/ja307713q

[499] X. Zhu , H. Zheng , X. Wei , Z. Lin , L. Guo , B. Qiu , G. Chen , Chem. Commun. 2013, 49, 1276. 10.1039/c2cc36661d 23295434

[500] Q. Sun , C. W. Fu , B. Aguila , J. Perman , S. Wang , H. Y. Huang , F. S. Xiao , S. Ma , J. Am. Chem. Soc. 2018, 140, 984. 29275637 10.1021/jacs.7b10642

[501] T.‐T. Chen , J.‐T. Yi , Y.‐Y. Zhao , X. Chu , J. Am. Chem. Soc. 2018, 140, 9912. 30008215 10.1021/jacs.8b04457

[502] C. Wang , H. Sun , J. Luan , Q. Jiang , S. Tadepalli , J. J. Morrissey , E. D. Kharasch , S. Singamaneni , Chem. Mater. 2018, 30, 1291.

[503] H. Yang , T. Li , L. Liu , N. Li , M. Guan , Y. Zhang , Z. Wang , Z. Zhao , Analyst 2018, 143, 2157. 29667690 10.1039/c8an00238j

[504] H. Li , X. Lu , Q. Lu , Y. Liu , X. Cao , Y. Lu , X. He , K. Chen , P. Ouyang , W. Tang , Chem. Commun. 2020, 56, 4724. 10.1039/d0cc00748j 32219295

[505] Z. Y. Gu , Y. J. Chen , J. Q. Jiang , X. P. Yan , Chem. Commun. 2011, 47, 4787. 10.1039/c1cc10579e 21399815

[506] C. B. Messner , M. R. Mirza , M. Rainer , O. M. D. Lutz , Y. Guzel , T. S. Hofer , C. W. Huck , B. M. Rode , G. K. HBonn , Anal. Methods 2013, 5, 2379.

[507] M. Zhao , C. Deng , X. Zhang , P. Yang , Proteomics 2013, 13, 3387. 24150872 10.1002/pmic.201300131

[508] Z. Xiong , Y. Ji , C. Fang , Q. Zhang , L. Zhang , M. Ye , W. Zhang , H. Zou , Chem. ‐ Eur. J. 2014, 20, 7389. 24807292 10.1002/chem.201400389

[509] M. Zhao , C. Deng , X. Zhang , Chem. Commun. 2014, 50, 6228. 10.1039/c4cc01038h 24789051

[510] Y. W. Zhang , Z. Li , Q. Zhao , Y. L. Zhou , H. W. Liu , X. X. Zhang , Chem. Commun. 2014, 50, 11504. 10.1039/c4cc05179c 25131456

[511] Y. Ji , Z. Xiong , G. Huang , J. Liu , Z. Zhang , Z. Liu , J. Ou , M. Ye , H. Zou , Analyst 2014, 139, 4987. 25110774 10.1039/c4an00971a

[512] J. Zheng , Z. Lin , G. Lin , H. Yang , L. Zhang , J. Mater. Chem. B 2015, 3, 2185. 32262386 10.1039/c4tb02007c

[513] J.‐P. Wei , B. Qiao , W.‐J. Song , T. Chen , F. li , B.‐Z. Li , J. Wang , Y. Han , Y.‐F. Huang , Z.‐J. Zhou , Anal. Chim. Acta 2015, 868, 36. 25813232 10.1016/j.aca.2015.02.018

[514] X. Zhu , J. Gu , J. Yang , Z. Wang , Y. Li , L. Zhao , W. Zhao , J. Shi , J. Mater. Chem. B 2015, 3, 4242. 32262301 10.1039/c5tb00113g

[515] Y. Chen , Z. Xiong , L. Peng , Y. Gan , Y. Zhao , J. Shen , J. Qian , L. Zhang , W. Zhang , ACS Appl. Mater. Interfaces 2015, 7, 16338. 26156207 10.1021/acsami.5b03335

[516] Y. Xie , C. Deng , Talanta 2016, 159, 1. 27474271 10.1016/j.talanta.2016.05.075

[517] J. Wang , J. Li , Y. Wang , M. Gao , X. Zhang , P. Yang , ACS Appl. Mater. Interfaces 2016, 8, 27482. 27681085 10.1021/acsami.6b08218

[518] W. Ma , L. Xu , Z. Li , Y. Sun , Y. Bai , H. Liu , Nanoscale 2016, 8, 10908. 27186633 10.1039/c6nr02490d

[519] G. Cheng , Z.‐G. Wang , S. Denagamage , S.‐Y. Zheng , ACS Appl. Mater. Interfaces 2016, 8, 10234. 27046460 10.1021/acsami.6b02209

[520] J.‐P. Wei , H. Wang , T. Luo , Z.‐J. Zhou , Y.‐F. Huang , B. Qiao , Anal. Bioanal. Chem. 2017, 409, 1895. 28012110 10.1007/s00216-016-0136-2

[521] M. Zhao , Y. Xie , H. Chen , C. Deng , Talanta 2017, 167, 392. 28340737 10.1016/j.talanta.2017.02.038

[522] W. Ma , L. Xu , X. Li , S. Shen , M. Wu , Y. Bai , H. Liu , ACS Appl. Mater. Interfaces 2017, 9, 19562. 28537384 10.1021/acsami.7b02853

[523] Y. Wang , J. Wang , M. Gao , X. Zhang , Proteomics 2017, 17, 1700005. 10.1002/pmic.201700005 28390088

[524] Q. Liu , Y. Xie , C. Deng , Y. Li , Talanta 2017, 175, 477. 28842020 10.1016/j.talanta.2017.07.067

[525] Y. Xie , C. Deng , Y. Li , J. Chromatogr. A 2017, 1508, 1. 28602507 10.1016/j.chroma.2017.05.055

[526] J. Peng , H. Zhang , X. Li , S. Liu , X. Zhao , J. Wu , X. Kang , H. Qin , Z. Pan , R. a. Wu , ACS Appl. Mater. Interfaces 2016, 8, 35012. 27983800 10.1021/acsami.6b12630

[527] G. Han , Q. Zeng , Z. Jiang , W. Deng , C. Huang , Y. Li , Talanta 2017, 171, 283. 28551142 10.1016/j.talanta.2017.03.106

[528] X. Yang , Y. Xia , Microchim. Acta 2016, 183, 2235.

[529] J. Li , J. Wang , Y. Ling , Z. Chen , M. Gao , X. Zhang , Y. Zhou , Chem. Commun. 2017, 53, 4018. 10.1039/c7cc00447h 28338695

[530] S. Peng , B. Bie , H. Jia , H. Tang , X. Zhang , Y. Sun , Q. Wei , F. Wu , Y. Yuan , H. Deng , X. Zhou , J. Am. Chem. Soc. 2020, 142, 5049. 32069054 10.1021/jacs.9b10936

[531] Z. Q. Hu , J. W. Jiang , Microporous Mesoporous Mater. 2016, 232, 138.

[532] H. Zhang , Y. Lv , T. Tan , D. Van Der Spoel , J. Phys. Chem. B 2016, 120, 477. 26730607 10.1021/acs.jpcb.5b10437

[533] C. Caratelli , I. Hajek , E. J. Meijer , M. Waroquier , V. Van Speybroeck , Chem. ‐ Eur. J. 2019, 25, 15315. 31461187 10.1002/chem.201903178 PMC6916623

[534] P. Z. Moghadam , S. M. J. Rogge , A. Li , C.‐M. Chow , J. Wieme , N. Moharrami , M. Aragones‐Anglada , G. Conduit , D. A. Gomez‐Gualdron , V. Van Speybroeck , D. Fairen‐Jimenez , Matter 2019, 1, 219.

[535] V. Bon , E. Brunner , A. Pöppl , S. Kaskel , Adv. Funct. Mater. 2020, 30, 1907847.

[536] K. Tu , B. Puértolas , M. Adobes‐Vidal , Y. Wang , J. Sun , J. Traber , I. Burgert , J. Pérez‐Ramírez , T. Keplinger , Adv. Sci. 2020, 7, 1902897. 10.1002/advs.201902897 PMC7141016 32274302

[537] C. Wang , J. Kim , J. Tang , J. Na , Y.‐M. Kang , M. Kim , H. Lim , Y. Bando , J. Li , Y. Yamauchi , Angew. Chem., Int. Ed. 2020, 59, 2066. 10.1002/anie.201913719 31846187

[538] C. Wang , P. Cheng , Y. Yao , Y. Yamauchi , X. Yan , J. Li , J. Na , J. Hazard. Mater. 2020, 392, 122164. 32086095 10.1016/j.jhazmat.2020.122164

[539] Q. Zhang , M. Wahiduzzaman , S. Wang , S. Henfling , N. Ayoub , E. Gkaniatsou , F. Nouar , C. Sicard , C. Martineau , Y. Cui , G. Maurin , G. Qian , C. Serre , Chem 2019, 5, 1337.

[540] T.‐Y. Luo , C. Liu , X. Y. Gan , P. F. Muldoon , N. A. Diemler , J. E. Millstone , N. L. Rosi , J. Am. Chem. Soc. 2019, 141, 2161. 30636428 10.1021/jacs.8b13502

[541] Z. Ji , T. Li , O. M. Yaghi , Science 2020, 369, 674. 32764067 10.1126/science.aaz4304

[542] J. W. Jiang , Metal–Organic Frameworks: Materials Modeling towards Potential Engineering Applications, Pan Stanford Publishing Pte. Ltd., Singapore 2015.

